# Genome-wide sweeps create ecological units in the human gut microbiome

**DOI:** 10.1038/s41586-026-10476-w

**Published:** 2026-05-06

**Authors:** Xiaoqian Annie Yu, Cameron R. Strachan, Craig W. Herbold, Michaela Lang, Christoph Gasche, Athanasios Makristathis, Nicola Segata, Shaul Pollak, Adrian Tett, Martin F. Polz

**Affiliations:** 1 Division of Microbial Ecology, Centre for Microbiology and Environmental Systems Science, University of Vienna, Vienna, Austria; 2 Centre for Food Science and Veterinary Public Health, Clinical Department for Farm Animals and Food System Science, University of Veterinary Medicine Vienna, Vienna, Austria; 3 Austrian Competence Centre for Feed and Food Quality, Safety and Innovation FFoQSI, Tulln, Austria; 4 Te Kura Pūtaiao Koiora (School of Biological Sciences), Te Whare Wānanga o Waitaha (University of Canterbury), Ōtautahi (Christchurch), Aotearoa New Zealand; 5 Division of Gastroenterology and Hepatology, Department of Internal Medicine 3, Medical University of Vienna, Vienna, Austria; 6 Division of Clinical Microbiology, Department of Laboratory Medicine, Medical University of Vienna, Vienna, Austria; 7 CIBIO Department, University of Trento, Trento, Italy; 8 Division of Computational Systems Biology, Centre for Microbiology and Environmental Systems Science, University of Vienna, Vienna, Austria

**Keywords:** Evolution, Microbiome, Microbial ecology

## Abstract

The human gut microbiome is shaped by diverse selective forces that originate from host and environmental factors and it substantially influences health and disease. Whereas the association of microbial lineages with various health conditions has been shown at different taxonomic levels^1–5^, the extent to which unifying adaptive mechanisms sort microbial lineages into ecologically differentiated populations remains poorly understood. Here we show that genome-wide selective sweeps are a pervasive mechanism that differentiates bacteria in the microbiome. This mechanism leads to population structures akin to global epidemics across geographically and ethnically diverse human populations. Such sweeps arise when an adaptation allows a clone to outcompete others in its niche followed by rediversification, and they manifest as clusters of closely related genomes on long branches in phylogenetic trees. This structure is revealed by excluding recombination events that mask the clonal descent of the genomes. Indeed, we show that genome-wide sweeps originate under a wide range of recombination rates in at least 66 taxa from 25 bacterial families. Estimated ages of divergence suggest that sweep clusters can spread globally within decades and that this process has occurred throughout human history. Sweep clusters are associated with different host conditions—such as age, colorectal cancer, inflammatory bowel diseases and type 2 diabetes—as an indication of their ecological differentiation. Our results reveal an evolutionary mechanism for the observation of stably inherited strains with differential associations and provide a theoretical foundation for analysing adaptation among microbial populations.

## Abstract (web summary)

Genome-wide selective sweeps commonly occur in the human gut microbiome and can spread across the world within decades to produce epidemic-like population structures.

## Main

**Fig. 1:**
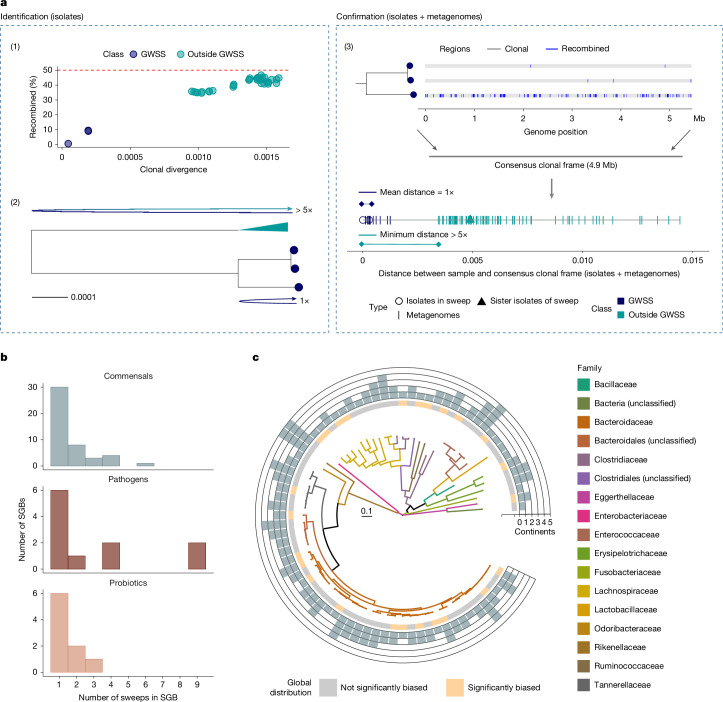
GWSSs are prevalent in the gut microbiome. **a**, Schematic of the pipeline used for the identification and confirmation of genome-wide sweeps using *Bacteroides intestinalis* (SGB1846) as an example. (1) Identification of GWSSs for clusters of genomes that have an average pairwise recombined portion of <50% and fulfil the 5× rule (defined as the divergence between the cluster and its sister clade exceeding 5 times the average divergence in the cluster). (2) A typical broom-like structure of GWSS clusters and illustration of the 5× rule. (3) Confirmation of GWSS clusters by first calculating the consensus clonal frame for each GWSS cluster, then mapping and calculating the distance of all samples (isolate genomes and metagenomes) to the consensus clonal frame, and finally checking adherence to a modified version of the 5× rule (within and between distances are calculated with reference to the clonal frame). The consensus clonal frame consists of the majority nucleotide at each site across the concatenated genome regions without evidence of recombination in any isolate of a GWSS cluster. **b**, Histograms of the number of GWSS clusters per SGB for commensal, pathogenic and probiotic bacteria. **c**, Phylogenetic and geographical distribution of GWSS clusters for commensal gut bacteria. The tree was constructed using approximately maximum-likelihood estimation based on a concatenated bac120 marker gene set in the GTDB-Tk database (R214)^44^ for all GWSS clonal frames. Branches of the tree are coloured according to bacterial families. The innermost circle represents whether samples in the GWSS had significantly biased continental occurrence relative to the geographical distribution of other samples in the SGB based on a two-sided Fisher’s exact test (*P* < 0.05). The height of the bars on the outer circles indicates the number of continents each sweep covered.

There is widespread agreement that the microbiome associated with our bodies heavily influences our wellbeing. Evidence for such assertions usually stems from correlations of some unit of bacterial diversity with disease or other dysbiotic states^1–5^. Such hypotheses can subsequently be tested in experimental models using animals colonized with isolates assumed to represent the identified bacterial group^6,7^. However, because correlations with host phenotypes are based on operationally defined units of microbial diversity, ranging from rRNA-level to strain-level variants^1–5,8,9^, it is difficult to ascertain whether these units and their associated model isolates accurately represent the adaptive process that underlies the association. A recently proposed path forward to address this issue is to define populations that are both ecologically and genetically differentiated from their sisters because they are the product of divergent environmental selection^10–13^. Termed ‘reverse ecology’, this approach has the potential to more precisely identify the genetic unit associated with host phenotypes because it is optimized by selection to occupy a particular niche space. Depending on the strength of selection relative to recombination, populations can be either optimized by genes or alleles sweeping across a population or by a genome-wide selective sweep (GWSS), whereby the entire genome hitchhikes with an adaptive mutation^14,15^. Theoretical considerations indicate that GWSSs are widespread; however, evidence for their occurrence in nature remains limited^16–18^. Nevertheless, it is notable that phylogenies of isolates originating from human microbiomes frequently manifest as ‘brooms’; that is, an expansion of closely related strains (broom head) on unexpectedly long branches (broom handle). This structure is a hallmark of GWSSs and arises when an adaptive mutation allows a strain to outcompete all sisters in their specific niche, followed by diversification of the winner into a cluster of closely related strains^19,20^ (Fig. 1a). These considerations therefore led us to propose that GWSSs are a common adaptive mechanism in the human microbiome.

To test the hypothesis that GWSSs create differentiated populations, we reasoned that it is necessary to constrain the effects of horizontal gene transfer and potential isolation bias among available isolate genomes. This constraint is required because both these effects can lead to the observation of long branches in phylogenetic trees^21^. Accordingly, we first sought to identify vertical descent among closely related genomes by differentiating clonally inherited and recombined portions of the genome. Next, we adopted a theoretical model that estimates the likelihood that genome clusters on long branches arise through GWSSs. Finally, we mapped metagenomes onto these clusters to test whether intermediate genotypes are missing from isolate collections. We also tested for signatures of selection and differential association with human phenotypes as a proxy for different ecological conditions in the gut. These analyses showed that GWSS clusters occupy different niche spaces. Moreover, many human microbiome taxa have been subject to GWSSs that can spread across the globe over decadal timeframes and lead to epidemic-like population structures.

## Genome-wide sweeps are common

**Extended Data Fig. 1:**
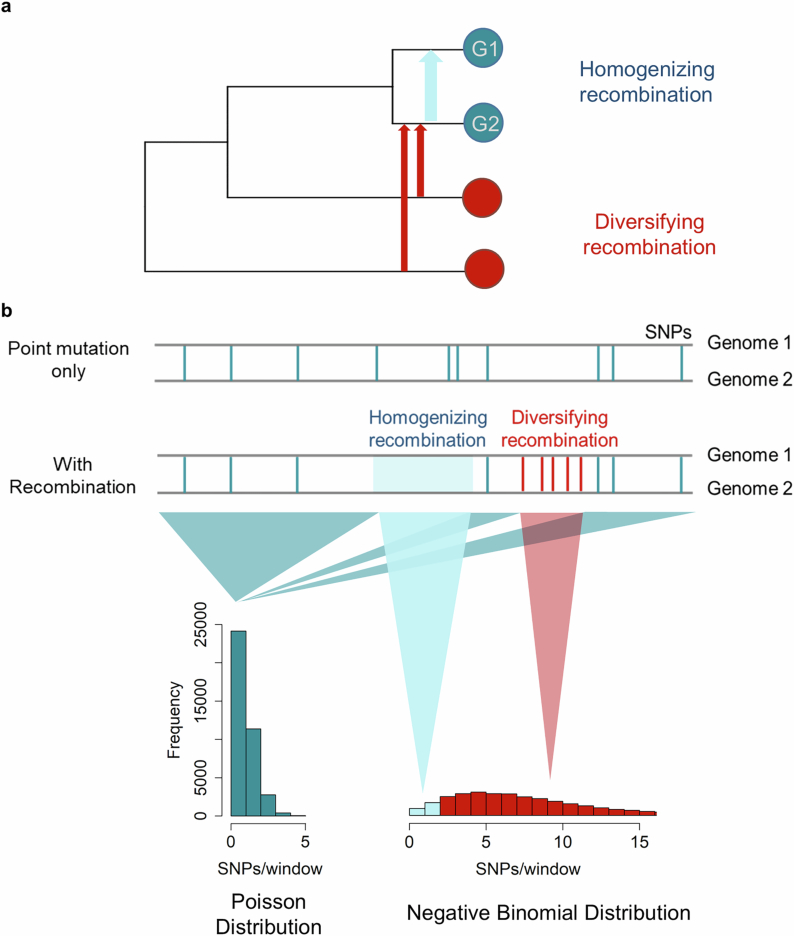
Single nucleotide polymorphism patterns across pairwise genome alignments reflect point mutations and recombination. **a**. Illustration of two types of recombination. For the focal pair of genomes (G1 and G2, dark cyan), recombination can happen between the two genomes (homogenizing recombination, light blue), or with phylogenetically more distant genomes (diversifying recombination, red). **b**. Recombination alters the SNP distribution between two genomes by either eliminating SNPs (homogenizing recombination, light blue) or by increasing the SNP density (diversifying recombination, red). The distribution of the number of SNPs in sliding windows across pairwise genome alignments can be expressed as the fractional sum of a Poisson distribution (point mutations), and a Negative Binomial distribution that accounts for both diversifying and homogenizing recombination.

**Extended Data Fig. 2:**
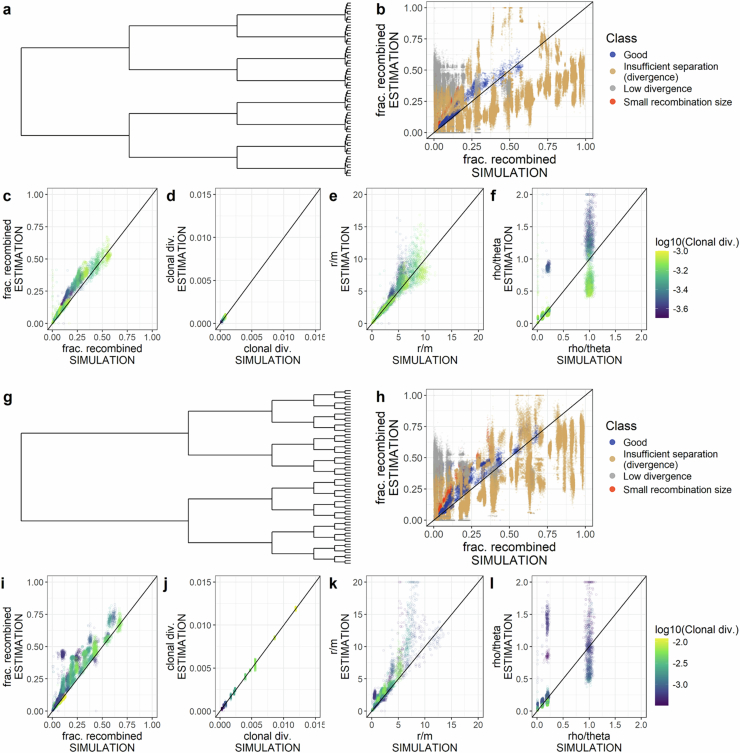
Validation of the mixture model for recombination estimation with simulated data. **a**. Phylogenetic structure of sets of 64 simulated genomes evolved along a phylogenetic tree with sweep clusters under 144 parameter combinations (Methods). **b**. Comparison of the recombined genome fraction in the simulation and estimated by the model. Different colors represent categories of genome pairs where the model accurately estimates the recombined genome fractions (dark blue), or is insufficient due to different reasons: yellow, insufficient differentiation of the clonal and recombined fragments due to similar divergences (< 2.5-fold difference); grey, overall divergence too low (< 0.0075%); red, insufficient length of recombination fragments (exponential distribution with a mean of 200 base pairs). **c-f**. Comparison between simulated vs. estimated values within the functional range of the model for recombined genome fraction, clonal divergence, the effective recombination rate r/m, and relative recombination to mutation occurrence rate rho/theta. **g-l**. same as a-f but for sets of 64 simulated genomes with balanced phylogenetic tree structure.

The process of diversification after a GWSS creates the characteristic broom-like structure on phylogenetic trees of isolates; that is, the genomic diversity in a niche is eliminated and subsequently rediversifies. However, the confident identification of such sweep clusters requires that the influence of recombination and isolation bias be constrained. Because homologous recombination can obscure clusters and generate long branches, especially among closely related genomes^21^, we first sought to identify the clonally inherited portion among genome pairs. To this end, we built on previous approaches that partition the single-nucleotide polymorphism (SNP) distribution between pairs of genomes into a mixture of a Poisson and a negative binomial distribution^10,22^. The former distribution captures the accumulation of point mutations in clonal genome regions, whereas the latter models the effect of recombination, which can either increase or reduce SNP density depending on the phylogenetic distance of the source genome (Methods and Extended Data Fig. 1). By determining the best fit for the observed SNP distribution to a mixture of these two models, we estimated the following features: (1) the fraction of the genome that is vertically inherited versus recombined; (2) the divergence in each fraction; and (3) the recombination rates between pairs of genomes. We comprehensively assessed this approach using simulated sets of genomes evolved from a single ancestral genome while incorporating selection and recombination under a wide range of mutation and recombination rates. We also defined the regime for which recombination can be confidently differentiated from point mutations (Methods and Extended Data Fig. 2), and we found that this divergence regime occurred at the subspecies level. Therefore, we delineated species-level genome bins (SGBs) as groups of genomes that are <6% diverged from a reference genome in the MetaPhlAn4 database^23^ and applied the mixture model to each SGB separately. We used a dataset of 16,864 human gut isolate genomes, which we dereplicated according to human participants to avoid double counting of clonal isolates from the same individual owing to repeated sampling (Methods). This categorization resulted in 176 SGBs that contain more than 5 genomes, which showed widely varying fractions of clonal and recombined genome portions. This result suggests that the diversity in SGBs is differentially structured by vertical and horizontal inheritance (Supplementary Fig. 1).

To test for the occurrence of GWSSs, we first analysed the isolate genomes that comprise the 176 SGBs. Subsequently, we used metagenomes to confirm the observed structure and account for potentially missing diversity from the isolate collections. We developed the computational pipeline ‘populations as clusters of genome sweeps’ (PopCoGenomeS)^24^ to search the 176 SGBs for groups of genomes that were vertically inherited from a common ancestor and displayed hallmarks of GWSSs. We tested whether there is support for GWSSs by applying a coalescent-based model (5× rule) that compares the diversity in a cluster of genomes to its closest relative^25,26^ (Fig. 1a). If the ratio of between to within cluster divergence is >5, the probability that an identified GWSS is in fact due to evolutionary drift, which is the most likely alternative mechanism to selection for the generation of sequence clusters, is <1% (Methods). In this way, we identified 377 putative GWSS clusters with many SGBs that contained multiple clusters. We then asked whether this structure can be confirmed through whole community metagenomic analysis that is not subject to isolation bias. To this end, we compiled 1,477 metagenomes from 74 datasets covering different ages, disease states and biogeographical regions (Methods). We first determined a majority-rule consensus sequence for each GWSS cluster, which is the majority nucleotide in each position of the genome not affected by recombination (consensus clonal frame; Fig. 1a). Then we calculated the distance of each of the 1,477 metagenomes and all isolate samples to this consensus clonal frame for the confirmation of GWSS clusters that adhere to the 5× rule (Fig. 1a and Methods).

In this combined isolate genome and metagenomic dataset, we were able to confirm 124 GWSSs in 66 SGBs. These SGBs consisted of 46 commensals, 11 pathogens and 9 commensals that are frequently found in fermented and functional foods (probiotics) (Fig. 1b and Supplementary Tables 1 and 2; see Methods for SGB classification). Although most SGBs contained a single GWSS cluster, many contained multiple clusters. This finding indicates that species that are operationally defined by average nucleotide identity can consist of several evolutionarily defined units of diversity, which, based on the evidence presented below, display differential ecology. We next specifically focused on commensals for which the identification of sweeps is less likely to be affected by disease outbreaks or the widespread use of probiotics, and identified a total of 77 GWSSs in 46 SGBs spanning 17 bacterial families. GWSSs were most prevalent in Bacteroidaceae and also present in most of the other major human gut bacterial families such as Clostridiaceae, Lachnospiraceae and Enterococcaceae. This finding indicates that GWSSs are a pervasive mechanism that differentiates populations in the human microbiome (Fig. 1c). As the number of sweeps detected in each SGB significantly correlated with the total number of samples for which the SGB was detectable (Spearman’s test, *n* = 46, *ρ* = 0.46, *P* = 0.001), it is likely that GWSSs exist in other gut bacterial families that have been less extensively cultured.

**Extended Data Fig. 3:**
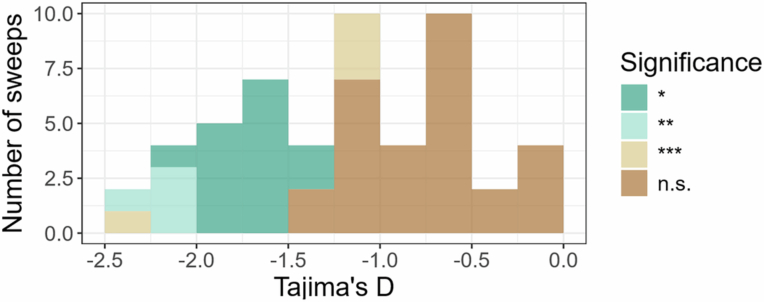
Population genomic signatures of genome-wide selective sweep clusters. Histogram of the Tajima’s D for all GWSS clusters detected in commensal gut bacteria. ***P < 0.001, **P < 0.01, and *P < 0.1. Significance was assessed using a two-sided parametric test of deviation from the standard neutral model using the beta approximation of the sampling distribution of D.

Further support for the idea that GWSS clusters are selectionally optimized units of diversity arises from population genetic metrics. Under population genetic theory, Tajima’s *D* quantifies the average pairwise nucleotide diversity relative to that expected under a neutral model. This model assumes that SNPs are randomly distributed among genomes at a constant mutation rate. Negative values indicate an excess of rare alleles, as expected in populations that have recently undergone a selective sweep or experienced a population bottleneck^27^. Indeed, all commensal GWSSs identified using the 5× rule also had negative Tajima’s *D* values (Extended Data Fig. 3). This result provides additional support for the fact that GWSSs are the result of recent clonal expansions owing to selection. Consequently, we investigated the geographical extent of GWSSs as further evidence for their adaptive nature.

Extreme and persistent bottlenecks could lead to the erroneous inference of a GWSS. However, the likelihood of this scenario becomes diminishingly small if GWSS clusters are widely distributed among human populations. Here we found that most GWSS clusters (54 out 77) comprised isolates or metagenomes from more than one continent, which indicates that they have spread among different human populations (Fig. 1c). Among these 54 widespread clusters, 42 did not show spatial preference (Fisher’s exact test, *P* > 0.05 for the geographical distribution of samples inside and outside a GWSS cluster; Fig. 1c), which suggests that they respond to similar selective pressures in diverse human populations. Notably, one *Bacteroides uniformis* sweep cluster was unique to indigenous populations and was shared among three populations on two different continents: the Baka and Beti people in Cameroon^28^, and the Matses people in Peru^29^. Because the Baka and the Matses are geographically remote hunter–gatherer communities who have never been in direct contact with each other, the spread of the same sweep cluster among them is best explained by a chain of transmission events mediated by less isolated populations. The absence of other human populations from this GWSS cluster suggests two possibilities. First, these strains are present at very low abundance in other modern-day humans but are selectively enriched only in certain traditional lifestyles. Alternatively, the transmission occurred in the past, and the strains have since gone extinct in most human populations, possibly due to lifestyle shifts related to industrialization and urbanization. These observations of global distributions of GWSS clusters led us to investigate the approximate timescales over which sweeps may occur. This question was of particular interest to answer because rapid global spread would provide further support that GWSS clusters are the product of selection rather than population bottlenecks and drift.

## Sweep clusters spread rapidly

**Fig. 2:**
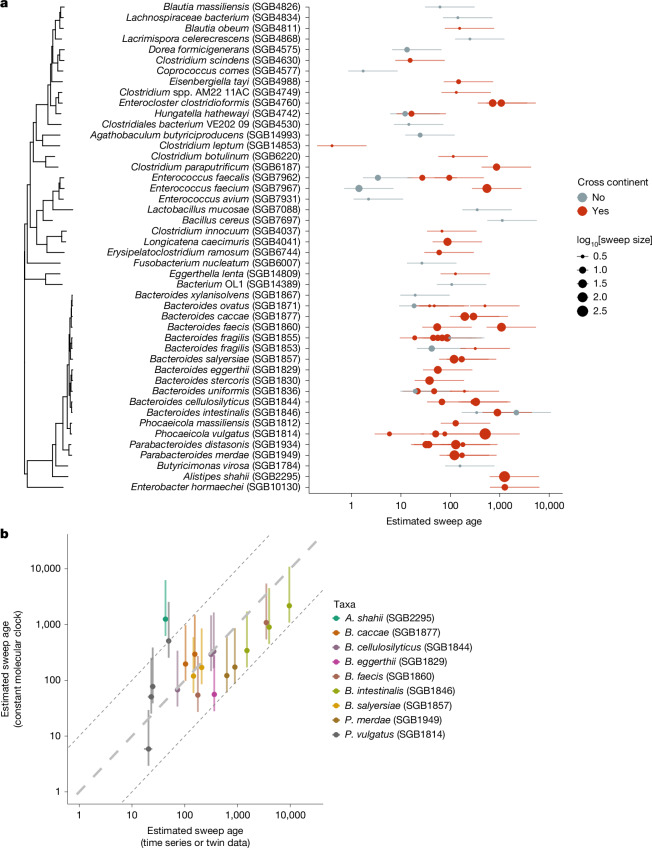
GWSSs arose repeatedly and spread rapidly. **a**, Estimated age of GWSS clusters (dots) using a constant molecular clock with a median mutation rate of five SNPs per genome per year, with the size of the dot representing the number of samples (isolate genomes and metagenomes) in each GWSS cluster. The error bars for each dot represent the upper and lower limits of the age of each sweep cluster at one and ten SNPs per genome per year, respectively. The colour of the dots indicates whether a sweep cluster was detected across more than one continent. The approximately maximum-likelihood tree on the left is based on a concatenated bac120 marker gene set (GTDB-Tk database, R214)^44^ extracted from a randomly selected GWSS clonal frame in each SGB. **b**, Comparison of the estimated ages of sweep clusters using a constant molecular clock (five SNPs per genome per year) and metagenomic molecular clocks determined separately for each SGB. The grey dashed line in the middle represents a 1:1 ratio between the two estimates, whereas the two grey dashed lines on the sides represent 10:1 and 1:10 ratios. The horizontal error bars represent the standard error of the estimated age from metagenomes, whereas the vertical error bars represent the upper and lower limits of the sweep age under a constant molecular clock (one and ten SNPs per genome per year).

Using two independent methods to constrain the age of sweep clusters, we found that GWSSs have occurred repeatedly over the course of human history. In detail, we first used a previously determined molecular clock rate for commensal and pathogenic bacteria of 1–10 mutations per genome per year^30–32^ to estimate that the age of sweep clusters ranged from approximately tens to thousands of years (Fig. 2a). These age estimates were consistent with those obtained using a second independent method of estimating molecular clocks. In this approach, we determined the number of substitutions that have accumulated in the same GWSS clusters detected in metagenomes of individuals who were sampled at least twice with a time interval of >1 year or twins who have resided apart since reaching adulthood (Methods). With only two exceptions, the age of sweep clusters estimated from these metagenome-based evolutionary clocks was in the same order of magnitude as those estimated using the fixed molecular clocks of 1–10 SNPs per genome per year (Fig. 2b). Although such estimates carry obvious uncertainties, our focus was on estimating sweep ages to the correct order of magnitude. This is because the estimation of precise rates was limited by the availability of metagenome and isolate-based mutation rates for gut commensal bacteria. Nevertheless, these estimates suggest that GWSSs have happened repeatedly over the past thousands of years, with clusters showing nearly a 1,000-fold difference in age. Moreover, even within a taxonomic species (SGB), sweep clusters with orders of magnitude different ages can exist (Fig. 2a).

**Extended Data Fig. 4:**
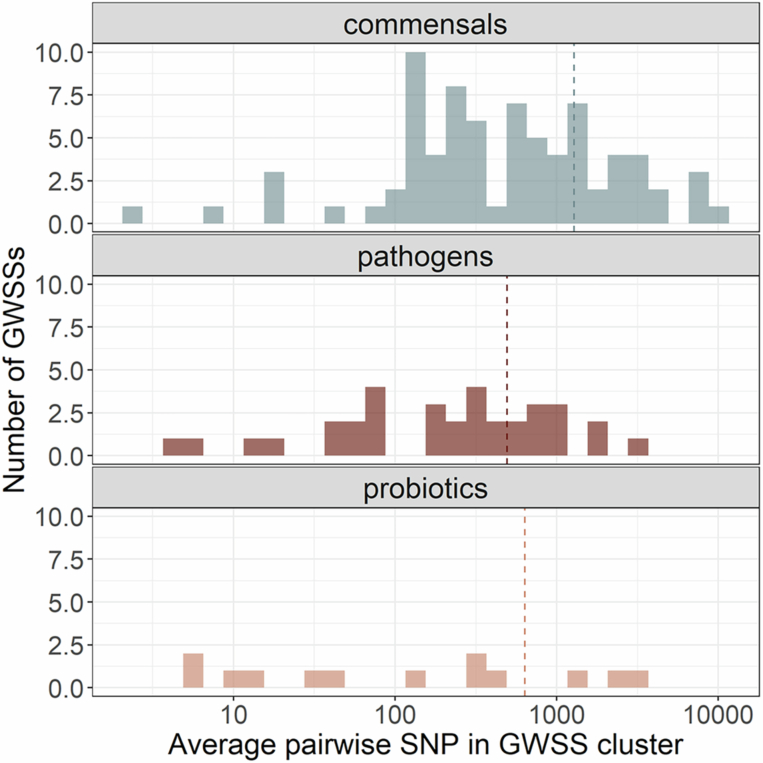
Average SNP distance within genome-wide selective sweep clusters for commensal, pathogenic and probiotic bacteria. Histograms show the distribution of average SNP distances within GWSS clusters for each bacterial category, with the dashed line indicating the mean of the distribution. See Methods for how SGBs are assigned to the three different categories.

The age of sweep clusters provides an upper bound on the time required for their spread into geographically distant human populations. Therefore, we used this information to estimate the minimum time for transmission across continents. Using the median molecular clock of 5 SNPs per genome per year, a total of 26 multicontinental sweep clusters were estimated to be younger than a century (Fig. 2a). Furthermore, in the majority (47 out of 54) of multicontinental sweep clusters, the genetic distances of samples that originated from different continents were not significantly different from those from the same continent (ANOSIM test, *P* > 0.1). This result suggests that in most SGBs, there were rapid and repeated cross-continental transfers of strains. Notably, the cross-continental *B. uniformis* sweep unique to the three indigenous and reputedly isolated populations mentioned above was the oldest GWSS in *B. uniformis*, with an estimated age of approximately 100–1,000 years. This result provides support for the idea that this cluster may have spread before industrialization. Finally, the average SNP diversity in sweeps for commensal bacteria was significantly larger than that for pathogenic bacteria (two-tailed Student’s *t*-test on log-transformed data, *n* = 77 and 34, d.f. = 63, *P* = 0.018, Extended Data Fig. 4). Therefore, the transmission of commensal bacteria in the human population was slower and/or the sweep clusters are older than infectious pathogen outbreaks. Nonetheless, our data indicate that even commensal GWSS clusters can spread globally within the lifespan of a human and that their distribution is consistent with shared selective regimes in diverse human hosts.

## GWSSs span diverse recombination rates

**Fig. 3:**
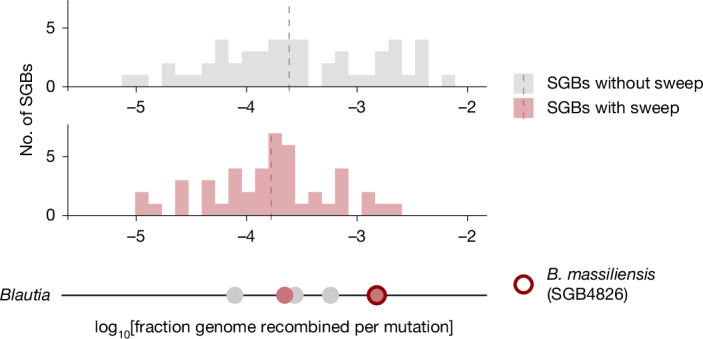
GWSSs occur across a wide range of recombination rates. Top and middle, comparisons of recombination rates between SGBs without (top) and with (middle) detected GWSSs. The dashed line in each histogram indicates the median of the distribution. Bottom, SGBs from the *Blautia* genus are shown as a representative example to illustrate that SGBs with GWSSs do not necessarily have lower recombination rates. Each dot represents an SGB aligned to the histograms above according to its recombination rate.

Theory predicts that GWSSs occur when the entire genome hitchhikes with an adaptive mutation. Thus, the relative rate at which the genome carrying the adaptation spreads in its niche must be much higher than that of the adaptive gene or allele being shared through recombination^14,15^. This reasoning stems from the fact that high recombination rates can lead to gene-specific sweeps by breaking the linkage between the beneficial mutation and its neighbouring variants, allowing the adaptive allele to spread independently of its genomic background. However, contrary to this explicit expectation, we found that SGBs with no confirmed GWSSs had comparable recombination rates (genome fraction recombined per mutation) to those containing GWSSs (median 2.4 × 10^−4^ compared with 1.7 × 10^−4^; Wilcoxon rank-sum test, *n* = 52 and 45, respectively, *P* = 0.09; Fig. 3 and Supplementary Fig. 2). In fact, among commensals, GWSSs were detected across a range of recombination rates that varied by approximately 400-fold. For example, *Blautia massiliensis* (SGB4826) has a sweep despite being among the fastest recombining SGBs, whereas multiple other SGBs from the same genus have recombination rates that are 3–20-fold lower with no observed sweeps (Fig. 3). Our data therefore suggest that selection is frequently strong enough to permit GWSSs even under high recombination regimes.

## GWSS clusters are ecological populations

**Fig. 4:**
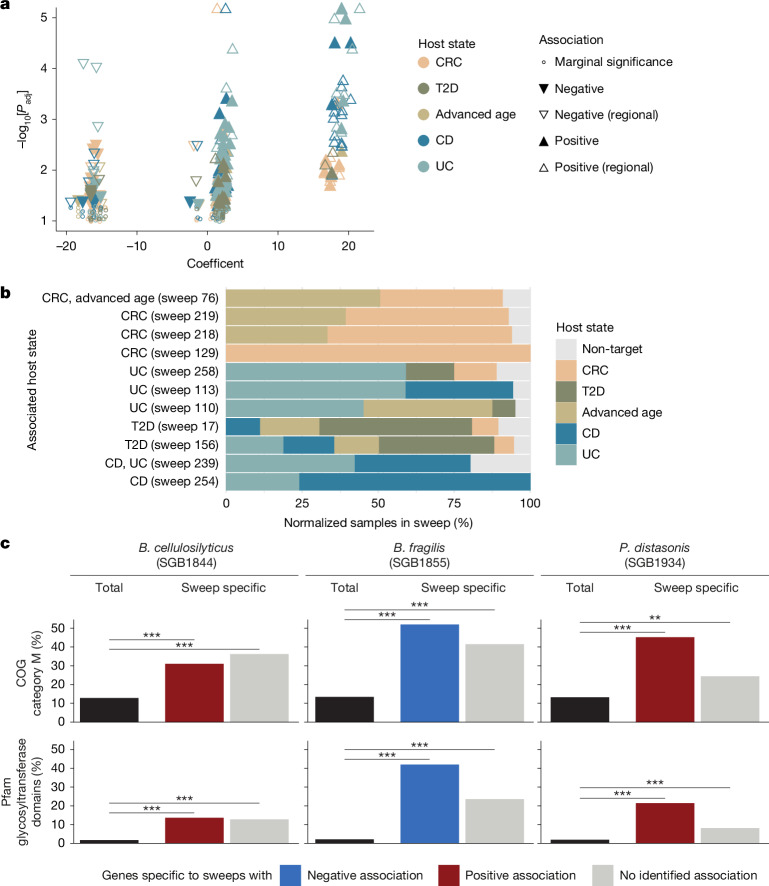
Differential ecological associations of GWSS clusters in SGBs. **a**, Volcano plot showing associations between GWSS clusters and five host phenotypes. Each point represents a GWSS cluster; the *x* axis shows the regression coefficient and the *y *axis shows –log_10_[*P*_adj_] values. Colours denote the associated disease or host condition. Upward and downward triangles indicate GWSS clusters significantly positively or negatively associated with the disease (*P*_adj_ < 0.05), whereas circles indicate marginal associations (0.05 ≤ *P*_adj_ < 0.1). Open and filled triangles distinguish associations with and without regional bias, respectively. Associations were tested using general linear models; tests were two-sided and *P* values were adjusted using the Benjamini–Hochberg procedure. Regional bias was assessed using two-sided chi-squared goodness-of-fit tests in each GWSS cluster; GWSS clusters with *P* < 0.05 were classified as regionally biased. **b**, Stacked bar plots summarizing the host conditions positively associated with GWSS clusters in *B. uniformis* (SGB1836). Each row represents a GWSS cluster; row labels indicate associated host conditions (*P*_adj_ < 0.05, no regional bias; same statistical framework as in **a**). The coloured segment length represents enrichment of samples from each host condition in a GWSS cluster relative to their frequency in the entire SGB, with segment colours denoting the respective conditions. The distribution of host conditions in all GWSS clusters positively associated with a condition is shown in Extended Data Fig. 6. **c**, Functional enrichment of genes specific to GWSS clusters in three Bacteroidales SGBs. Bar plots show the percentage of genes annotated with clusters of orthologous group (COG) category M (cell wall, membrane, envelope biogenesis; top) and Pfam glycosyltransferase domains (bottom) among all GWSS genes (total) and those unique to single GWSSs (sweep specific). Bars are coloured by disease-association status (blue, negative; red, positive; grey, none). Significance was determined using one-sided Fisher’s exact tests (*P < 0.05, **P < 0.005, ***P < 0.0005).

**Extended Data Fig. 5:**
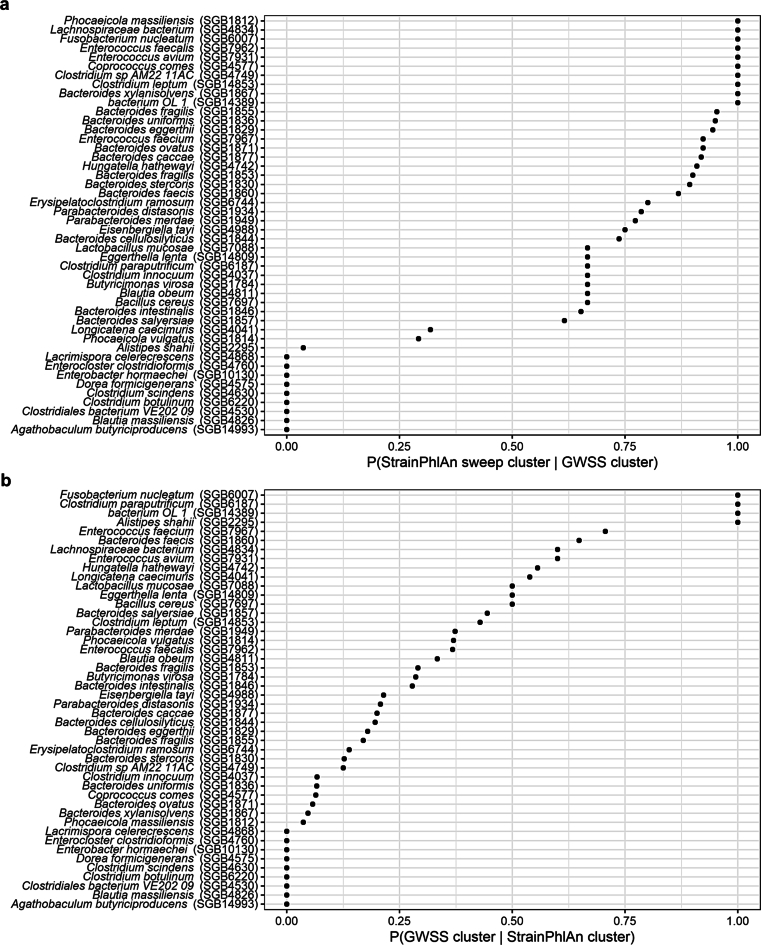
Comparison of genome-wide sweep clusters identified using isolate-based clonal frames and StrainPhlAn marker genes for each of the 46 commensal SGBs. **a**. The probability that samples (including isolate genomes and metagenomes) within GWSS clusters identified using isolate-based clonal frames also fall within sweep clusters called from the StrainPhlAn marker gene trees. **b**. The probability that samples (including isolate genomes and metagenomes) within sweep clusters called from the StrainPhlAn marker gene trees, also fall within GWSS clusters identified using isolate-based clonal frames.

A key prediction of the theory underlying GWSSs is that sweep clusters are ecologically differentiated. Although fine-scale niche differences in the gut are difficult to constrain, we proposed that major life-history or physiological changes in humans may lead to shifts in ecological conditions that bacterial populations respond to. As a test case, we searched for associations between GWSS clusters and five disease phenotypes that are well represented in datasets across different human populations: colorectal cancer (CRC), Crohn’s disease (CD), ulcerative colitis (UC), type 2 diabetes (T2D) and advanced age (>65 years old). We extended the 5× rule to phylogenetic distances based on alignments of metagenomes to marker genes in the MetaPhlAn database as this approach enables more efficient scaling up to large datasets (Methods and Extended Data Fig. 5). We were therefore able to expand the screening for GWSS clusters to a collection of 6,783 metagenomes from 29 datasets covering various geographies (Methods). Using a general linear model with a forward, stepwise variable selection approach, we identified a total of 178 commensal GWSS clusters that were significantly associated with at least one host phenotype of interest (Benjamini–Hochberg procedure, adjusted *P* value (*P*_adj_) < 0.05), of which 104 span across multiple datasets with no significant geographical bias (chi-squared test in each GWSS, *P* > 0.05, Fig. 4a).

**Extended Data Fig. 6:**
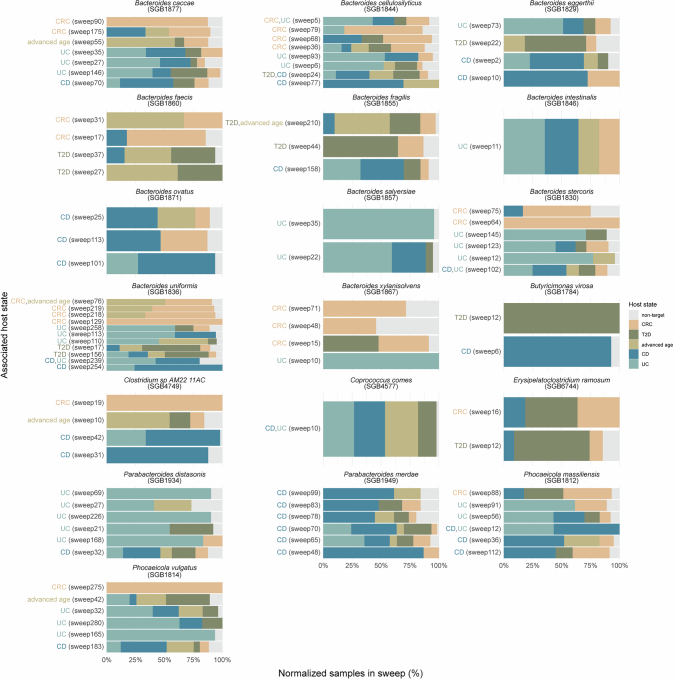
Stacked bar plots summarizing host conditions associated with GWSS clusters in all commensal SGBs. Each row represents a GWSS cluster within a SGB and is labeled according to the major host condition(s) associated with that cluster (*P*_adj_ < 0.05, no regional bias). Associations were tested using general linear models. All tests were two-sided, and *P* values were adjusted using the Benjamini–Hochberg false discovery rate (FDR) procedure. Regional bias was assessed using two-sided chi-squared goodness-of-fit tests within each sweep; sweeps with *P* < 0.05 were classified as regionally biased. Within each row, the length of each colored segment represents the enrichment of samples from different host conditions in a given GWSS cluster, relative to their occurrences in the entire SGB, with segment colors denoting the respective conditions.

**Extended Data Fig. 7:**
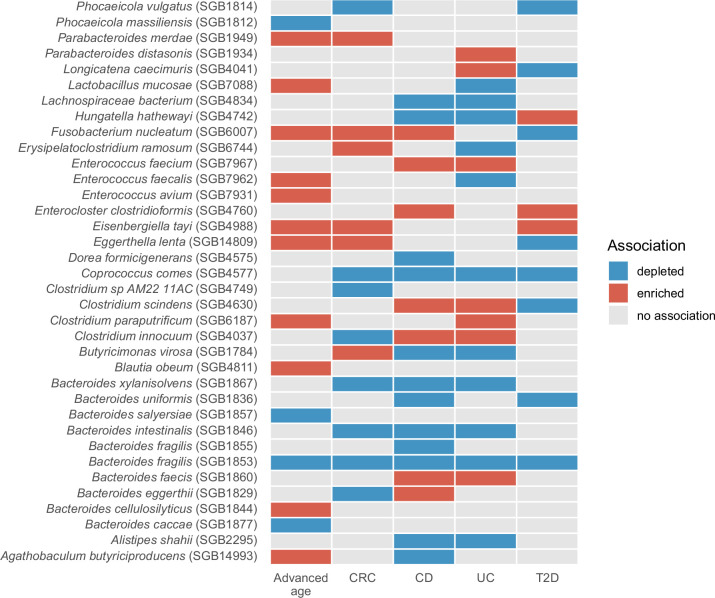
SGBs associated with host conditions. Heatmaps of statistically significant (*P*_adj_ < 0.05) associations between entire SGBs and host conditions. Associations were tested using general linear models. All tests were two-sided, and *P* values were adjusted using the Benjamini–Hochberg false discovery rate (FDR) procedure.

In line with the idea that GWSS clusters are finely differentiated ecological units, clusters in the same SGB can exhibit distinct associations with different host phenotypes. Notably, positive associations were generally more significant than negative ones, which suggests that disease-specific selective pressures may be stronger than those associated with broader non-disease states (Fig. 4a). We identified 14 SGBs with GWSS clusters positively associated with multiple host conditions, 11 of which belonged to the order Bacteroidales (Extended Data Fig. 6). These SGBs generally contained high numbers of sweep clusters, each with limited prevalence, which suggests that there is fine-scale and frequent differentiation in this order (Fig. 2a). A representative example is *B. uniformis*, which exhibited positive associations with all five host phenotypes (Fig. 4b) despite showing either no or even negative associations with these phenotypes at the SGB level (Extended Data Fig. 7). It was also one of the SGBs with a large number of confirmed sweeps (four GWSS clusters; Fig. 2a), with the average sweep cluster comprising only 5 ± 1.8 samples. This finding indicates that there is differential selection among populations in the same taxonomic species and is consistent with the ability of strains possessing a selective advantage to rapidly occupy a niche, the breadth of which constrains the extent of occurrence of the sweep cluster.

**Extended Data Fig. 8:**
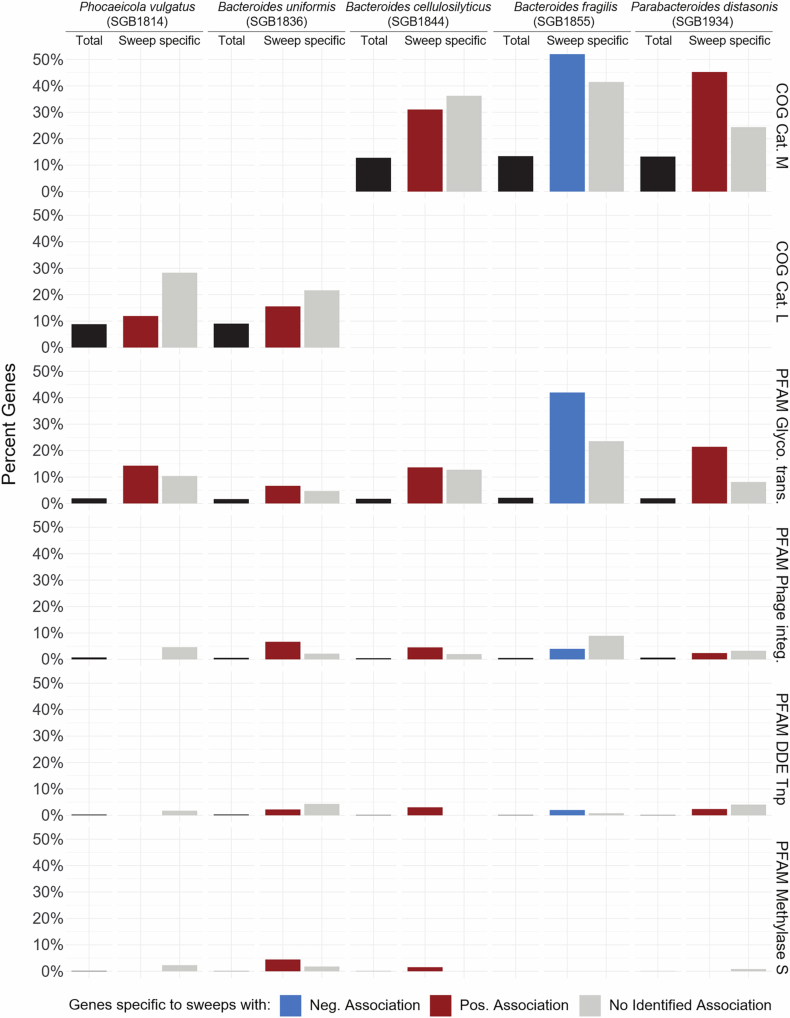
Functions enriched in genes specific to GWSS clusters in all SGBs. Bar plots show the percentage of genes annotated with selected COG categories and Pfam domains among all genes within GWSS clusters (“Total”) and among genes unique to GWSS clusters (“Sweep specific”) for each SGB. Only COG categories and Pfam domains in SGBs where the difference between “Total” and “Sweep specific” genes was statistically significant (*P* < 0.000005) are shown, i.e. COG category M (Cell wall/membrane/envelope biogenesis) and COG category L (replication, recombination, and repair). Bars are colored by the disease association status of each GWSS cluster: blue for negative associations, red for positive associations, and gray for no association.

To explore potential functions that underlie such niche differentiation, we used all isolate-based sweeps and asked which genes might be specific to or are under positive selection in each GWSS cluster (Methods and Supplementary Table 3). Compared with the overall distribution of all genes in GWSS clusters, genes specifically enriched in individual GWSS clusters were categorized as mobile genetic elements and had functions involved in the synthesis or modification of glycans and glycoconjugates, such as capsular polysaccharides (Extended Data Fig. 8), especially in Bacteroidales (Fig. 4c). As these structures have long been known to contribute to pathogenicity by allowing pathogens to escape specific immune responses^33–35^, it is possible that commensal bacteria use similar mechanisms to adapt to discrepant host phenotypes related to health or disease. Indeed, specific capsular polysaccharides in *Bacteroides thetaiotaomicron* facilitate evasion of the adaptive immune system^36^, whereas those in *Bacteroides fragilis* can confer protection against inflammatory diseases^37^. Moreover, genes that regulate capsular biosynthesis in *B. fragilis* have been inferred to be under adaptive evolution in individual human hosts^31^. Altogether, our results suggest that GWSS clusters in commensal bacteria can display highly specific associations with human phenotypes, and their population structure may be influenced by interactions with the immune system.

## Discussion

Our findings indicate that GWSSs are a fundamental force that structures bacterial populations in humans. Furthermore, we suspect that they occur more commonly than our results show because our analysis was limited by the availability of sets of closely related isolate genomes. Because GWSSs create ecologically differentiated populations, their mapping onto metagenomes may serve as accurate and easily definable markers for various health conditions. Among SGBs, we identified GWSS clusters that are differentially associated with human phenotypes such as CRC, CD, UC, T2D and advanced age (>65 years old). A further important corollary of their ecological differentiation is that GWSS clusters should be adapted to coexist with at least a subset of other community members. Therefore, the identification of GWSS clusters may facilitate the construction of bacterial co-occurrence networks and could affect the choice of appropriate isolates to serve as experimental models or the assembly of synthetic gut communities.

An unexpected outcome of our analysis is that GWSS clusters of gut commensals seem to be globally adaptive and spread in a fashion that resembles epidemics, which are typically ascribed to pathogens^38,39^. However, the underlying mechanisms involved in the spread of commensal and pathogenic bacteria are likely to be different. For many pathogens, it is well known that novel adaptive clones can spread rapidly and lead to genetically highly homogeneous global populations. By contrast, our data suggest that commensals may require decades to reach global distribution, which may be due to slower transmission chains. This slower spread is reflected in the higher diversity of commensal GWSS clusters because they may diversify as they spread geographically. Strains, which are often regarded as the lowest identifiable unit of diversity in metagenomes, have been shown to be primarily acquired from related individuals^40^, but strain sharing among unrelated individuals has been observed at both local and global scales^40–42^. Moreover, although the level of sharing is lower than among related individuals, it remains substantially higher than expected under neutral simulations^42,43^, which suggests that it is driven by broader population genetic processes rather than random dispersal. However, the eco-evolutionary mechanism behind this phenomenon remains unclear, despite suggestions that host adaptation has an important role^41^. In the model proposed here, not all closely related strains among unrelated individuals in an SGB originate from the same clonal expansion^42^. However, a portion can be part of GWSS clusters and their acquisition reflects primarily their enhanced adaptation to the specific settings in a microbiome. Indeed, once acquired, strains have been shown to persist over extended periods of time^5^, on average over one-third of the human lifespan^40^. Therefore, we suggest that their replacement also reflects the turnover of ecologically adapted populations, which may also serve as a sentinel for shifts in human health.

## Methods

### Isolate genome collection

A total of 19,837 publicly available isolate genomes were collected and consisted of the entire data in the Unified Human Gastrointestinal Genome (UHGG) catalogue^45^ (v.1.0, 10,648 genomes) and 4 large-scale culturomic studies^28,46–48^. Furthermore, a group of 186 isolates from Austrian individuals were newly collected and sequenced. A total of 50 patients undergoing CRC screening colonoscopy at the Vienna General Hospital were enrolled in the study, which comprised 24 individuals with irritable bowel syndrome, 5 with UC and 21 healthy participants. None of the participants were found to have carcinomas during the colonoscopy.

For bacterial isolation of participants in the Austrian study, brush samples and biopsy samples were collected during colonoscopy from the ileal or caecal mucosa or the ascending colon with or without an endoscopically visible biofilm and were immediately processed^49^. Brush and biopsy samples were vortexed or homogenized in 0.6 ml of 0.9% NaCl, and the suspensions were subsequently plated on one of the following six culturing conditions: Columbia agar with 5% sheep blood, MacConkey agar, Columbia CNA agar with 5% sheep blood or CPS agar (Becton Dickinson) under aerobic conditions at 37 °C; Brucella agar with 5% horse blood or Schaedler KV agar with 5% sheep blood (Becton Dickinson) under anaerobic conditions at 37 °C. Aerobic cultures were assessed after 18 h and 48 h, and anaerobic cultures were assessed after 48 h and 72 h. Colonies identified as *Bacteroides* or *Parabacteroides* by matrix-assisted laser desorption ionization time-of-flight mass spectrometry analysis on a MALDI Biotyper MBT smart instrument (Bruker) or by 16S rRNA gene sequencing on a capillary sequencer (SeqStudio Genetic Analyzer, Applied Biosystems by Thermo Fischer Scientific) were saved as glycerol stocks at −80 °C.

Glycerol stocks of *Bacteroides* and *Parabacteroides* were cultured in brain heart infusion medium with supplements for 24 h before DNA isolation. DNA isolation was performed on a King Fisher Flex instrument (Thermo Fisher Scientific) using a MagMA DNA Multi-Sample Ultra 2.0 kit (Thermo Fisher Scientific), which included an initial proteinase K digestion step and a RNase treatment step. Sequencing libraries were prepared using a NEBNext Ultra II FS DNA Library Prep kit, with NEBNext Multiplex Oligos for Illumina barcodes. Sequencing was performed on an Illumina NovaSeq 6000 platform using SP flow cells (300 cycles, 2 × 150 bp paired-end reads). Reads were trimmed, filtered and merged with BBMap^50^ (v.38.90; ktrim=r k=21 mink=11 hdist=2 minlen=125 qtrim=r trimq=15), and de novo genome assembly was performed using Spades (v.3.15.5)^51^ under isolate mode.

### Isolate quality filtering and taxonomic assignment

A total of 20,023 genomes were collected and evaluated using CheckM (v.1.2.2)^52^ to screen for genomes that met the following criteria: >85% genome completeness, <5% contamination and N50 > 50 kb. This selection process produced a total of 16,864 genomes that were of sufficient quality for downstream analyses. We assigned each genome to a SGB according to the MetaPhlAn4 reference genome database (v.Jan 2022)^23^. Each genome was assigned to the SGB it was most closely related to based on FastANI (v.1.33)^53^, with the centroid genome as the primary reference or, if unavailable, a representative genome. To account for potential mis-assignments in species with boundaries slightly lower than the commonly used threshold (95%)^54^, we adopted a more relaxed species boundary of 94% average nucleotide identity (ANI) for SGB assignment. Specifically, SGB assignments were only made for genomes that were less than 6% divergent from at least one reference genome with over 30% sequence alignment. As a result, the total divergence in each SGB could be as high as 10–12%. Genomes failing to meet the 94% ANI cutoff with their closest relatives were converted to synthetic fastq reads (ART-2016.06.05, -ss HS25)^55^ and assigned by MetaPhlAn4 (v.4.0.3)^23^. Genomes that could not be assigned by either method were excluded. We further checked whether the ANI-based and MetaPhlAn4-based SGB assignments are generally consistent by converting a random subset of genomes in each ANI-based SGB to synthetic fastq reads (ART-2016.06.05, -ss HS25)^55^ and assigning them by MetaPhlAn4. Although the majority of ANI-based and MetaPhlAn4-based SGB assignments were congruent, certain ANI-based SGBs had all genomes assigned to another SGB in MetaPhlAn4. All genomes in these SGBs were reassigned to their corresponding MetaPhlAn4-assigned SGB (Supplementary Table 4).

### Isolate genome filtering based on metadata

A key step to ensure unbiased genome-wide sweep identification is to filter the genomes so that each SGB only contains isolates that originate from different individuals. For each isolate, we retrieved information on human participant identifiers, age, sex, health status, country, year and creator of collection, and BioProject accession number from the UHGG database^45^, as well as from the text or supplementary materials of the respective publications. For each SGB, we only retained isolates that originated from a different human participant based on either a unique identifier, country of sample or BioProject number (representing a different study; studies with multiple BioProject numbers were checked for and manually corrected). For studies with more than ten genomes but no human participant identifier or country information, we created a human participant identifier as a combination of the following five factors: age (or age group when the exact age was not available), sex, health status of the participant, year and creator of collection. Human participants with different combination-based identifiers were considered as different individuals. When multiple genomes from a single SGB were isolated from the same individual, we chose the genome with the highest quality score according to dRep (v.3.4.1)^56^. This procedure resulted in a final collection of 6,411 high-quality isolate genomes that originated from different human participants (Supplementary Table 5). These isolates spanned 995 SGBs, of which 176 contained more than 5 genomes. As one SGB, SGB10068, assigned as *Escherichia coli*, contained 1,053 genomes and was larger than any other SGB, we randomly subsampled this SGB to 25% of its original size so that it became comparable in size to the second-largest SGB. This SGB was renamed as SGB10068s to indicate the subsampling process. Each SGB was assigned to its corresponding family, genus and species-level taxonomy in the MetaPhlAn4 database.

### Estimation of recombined and clonal genome fractions via mixture modelling

To estimate the recombined and clonal fractions in a pair of genomes, we developed a method that uses a combination of maximum-likelihood estimations (MLEs) and hidden Markov models (HMMs). This method is conceptually similar to a previously published method^22^, but with several technical adjustments (detailed at the end of the model validation section below). The major rationale behind the method is that SNPs introduced by mutations between a pair of genomes should be randomly distributed across the genomes, whereas recombination generates regions in the genomes that have an increased or decreased number of SNPs depending on whether the recombined genome fragment stems from a distant or close relative (Extended Data Fig. 1). It is therefore possible to partition genome alignments into regions that have been vertically inherited or recombined based on SNP distributions across the alignment.

The SNP distribution for each pair of strains was determined by sliding 500 bp windows across pairwise genome alignments with a step size of 50, which resulted in a probability mass function *P*(*x* = *n*) of 500 bp windows that have *n* SNPs. This probability mass function was modelled as a fractional sum of a Poisson distribution that represents the clonal fraction of the genome and as a negative binomial distribution that represents the recombined fraction of the genome using the following equation:

$$P(x=n)={f}_{c}\frac{({{\mu }_{c})}^{n}}{n!}{e}^{-{\mu }_{c}}+{(1-f}_{c})\frac{\varGamma (n+\alpha )\,}{\varGamma (\alpha )\,n!}{\left(\frac{\alpha }{\alpha +{\mu }_{{nc}}}\right)}^{\alpha }{\left(\frac{{\mu }_{{nc}}}{\alpha +{\mu }_{{nc}}}\right)}^{n}$$

where *µ*_*c*_ and *µ*_*nc*_ are the means for the SNPs per window in the clonal fraction (Poisson distribution) and the recombined fraction (negative binomial distribution), respectively, and *α* is the dispersion parameter of the negative binomial distribution. The fraction of the genome that is clonally inherited or recombined is represented by *f*_*c*_ and 1 – *f*_*c*_, respectively. The observed SNP distribution was fitted to the equation using MLE with the L-BFGS-B algorithm in the Python package SciPy^57^. As the negative binomial distribution can be interpreted as a gamma mixture of Poisson distributions with the dispersion parameter *α*, and approaches the Poisson distribution when *α* = 1, the lower bound of *α* was set as 2 so that the distribution was sufficiently distinct from the Poisson distribution that represents the clonal fraction. The effective recombination rate *r/m*, which is the number of SNPs exchanged by recombination relative to the number of SNPs introduced by mutation, can be calculated as ${\mu }_{{nc}}({1-f}_{c})$/${\mu }_{c}{f}_{c}$.

The estimated recombination and clonal fractions were further validated through the use of a HMM with the Python package pomegranate^58^, in which the two fractions serve as hidden states (*C*, clonal state; *R*, recombined state). The Viterbi training algorithm was applied to the spatial SNP profiles for each pairwise alignment, with *f*_*c*_ and 1 – *f*_*c*_ from the MLE as the starting proportion of *C*:*R*. Similarly, the initial parameters for the HMM emission matrix were generated from the MLE estimated Poisson and negative binomial distributions. Although in most cases, the HMM produced results that were highly consistent with the MLE, the HMM validation step was effective in correcting occasional MLE failures at low clonal divergences. Furthermore, the relative occurrence rates of recombination to mutation (*ρ/θ*) could be estimated as the total number of *R* states divided by the total number of SNPs in the *C* state.

### Validation of model performance with simulated data

To assess the performance of our mixture model across the expected biological ranges of evolutionary processes in gut bacteria, we evaluated it on sets of simulated genomes that covered a wide range of population genomic parameters. This approach also enabled us to optimize our model so that it would be most effective in the divergence range for which recombination is expected to influence the detection of GWSSs.

We generated sets of genomes (*n* = 64) with the program CoreSimul^59^, a forward simulation program that evolves a single genome along a phylogenetic tree to generate derived genomes while incorporating recombination. For each phylogenetic tree, 144 parameter combinations were tested: (1) the scale (that is, maximum pairwise distance) of the tree *s* = 0.0002, 0.001, 0.005, 0.02, 0.036 and 0.05; (2) the size of the recombination fragment, exponential distributions with mean *δ* = 200, 500 and 1,000; (3) the relative occurrence rates of recombination to mutation *ρ/θ* = 0.01, 0.1, 0.2 and 1; and (4) the rate of exponential decay with divergence for success of recombination *Φ *= 9,18, when *P*_success_ = 10^−*πΦ*^. We simulated the evolutionary process of a 2 million base-pair genome diverging into 64-genome populations with 2 different types of phylogenetic structures: one in which multiple genome-wide sweeps have occurred (Extended Data Fig. 2a) and another one in which the tree is fully balanced (Extended Data Fig. 2g). During each time segment (that is, time between consecutive nodes) on the tree, each branch on the tree receives mutations (Jukes–Cantor 69 substitution model) and recombination events based on a Poisson process, but only branches that overlap in time are allowed to recombine with each other, and the probability of successful recombination exponentially decreases with sequence divergence^60^. For each pair of genomes, we tracked all regions that have undergone recombination since their last recent common ancestor, and regions with overlapping recombination events were merged and treated as a single event. Finally, we applied our mixture model to the simulated genomes and conducted a comparison between the estimated recombined fraction of the genome, the clonal divergence and two measures of recombination (relative effect of recombination and mutation *r/m*, and relative recombination to mutation occurrence rate *ρ/θ*) with their corresponding values in the simulation.

We found that our method performed well when there were >1,500 total SNPs (>0.075% divergence, including SNPs introduced by both recombination and mutation) in the pairwise alignment, when the majority of recombination fragments were >500 bp and when the overall recombined fraction of the genome was more than two-thirds of the genome. We propose several reasons for these limitations. First, the method becomes noisy when the overall number of SNPs falls under 1,500, which is probably due to a lack of sufficient SNP-containing windows for either the MLE or the HMM to perform efficiently. Second, our method considerably overestimates the recombined fraction of genomes when the length distribution has a mean of 200 because too many windows (500 bp) are only partially recombined, and the fraction of windows that are identified as recombined no longer equals the fraction of the genome that is simulated as recombined. Third, the method also loses accuracy when the mean divergence of the recombined fraction is less than 2.5 times that of the clonal fraction as the SNP distributions in these two fractions overlap too much to be sufficiently resolved from each other. This mostly occurs when more than two-thirds of the genome is recombined under our parameter settings.

Considering the above results, we optimized our method so that it would perform best in a range characterized by low-to-intermediate levels of genome recombination as expected in GWSS clusters that are of relatively recent origin and hence still retain a high fraction of vertically inherited genome (clonal frame). Our optimization strategy involved using an intermediate window size for counting SNPs and filtering at both ends of the recombination spectrum where either a very small or very large fraction of the genome was recombined. We opted for a window size of 500 bp because the recombination fragment size in bacteria is estimated to range from tens to thousands of base pairs^22,61,62^. Moreover, further using a smaller window size could compromise the resolution of the method at low divergences owing to insufficient numbers of SNPs per window. Validation of our method using simulated data enabled us to establish robust filters to ensure accurate parameter estimation, free from the influence of degenerate parameter sets resulting from the MLE being confined to a local minimum. These filters were set for genome pairs that were expected to be very highly or lowly recombined. All genome pairs with less than 1,500 SNPs were considered 100% clonal, with the divergence of the clonal fraction deemed as 10^−5^. Meanwhile, all genome pairs for which the estimated mean of the recombined fraction is less than 2.5 times that of the clonal fraction were considered as 100% recombined, with both the clonal and recombined fractions of the genome sharing the same divergence as the overall genome alignment.

As a precaution against sporadic failures of the MLE, we also implemented a corrective measure. We cross-checked whether the MLE-estimated recombination fraction exceeded twice that determined by the HMM. If such a discrepancy occurred, we substituted the MLE-estimated parameters with those derived from the HMM. Conversely, if no such discrepancy was observed, the MLE-derived clonal divergence and recombination fractions were deemed the final estimated parameters. As the HMM was also used to determine the spatial information of the recombined regions, we found that most recombined regions that stretched for less than eight consecutive sliding windows in the HMM were falsely identified. Therefore, we reassigned them as clonal regions after completion of the HMM.

### Identifying putative GWSSs from the isolate collection

We established two criteria for the conservative identification of putative GWSSs in the isolate genome collection and encapsulated the relevant workflow into a package called PopCoGenomeS^24^. First, to ensure a sufficiently large clonal frame for confident phylogenetic analysis and downstream metagenomic mapping, we only considered genomes that were predominantly vertically inherited (that is, the pairwise recombined portion is <50%). Second, the divergence among the genomes considered should satisfy the 5× rule, which is a stricter variant of the previously established 4× rule^25^. According to the 4× rule, if sister clades on a tree with the same sample size are separated by a distance gap that exceeds 4 times the within-clade distance, there is less than 5% probability that the clades are formed due to random drift. The 5× rule decreases the probability of drift to less than 1% and allows for uneven sample sizes, including cases when the sister clade is represented by a single genome^26^.

To first identify groups of isolates with mostly vertically inherited genomes (clonal frame >50%), we applied our mixture model and its associated filters to each of the 176 SGBs that contained more than 5 genomes. In each SGB, we identified vertically inherited groups of genomes using the package micropan (bClust, average linkage)^63^ in R to generate networks of genomes for which pairwise vertical inheritance averaged >50%, as determined by our mixture model. In some SGBs, the fraction of recombined genomes plateaued or gradually decreased with clonal divergence after the initial increase, which may be due to the model nearing the limits of its suitable range (Supplementary Fig. 1). Therefore, from each vertically inherited genome cluster identified, we removed genomes for which the average divergence from other cluster members exceeded that of genomes outside the cluster.

We then checked whether entire groups of vertically inherited genomes could be putative GWSS clusters. We applied the 5× rule to each vertically inherited group of genomes in a SGB by determining whether the most closely related isolate outside the group was more than 5× distant compared with the average clonal divergence in the group. If this condition was met, the entire genome group was considered a putative GWSS cluster. Subsequently, we scanned all vertically inherited groups of genomes for evidence of GWSSs in the group. Each clade in a maximum-likelihood tree, constructed based on whole genome alignments of a vertically inherited group (phyml, GTR + G + I model)^64^, was evaluated according to the 5× rule. If the average clonal divergence in a clade was less than one-fifth of that between it and its sister clade, then the clade was identified as a GWSS cluster.

### Validation of putative GWSS clusters in metagenomes

We sought to validate the structure of GWSS clusters and the extent of their occurrence in metagenomes representing a large diversity of host conditions and geographical locations. To confirm that the 5× distance gaps for putative isolate GWSS clusters were not due to incomplete or biased sampling, we developed a pipeline that enabled testing of the 5× rule by combining isolate genomes and metagenomes. To this end, we identified a consensus clonal frame (CCF) for each putative GWSS cluster based on the isolate genomes and then implemented the 5× rule twice using two distinct distances: first, the distance of each genome and metagenome sample to the CCF; and second, the pairwise distances between all isolate genomes and metagenome samples based on their alignments to the CCF. This procedure is described in detail below.

First, we constructed a database in which each isolate-based GWSS cluster was represented by its clonal frame to ensure that the distances we calculated between metagenomes and each putative GWSS cluster reflected only vertically acquired substitutions. We determined whether the clusters are nested (if one cluster completely encompasses another), and only kept the encompassing cluster. For putative GWSS clusters that consisted of three or more genomes, we extracted the clonal frame of each cluster by removing all recombined fragments in the core genome alignment (Mugsy (v.1.2.3)^65^) of the sweep with ClonalFrameML (v.1.12)^66^ and constructed a CCF by selecting the major allele of each SNP. For GWSSs with only two genomes, we extracted the clonal frame by removing all recombined segments (+500 bp upstream and downstream) identified by our previous bipartitioning HMM model and randomly assigned the clonal frame of one genome as the CCF. We then clustered all of the CCFs with fastANI (v.1.33)^53^ and sorted CCFs with ANI > 99% into separate databases. This resulted in 6 CCF databases each containing 53–75 clonal frames.

Second, to ensure that the addition of metagenomic samples to the GWSS clusters successfully mitigated potential isolate sampling bias, we acquired a subset of metagenomes that covered many host phenotypes from the curatedMetagenomicData (v.3.4.2)^67^ database. Stool metagenomes were first dereplicated by human participants so that for samples sequenced over a time series from the same participant, only the sample with the maximum number of reads was kept. We then grouped the samples by study, age category, disease and country, and selected up to five metagenomes from each unique group combination. If multiple participants from the same family were included, we only kept the metagenome for one adult member. This process resulted in a collection of 1,477 metagenomes representative of 74 datasets (Supplementary Table 6).

Third, to ensure that calculation of sequence distances between the metagenomes and the CCF do not represent a mixture of strains, we filtered for metagenomes that were dominated by a single strain from each sweep-containing SGB. Metagenomes were aligned against the MetaPhlAn4 reference database (v.Jan 2022) with MetaPhlAn (v.4.0.6)^23^ default settings, and all polymorphic sites with a Phred quality score ≥20 and coverage ≥3 were identified. The allele frequency spectrum was generated for each SGB with >40 polymorphic sites in every metagenome, and SGBs for which the 5th percentile of the spectrum exceeded 0.8 was considered as single-strain dominance in that metagenome. This cutoff roughly corresponds to a ratio of 9:1 between major and minor strains and reduces the number of usable metagenome samples per SGB to between 1 and 742 (Supplementary Table 7).

Fourth, we established a consistent metric to determine the distances of isolate genomes and metagenomes to the CCFs. To determine the distances between single-strain metagenomes and the CCFs, we aligned metagenomes to each of the 6 CCF databases with bowtie (v.2.5.1; -X 2000, --no-mixed, --very-sensitive)^68^ and calculated the distances as 1– the popANI metric in inStrain (v.1.7.5; default settings)^69^. The popANI metric takes into account both major and minor alleles when calculating the distance between metagenomic samples aligned to a reference, which makes it a population-level measurement of ANI. Furthermore, to enable direct comparisons between the distances of isolate genomes and metagenomes to CCFs, we converted isolate genomes to synthetic fastq reads (HiSeq 2500 platform, 10× coverage, ART-2016.06.05)^55^ and calculated their distances to the CCFs in the same manner as the metagenomes. We included all isolates that were in the putative sweep clusters as well as up to six isolates that were most closely related to the sweep (‘sister isolates’) in the calculation. Eventually, only distances calculated from samples with 2× coverage and 25% coverage breadth (50% for isolate genomes originating from the sweep) were retained for each clonal frame.

Finally, we applied the 5× rule to the sample-to-CCF distances for each GWSS cluster putatively identified on the basis of isolate genomes. All samples, processed as described above, were sorted by their distance to the CCF from the nearest to the furthest. Then, beginning with the third sample in proximity to the CCF, we progressively examined samples in increasing order of distance until a sample for which the distance from the CCF exceeded five times the average distance of all samples closer to the CCF was identified. Therefore, a GWSS consisted of least three samples. All samples in closer proximity to the CCF than the identified sample were deemed part of the sweep. To avoid identifying GWSSs based on outlier samples that aligned to the corresponding CCF (that is, genomes or metagenomes misassigned to a certain SGB), we eliminated all sweeps with fewer than three samples found outside the sweep. Moreover, as some SGBs are known to be mixtures of recently diverged species, and distance gaps can arise from samples far away from the reference CCF not surpassing the coverage threshold, we excluded sweeps if the number of samples in the sweep exceeded two-thirds of the total number of samples and if, simultaneously, the distance of the closest sample outside of the sweep was more than 1.5 times further from the CCF compared with that determined only by isolates. Finally, in cases when a sample was assigned to two sweeps from the same SGB, we removed the sample from the sweep with less coverage and reran the sweep assignment for the corresponding CCF.

As a final verification of the GWSSs identified by sample-to-CCF distances, we performed an additional 5× test based on pairwise distances. This test included all samples (isolate genomes and metagenomes) in the GWSS cluster as well as up to six of the most closely related isolate genomes and metagenomes to the GWSS cluster. For GWSSs occurring in more than 200 samples, we subsampled across the range of distances to the corresponding CCF to obtain a final set of approximately 100 samples for analysis. Pairwise genetic distances between samples (isolate genomes and metagenomes) were calculated by obtaining the 1 – popANI between samples when mapping to the same CCF using inStrain (v.1.7.5)^69^. We only considered pairwise samples for which more than 25% of the reference CCF was covered by both samples. We then assessed whether the average pairwise distance for all samples in the GWSS cluster was less than one-fifth of the average minimal distance of each within-GWSS sample from its closest relative outside the GWSS cluster. GWSSs that passed this additional 5× test were confirmed as true GWSS clusters.

The phylogenetic structure of all confirmed GWSSs was determined by extracting the bac120 marker gene set in the GTDB-Tk database (R214)^44^ from their corresponding CCFs and inferring a phylogenetic tree from the marker gene alignments using default settings in GTDB-Tk (v.2.3.2)^44^.

### Robustness of GWSS assignments

We evaluated the sensitivity of GWSS identification to the adjustments of key parameters used to detect them. We focused on three types of parameters: (1) how the recombination fraction cutoff used to determine vertical inheritance affects the detection of isolate-based sweeps; (2) how the number of metagenomes included influences the number of GWSSs detected; and (3) how changes to coverage cutoffs, both in depth and breadth, for samples mapping to CCFs affect GWSS detection. We tested 54 combinations of recombination and coverage cutoffs (parameter types 1 and 3) spanning all stages of the GWSS pipeline and found that the number of total and young (<100 years old) GWSSs identified changed by less than 15% (Supplementary Table 8 and Supplementary Fig. 3c), with the dominant effect arising from the recombination fraction cutoff (Supplementary Fig. 3a). For parameter type 2, rarefaction analysis showed that although the number of GWSSs detected increases with the number of metagenomes included, this trend plateaued between 20 and 40% of the total dataset (Supplementary Fig. 3b), which suggests that the number of metagenomes used in the current analysis is sufficient to recover nearly all GWSSs detectable with the available isolate genomes. Together, these findings indicate that the GWSS assignments are robust to parameter adjustments and remain stable across reasonable changes to cutoff values and sampling effort. Further details on the parameter adjustments and their effects are provided in Supplementary Table 8.

### Sensitivity of GWSS assignments to low-level occurrence of closely related strains

We assessed the sensitivity of GWSS assignments to the low-level co-occurrence of closely related strains. Using the largest of our 6 CCF databases (comprising 75 CCFs), we introduced simulated reads, derived from the SGBs containing the CCFs at 0.5× coverage of an isolate randomly sampled in the SGB, into all 1,477 metagenomes used for sweep confirmation. We found that the introduction of these simulated reads did not alter the number or identity of the GWSSs detected. Examination of two key parameters, the maximum divergence in the GWSS (Supplementary Fig. 4a) and the distance of the GWSS to the closest relative (Supplementary Fig. 4b), indicated that these distances also changed minimally. Overall, this analysis suggests that the existence of closely related strains at a low level has a limited impact on GWSS detection and does not substantially affect the robustness of our approach.

### SGB category assignments

All 176 SGBs for which we performed GWSS searches were classified into commensals, pathogens or commensal SGBs that are frequently found in fermented and functional foods (probiotics) according to the following standards. An SGB was categorized as a pathogen if we found that at least a portion of isolate genomes originated from an outbreak by checking the source studies of the isolates. The criterion was that multiple (≥3) genomes were sequenced from the outbreak, as this is also the lowest number of genomes required for the identification of a GWSS. An SGB was designated as a probiotic if literature searches of the corresponding taxa revealed a species commonly found in probiotic products or fermented foods. The remaining SGBs were classified as commensals. The classification of SGBs is provided in Supplementary Table 1.

### Calculation of Tajima’s *D* for GWSS clusters

For calculation of the Tajima’s *D* of a GWSS cluster, a clonal frame was reconstructed for each isolate and metagenome sample associated with the GWSS based on the SNP profile of the sample when mapped against the CCF of the GWSS cluster. All reconstructed clonal frames in a GWSS cluster were used to calculate the Tajima’s *D* of the cluster and its significance level, assuming that *D* follows a beta distribution using the tajima.test function in the pegas package (v.1.3)^70^ in R.

### GWSS cluster age estimation

The age of each GWSS cluster was calculated using two independent methods: (1) dividing the maximum pairwise SNP distances in each cluster by 2 and then with a constant molecular clock of 1–10 mutations per genome per year; and (2) estimating a molecular clock from strains in twin metagenomes or metagenomic time series. In each GWSS cluster, the SNP distances between strains in two samples were calculated by normalizing the population_SNPs metric (inStrain (v.1.7.5)^69^, defined as sites for which coverage is >5× with no shared alleles between the samples) by the fraction of reference CCF with >5× coverage in both samples. This pairwise SNP distance calculation was performed exclusively on samples for which more than 25% of the reference CCF had >5× coverage.

We were able to estimate the metagenomic molecular clock of nine SGBs from the metagenome time series or twin metagenomes by finding all strains that persisted in individuals over a period of time or were shared between twins. We retrieved all metagenomes from the same human participant who was at least sampled 1 year apart from curatedMetagenomicData (v.3.4.2)^67^. If multiple time points were sampled for the same human participant, we selected the two time points that were furthest apart. We also retrieved all metagenomes and their related metadata from 250 adult twins from the TwinsUK study^71^. Twins were assumed to have identical strains when they were living in the same household, and the years that the twins had lived apart were assumed as the time that the strains had to accumulate mutations. The genetic distance between strains in each metagenome and the CCF of their corresponding GWSS were calculated in the same manner as in the previous section (‘Validation of putative GWSS clusters in metagenomes’). To account for shifts in strain dominance and strain replacement events over time, we only considered metagenomes from the same person or twin pair to be sharing strains from the same GWSS if one metagenome was more closely related to the reference CCF of the GWSS compared with the threshold previously used to establish the GWSS cluster, whereas the other metagenome was closer to the CCF than half of the minimum distance observed for metagenomes outside of the GWSS cluster.

For each SGB, the metagenomic molecular clock was expressed as a linear function, with the SNP distance between shared strains in metagenomes as the independent variable and the time difference between the metagenomes as the dependent variable. When SGBs contained shared strains in both the metagenome time series and twin metagenome datasets, the linear function was determined as the best fit across all data points. For SGBs with shared strains in only one dataset, the function was defined as the average slope of lines constrained to pass through each data point and the origin. We eventually estimated the age of every GWSS cluster belonging to the nine SGBs by extrapolating the corresponding metagenomic molecular clock to the maximum pairwise SNP distance of the GWSS cluster.

### Validation of GWSS detection and age with pathogen datasets

We evaluated how well our sweep detection and age estimation pipeline performed on pathogens with well-documented pandemics, as many of these can be considered as rapid, global genome-wide sweeps. We selected *Vibrio cholerae* as a validation case because its ongoing seventh pandemic, which includes all currently circulating pandemic strains, originated from a single source population in the Bay of Bengal followed by local diversification^72^. These seventh pandemic strains form a distinct clonal group known as the L2 phyletic lineage. Given these features, all modern *V. cholerae* L2 isolates (that is, collected after 1995) should be identifiable as a GWSS. We briefly summarize the evaluation results here, with the full details provided as Supplementary Text.

**Extended Data Fig. 9:**
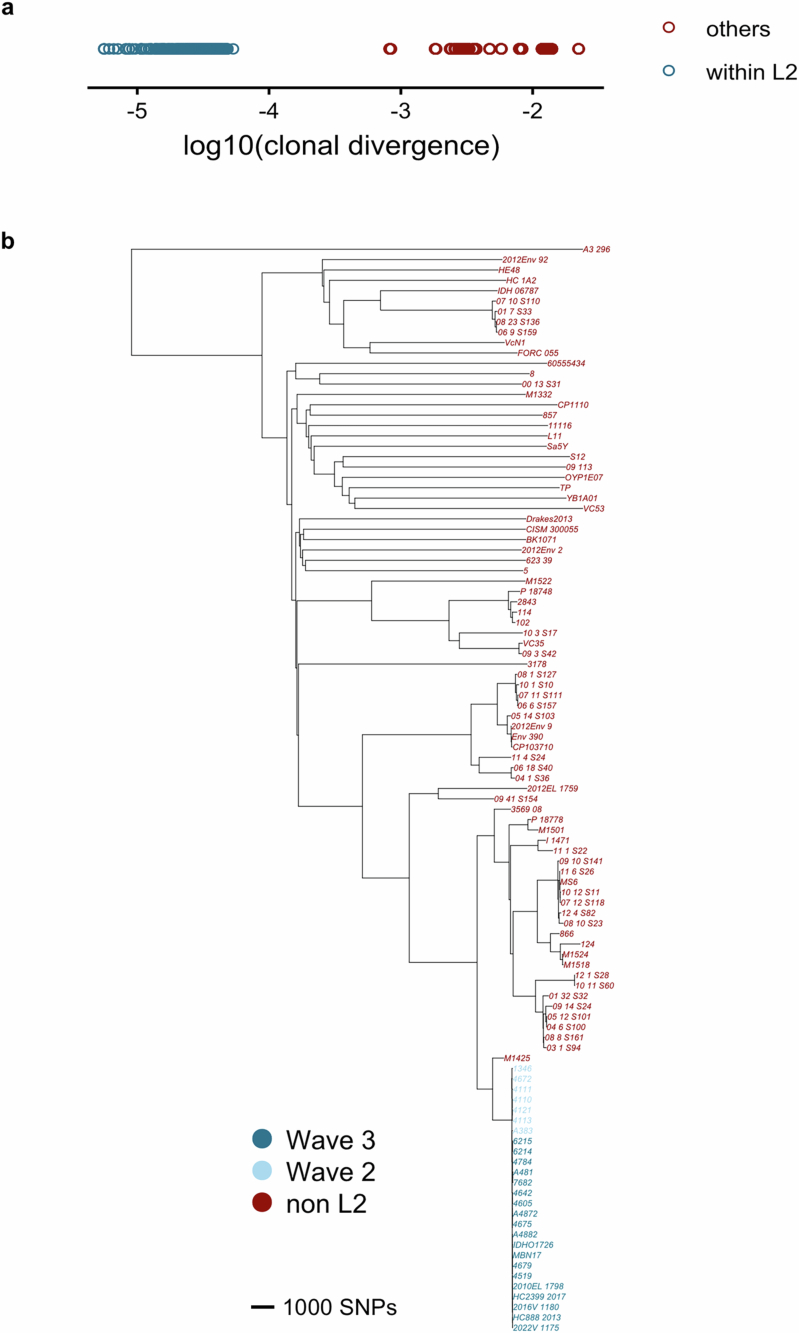
Evaluating the sweep detection and age estimation pipeline with *V. cholerae* strains from the 7^th^ pandemic. **a**. The L2 lineage identified as a distinct clonal GWSS cluster with a clear divergence gap from non-L2 isolates. Each dot represents a mock metagenome generated from a *V. cholerae* isolate genome. **b**. Neighbor-joining tree constructed from pairwise distances among mock metagenomes derived from isolates within the L2 lineage and from non-L2 lineages.

Using dereplicated datasets of modern L2 isolates (post-1995) and non-L2 controls (Supplementary Table 9), we showed that when these isolate genomes are converted into simulated human gut metagenomes with *V. cholerae* infection, our method accurately identified the L2 lineage as a distinct clonal GWSS cluster with a clear divergence gap from non-L2 isolates (Extended Data Fig. 9a). We also estimated sweep ages for the currently circulating strains and individual waves of the seventh pandemic using a molecular clock of one to ten SNPs per genome per year. Historically, the seventh cholera pandemic comprises three global waves, with the first wave now extinct and only strains from the second and third waves still circulating^72^. We found that the estimated sweep ages (5–51 years for the overall circulating pandemic strains and 4–46 years for the third wave) closely matched historical estimates of 45 and 35 years^72^, respectively, and the neighbour-joining tree based on the clonal divergence among isolates resolved waves 2 and 3 as discrete, nested clusters (Extended Data Fig. 9b).

We also used this dataset to test whether using the CCF as the representative of a sweep cluster affects sweep detection or age estimation. Specifically, we compared sweep detection and age estimation results using the CCF for the L2 lineage to those obtained using clonal frames derived from ten randomly sampled L2 isolates. The difference was minimal: the maximum pairwise distance in the entire L2 lineage (representing the seventh pandemic) calculated using the CCF was 103 SNPs, which corresponded to an estimated sweep age of 5.1–51.5 years. By comparison, the maximum pairwise distance calculated using 10 randomly sampled L2 isolates as references was 116 ± 4.6 SNPs, which corresponded to a sweep age of 5.8–58 years. Thus, any potential bias introduced by using the CCF is negligible, particularly given that our goal was to estimate the sweep age on the correct order of magnitude.

### Curve fitting for measuring recombination rates

Because in most SGBs the fraction of the genome that had undergone recombination increased linearly as the number of mutations in the clonal region increased, and subsequently plateaued, the slope of the linear segment of the recombined fraction–mutation plots is a measure of recombination rate. We therefore segmented all recombined fraction–mutation plots (Supplementary Fig. 2) for commensal bacteria to find their linearly increasing regions using the R package dpseg (v.0.1.1)^73^. Sometimes a large number of data points clustered at low divergence and this could lead to oversegmentation of the scatter plot. Therefore, we subsampled the plot to 100 data points when there were more than 100 data points with fewer than 2,000 mutations. A total of 4 parameter combinations that included a breakpoint penalty, a minimal segment length and a maximal segment length were tested for the curve fitting ((0.2,20,40), (0.1,10,20), (0.1,5,10), (0.2,20, all data points)). If the first linear fragment of the fit had *R*^2^ > 0.8, then this fragment was determined as the linearly increasing region. If *R*^2^ > 0.8 was satisfied under multiple parameter combinations, then the combination that had the maximum *R*^2^ or allowed all data points to fit to a single linear fragment with *R*^2^ > 0.8 was used. Otherwise, consecutive linear fragments with similar (within 75%) slopes were combined and refit as one fragment, and the first fragment with *R*^2^ > 0.33 was determined as the linearly increasing region. For SGBs in which automated segmentation was not satisfactory, we manually identified the linear range of increase. Finally, for all the identified linearly increasing regions of each SGB, we added a point (0,0) to the data points in the region and applied a linear regression model passing through the origin. The slope of the linear regression model was used as the recombination rate, and we were able to measure the recombination rates for 45 out 46 of the commensal SGBs with sweeps, and 52 out 95 of those without sweeps. The lower fraction of satisfactory fits in the SGBs with no confirmed sweeps was due to both fewer genomes per SGB and a more frequent absence of data points in the linearly increasing fraction of the recombined fraction–mutation plots. All curve fittings are shown in Supplementary Fig. 2, and all measured recombination rates are in Supplementary Table 10.

### GWSS identification from StrainPhlAn marker gene trees

Because we were interested in testing associations of GWSS clusters with human disease or physiological states, we explored the feasibility of identifying GWSSs based on phylogenetic distances of marker genes extracted from the metagenomes as this approach can be more easily scaled up to large metagenomic datasets. As our SGB classifications were based on the MetaPhlAn4 database, we performed strain-level marker gene profiling for SGBs in metagenomes with StrainPhlAn4 and tested how and to what extent the 5× rule could be extended to StrainPhlAn4 marker gene trees to identify GWSS clusters. We set up two criteria for calling a GWSS cluster from the marker gene tree: (1) the normalized average genetic distance in a marker gene based GWSS cluster needs to be smaller than a normalized cutoff based on previously identified GWSS clusters; and (2) the phylogenetic distance between the proposed GWSS clade and its sister clade exceeds five times the average distance in the GWSS clade.

To define the cutoff for the first criterion, we constructed mock metagenomes for all isolate genomes in GWSS clusters. Each mock metagenome for an isolate genome consisted of synthetic fastq reads for the target genome at 20× coverage (ART-2016.06.05, -ss HS25 -f 20)^55^, and a randomly selected isolate genome from every other GWSS-containing SGB at 1× coverage. Therefore, the total number of mock metagenomes for each SGB is the number of isolate genomes identified in GWSS clusters for that SGB. For each SGB, strain-profiling was performed for each mock metagenome with StrainPhlAn4 against the MetaPhlAn4 reference database (v.Jan 2022)^23^, which resulted in a tree that was built using marker genes from all the isolate-based mock metagenomes and marker genes extracted directly from all other isolate genomes in the SGB. The cutoff was set as SGB-specific normalized phylogenetic distance (nGD) thresholds that optimally separated isolate pairs in GWSS clusters from isolate pairs that had only one isolate genome in the GWSS cluster. nGDs were calculated as leaf-to-leaf branch lengths on the SGB marker gene tree normalized by their median. For SGBs with at least 50 pairs of isolates in the GWSS cluster, nGD cutoff thresholds were defined based on the value that would maximize the Youden’s index (R package cutpointr, v.1.2.0)^74^, unless the value exceeded the 5th percentile of the isolate pairs that had only one isolate genome in the GWSS cluster. For SGBs with fewer than 50 total within-GWSS isolate pairs, the nGD corresponding to the 3rd percentile of the isolate pairs with only one isolate genome in the GWSS cluster was used as the cutoff.

StrainPhlAn marker gene trees were constructed for each SGB with the same isolate and metagenome samples previously used to identify GWSSs. For 32 out 46 of the commensal bacteria, at least 2 out 3 of the samples in previously identified GWSS clusters were retained in GWSS clusters called from the StrainPhlAn marker gene trees (Extended Data Fig. 5a). Also, because GWSS clusters identified from marker gene trees are not necessarily the expansion of isolate based GWSS clusters but can be purely metagenome based, for the majority of the SGBs (33 out 46), GWSS clusters called from the StrainPhlAn marker gene trees included more samples than those included in the previously identified GWSS clusters (Extended Data Fig. 5b).

### Association studies of GWSS clusters in metagenomes

Associations between GWSS clusters and five human health metrics, advanced age (>65 years old), CRC, UC, CD and T2D, were examined for each SGB. These five metrics were chosen because they represented different types of disease and health states that are related to gut microbiome dysbiosis and the availability of sufficient samples across diverse biogeographies. To start, we assembled a baseline metagenome database comprising 12 datasets, which collectively included 2,084 samples from 1,446 human participants in the curatedMetagenomicData database (v.3.4.2)^67^. This database includes all adult samples from the 12 datasets and excludes individuals who were on antibiotics. Eventually, this baseline database included 654 healthy individuals and 792 individuals with various diseases: atherosclerotic cardiovascular disease (*n* = 187), CRC (*n *= 132), inflammatory bowel disease (IBD, *n* = 186), glucose metabolism-related diseases (*n* = 131), rheumatoid arthritis (*n* = 89) or adenoma (*n* = 67). We further expanded the database by incorporating five additional CRC datasets, six IBD datasets (including both UC and CD) and six additional T2D datasets, applying the same filtering criteria. This extended dataset of 6,783 samples from 4,614 individuals (including 646 patients with CRC, 749 patients with T2D, 467 patients with CD and 342 patients with UC) captures all large-scale studies available so far for these diseases (Supplementary Table 11).

The full 6,783 sample dataset was used to identify GWSSs. For all 46 commensal SGBs with previously confirmed GWSSs, strain-profiling was performed with StrainPhlAn4 against the MetaPhlAn4 reference database (v.Jan 2022)^23^. Markers for each SGB were extracted from all isolate genomes and all metagenomes with single-strain dominance for the SGB (see the section ‘Validation of putative GWSS clusters in metagenomes’). For each SGB, all extracted markers were aligned, filtered and constructed into a maximum-likelihood tree according to the default settings under the accurate mode of StrainPhlAn4. The two criteria for identifying GWSS clusters in StrainPhlAn marker gene trees were applied to each SGB. A total of 1,479 GWSS clusters were identified in 40 out of the 46 commensal SGBs examined.

As further preparation for the association analysis, we performed additional filtering and metadata curation for all the samples involved in each SGB marker tree. As age and disease information were often unavailable for isolate genomes, we removed all isolate genomes from the SGB trees. For samples originating from the same participant or from participants in the same family, we only kept one sample at random from each participant or family member for each SGB tree. For each target disease (CRC, UC, CD and T2D), we compared samples from affected individuals with samples from participants without the corresponding disease (control group). To prevent the control group from being dominated by samples from other disease cohorts, the association analysis for each disease was restricted to samples from the baseline dataset and the corresponding disease-specific expanded datasets. For age-related analyses, participants aged >65 years were classified as ‘advanced age’ and the remainder as ‘normal age’; associations were assessed between healthy individuals in these two age groups.

Associations between GWSS clusters in each SGB and age and disease were examined by building a general linear model with stepwise, forward variable selection and false-discovery rate correction (Benjamini–Hochberg procedure). We asked whether being in a certain sweep or not has a positive or negative impact on the sample being from patients with a disease or those of advanced age with the formula *Y* (diseased or advanced age) = *β*_1_*S*_1_ + *β*_2_*S*_2_ + …*β*_*n*_*S*_*n*_ + *μ*, where *S*_1_, *S*_2_, … *S*_*n*_ represent GWSS clusters detected in each SGB. The forward selection was performed with the R package SignifReg (v.4.3)^75^ under the criteria that a new predictor is added to the model if the addition of the predictor further minimizes the model *P *value, and every individual predictor remains significant at *P*_adj_ < 0.05 after correcting for multiple hypothesis testing with the Benjamini–Hochberg procedure. All selected sweeps were further tested for geographical biases to ask whether sweeps are dominated by samples from certain countries by performing a chi-squared test for the country distribution in each sweep.

Associations between SGBs and disease or age were performed in the same manner as for GWSS clusters in each SGB, with the formula *Y* (disease or advanced age) = *β*_1_SGB_1_ + *β*_2_SGB_2_ + …*β*_*n*_SGB_*n*_ + *μ*, where SGB_1_, SGB_2_, … SGB_*n*_ represent individual SGBs. Owing to the large number of metagenomes associated with each SGB, we did not test for geographical bias in each SGB.

### Identification of sweep-specific genes

To identify sweep-specific genes that were specific to each GWSS cluster (genes that are both highly differentiated from other GWSS clusters and missing from sister genomes), we first predicted all protein-coding genes in the CCF and isolate genomes (Prodigal v.2.6.3)^76^ from each of the isolate-based sweep clusters. The protein-coding genes in each CCF were then pairwise aligned at the protein and nucleotide level (BLAST v.2.15.0+)^77^, and proteins found in a single CCF were selected as sweep-specific genes. Specifically, the selected proteins had no homologue in the other CCFs after filtering for alignments with over 60% amino acid identity and alignment length. We then required that the genes encoding the selected proteins in each CCF are identical at the nucleotide level in isolate genomes in the corresponding sweep cluster and share less than 60% nucleotide identity and alignment length with sister genomes (up to six isolates that were most closely related to the sweep). Selected protein-coding genes were annotated using EggNOG (emapper v.2.1.12, database v.5.0.2)^78^ and Prokka (v.1.14.6)^79^. Finally, to test for COG categories or Pfam families enriched in the sweep-specific gene clusters, annotations of the sweep-specific genes were compared with those in the entire CCFs using a Fisher’s exact test with Bonferroni correction.

### Statistical analysis

Statistical analyses and graphical representations were performed in R (v.4.2.1)^80^ using base R statistical functions and ggplot2 (v.3.5.1)^81^, ggpubr (v.0.6.0)^82^, ggtree (v.3.4.4)^83^, ggtreeExtra (v.1.6.1)^84^ and ComplexHeatmap (v.2.12.1)^85^. Correction for multiple testing (Benjamini–Hochberg procedure) was applied when appropriate and significance was defined at *P*_adj_ < 0.05. All tests were two-sided, except for those assessing functional enrichment of genes specific to GWSS clusters. To access differences between two groups, Student’s *t*-test was performed on data that passed the Shapiro–Wilk normality test; otherwise, a Wilcoxon rank-sum test was performed. Correlations were assessed with Spearman’s tests. All geographical biases in the datasets were accessed either with a chi-squared test or a Fisher’s exact test.

### Ethical compliance

For the Austrian isolate collection, study approval was granted by the ethics committee of the Medical University of Vienna (EK-Nr: 1617/2014, 1910/2019). All study participants gave written informed consent before study inclusion. The study was conducted in accordance with the ethical principles of the Declaration of Helsinki. The analysis of the Global Microbiome Conservancy isolate dataset was conducted with authorized access to data from the database of Genotypes and Phenotypes (accession phs002235.v1.p1) under approval from the US National Human Genome Research Institute.

### Reporting summary

Further information on research design is available in the Nature Portfolio Reporting Summary linked to this article.

## Online content

Any methods, additional references, Nature Portfolio reporting summaries, source data, extended data, supplementary information, acknowledgements, peer review information; details of author contributions and competing interests; and statements of data and code availability are available at 10.1038/s41586-026-10476-w.

## Supplementary information

Supplementary Information Supplementary Figs. 1–4, Supplementary Text Sections 1 and 2 and Supplementary References. The Supplementary Text validates the sweep-calling and sweep-age estimation framework using *V. cholerae*, a pathogen with well-documented clonal expansions and a known molecular clock rate. The analysis demonstrates that our method can validate that the ongoing seventh cholera pandemic is a GWSS cluster and that the method can recover known pandemic transmission waves and estimate their ages.

Reporting Summary

Supplementary Tables Supplementary Tables 1–11.

Peer Review File

## Data Availability

All newly sequenced genomes have been uploaded to the NCBI database under the BioProject identifier PRJNA1101861. The study and sample accession numbers for all isolates and metagenomes used are provided in Supplementary Tables 5, 6 and 11. Metadata for isolates were collected from the UHGG catalogue (v.1.0)^45^ and from the original publications of culturomic studies not included in the catalogue. Metadata for metagenomes were collected from the curatedMetagenomicData database (v.3.4.2)^67^ and from the original publications of studies not included in the database.

## Code availability

The PopCoGenomeS source code is available at GitHub (https://github.com/cusoiv/PopCoGenomeS) and archived at Zenodo (10.5281/zenodo.19139639)^24^. All other code have been uploaded to GitHub (https://github.com/cusoiv/genome_sweeps) and archived at Zenodo (10.5281/zenodo.19174060)^86^.
