# Supplementary material for: Genome-wide sweeps create ecological units in the human gut microbiome
Source: Nature. 2026 May 6;655(8121):202–9. doi: 10.1038/s41586-026-10476-w (PMC13322978; doi:10.1038/s41586-026-10476-w)
Supplement: Supplementary file 1 — Supplementary Figs. 1–4, Supplementary Text Sections 1 and 2 and Supplementary References. The Supplementary Text validates the sweep-calling and sweep-age estimation framework using V. cholerae, a pathogen with well-documented clonal expansions and a known molecular clock rate. The analysis demonstrates that our method can validate that the ongoing seventh cholera pandemic is a GWSS cluster and that the method can recover known pandemic transmission waves and estimate their ages. [file 41586_2026_10476_MOESM1_ESM.pdf]

nature portfolio

https://doi.org/10.1038/s41586-026-10476-w

Supplementary information

Genome-wide sweeps create ecological units in the human gut microbiome

In the format provided by the authors and unedited

Supplementary Data Figure 1

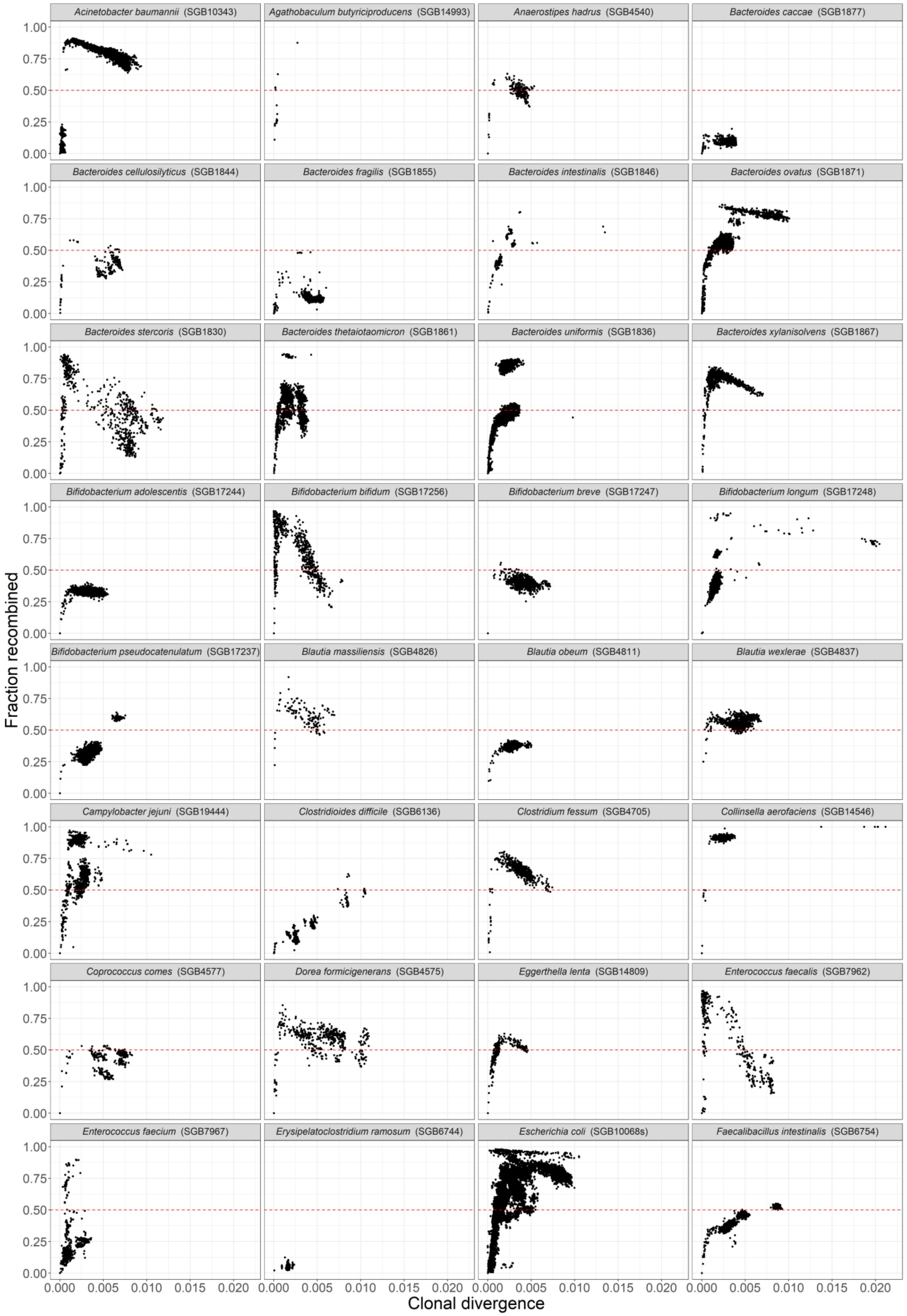

Supplementary Data Figure 1 (Continued)

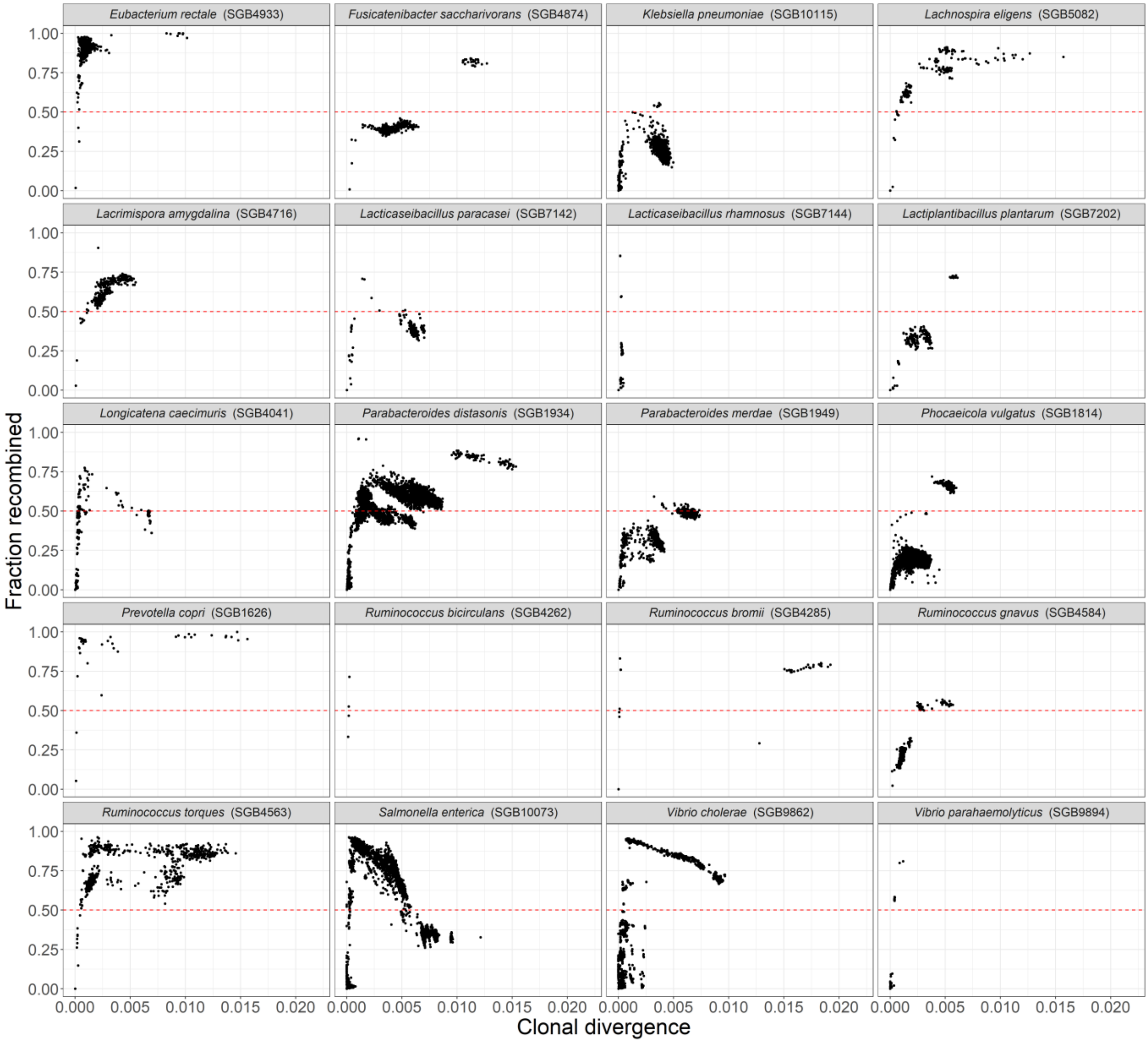

Supplementary Data Fig. 1. Recombination-divergence relationships among isolate genomes. The relationship between clonal divergence and fraction of the genome that has undergone recombination for all SGBs with more than 20 isolate genomes (52 out the total 176 SGBs), after implementation of filtering and corrective measures. Each dot represents a pair of isolate genomes and the red line at 0.5 is the cutoff chosen for predominantly vertical inheritance (< 50% recombined). Clusters of genomes below this threshold were subsequently utilized for downstream GWSS searches.

Supplementary Data Figure 2

a

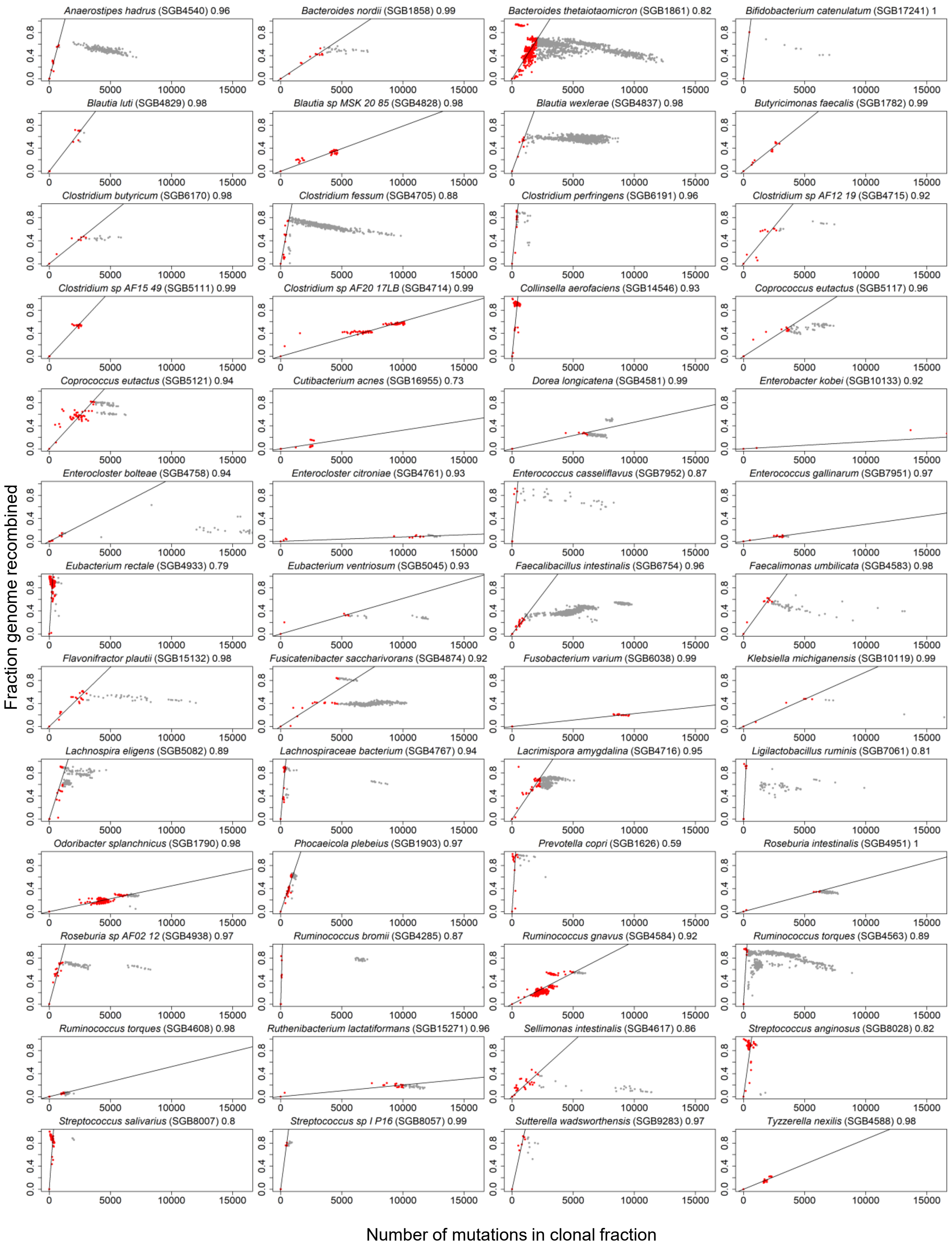

Supplementary Data Figure 2 (Continued)

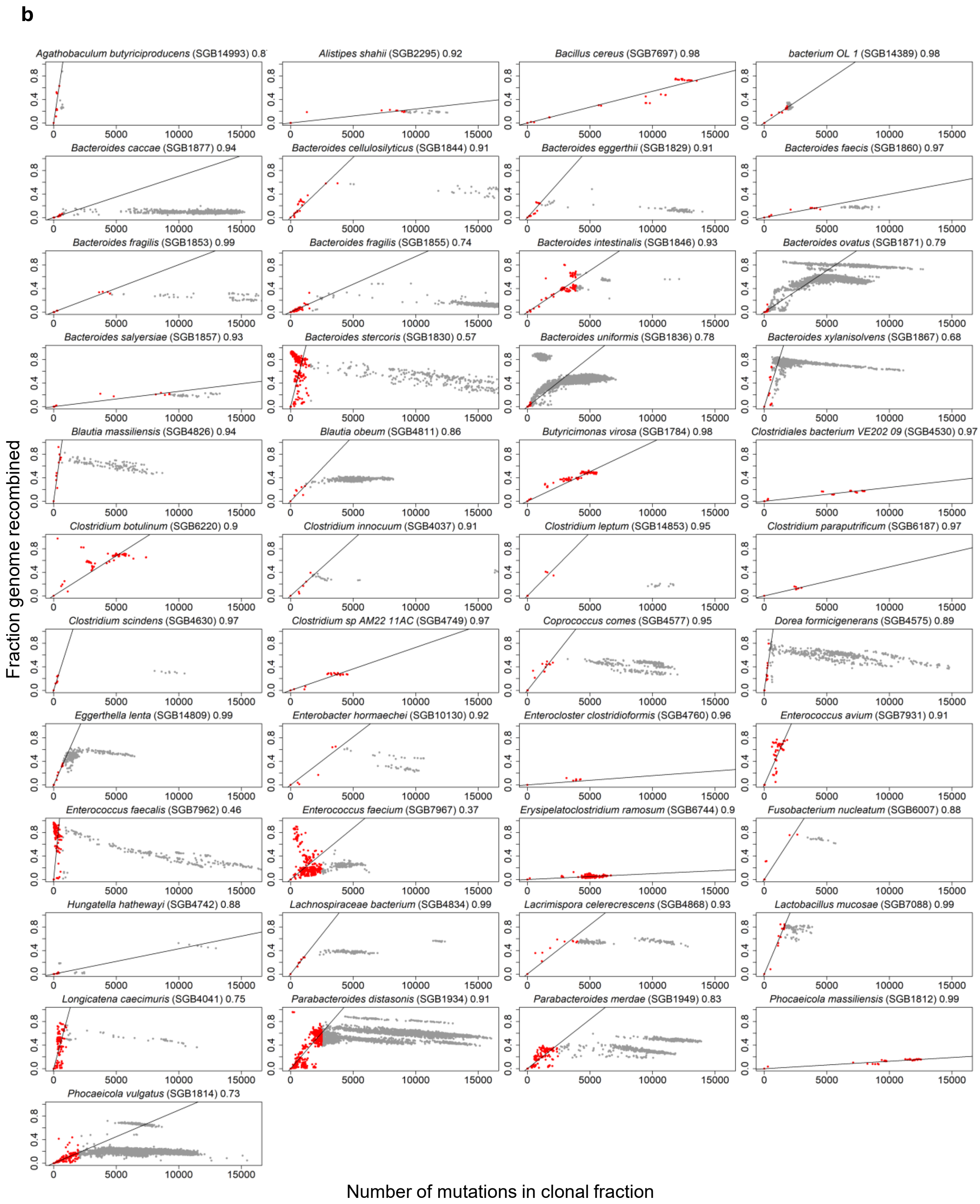

Supplementary Data Fig. 2. Recombination rates vary among SGBs. Curve fitting for all **a.** SGBs with no confirmed GWSSs and **b.** SGBs containing GWSSs with satisfactory fits ($R^2 > 0.33$) for the fraction of recombined genome vs. number of mutations in the clonal fraction. Each dot represents a pair of genomes compared, with red dots indicating those used for curve fitting, and the black line being the fitted line. The number following the alphanumerical SGB names in the title of each plot represents the R^2 of the fit.

Supplementary Data Figure 3

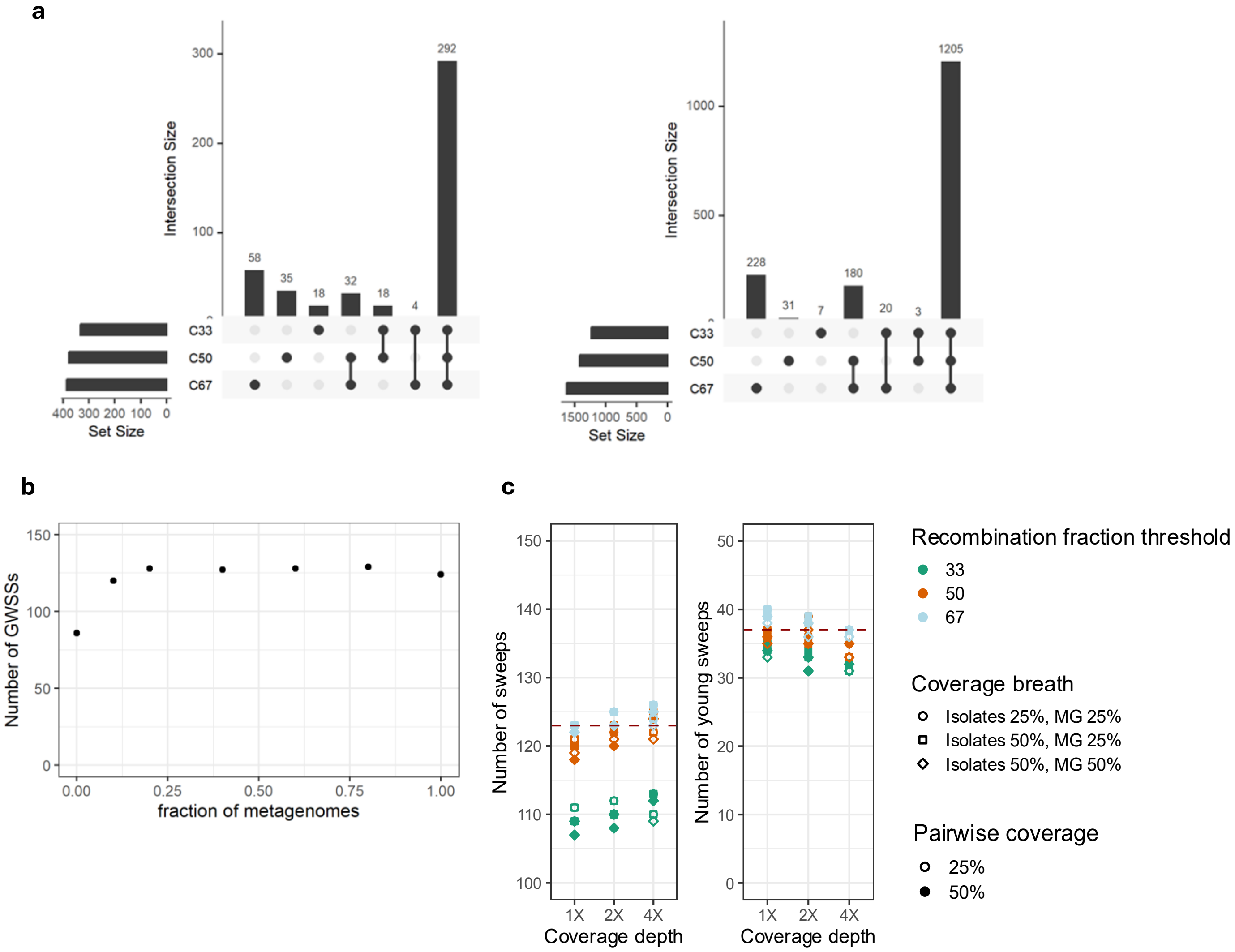

Supplementary Data Fig. 3. Robustness of GWSS assignments. a. Comparison of number of isolate-based sweeps (left) and the corresponding isolates within them (right) detected under different recombination cutoffs. In each UpSet plot, the horizontal bars on the left represent the total number of isolate-based sweeps (or isolate samples within sweeps) detected under three recombination fraction thresholds, respectively: C33 (< 33%), C50 (< 50%), and C67 (< 67%). The vertical bars represent the number of sweeps (or samples) shared across different thresholds, as indicated by the filled dots below each bar. **b. Rarefaction curve showing the number of GWSSs detected as a function of the fraction of metagenomes used.** The x-axis indicates the proportion of the 1,477 metagenomes included in the analysis, and the y-axis shows the total number of GWSSs identified at each subsampling level. **c. Comparison of the number of total (left) and young (< 100 years old, right) GWSSs identified across 54 parameter combinations spanning all steps of the GWSS pipeline.** Each dot represents the total number of GWSSs detected under one set of parameters, with the dot color representing the pairwise recombination threshold for the identification of the initial isolate-based sweep (33, 50, 67%). The x-axis shows coverage depth (1X, 2X, 4X) used for calculating the distance between the sample and the CCF of the isolate-based sweeps: these numbers set different minimum mean coverages required for a (isolate or metagenome) sample to be included in the calculation. Simultaneously, coverage breadth was varied with three settings for isolates and metagenomes (MG): Isolates 25%, MG 25%, Isolates 50%, MG 25%, or Isolates 50%, MG 50%. Finally, "Pairwise coverage" is the minimum shared covered fraction between two samples mapping to the same CCF when computing pairwise distances, set to > 25% or > 50%. The red horizontal lines represent the number of GWSSs identified with the parameters used in the paper.

Supplementary Data Figure 4

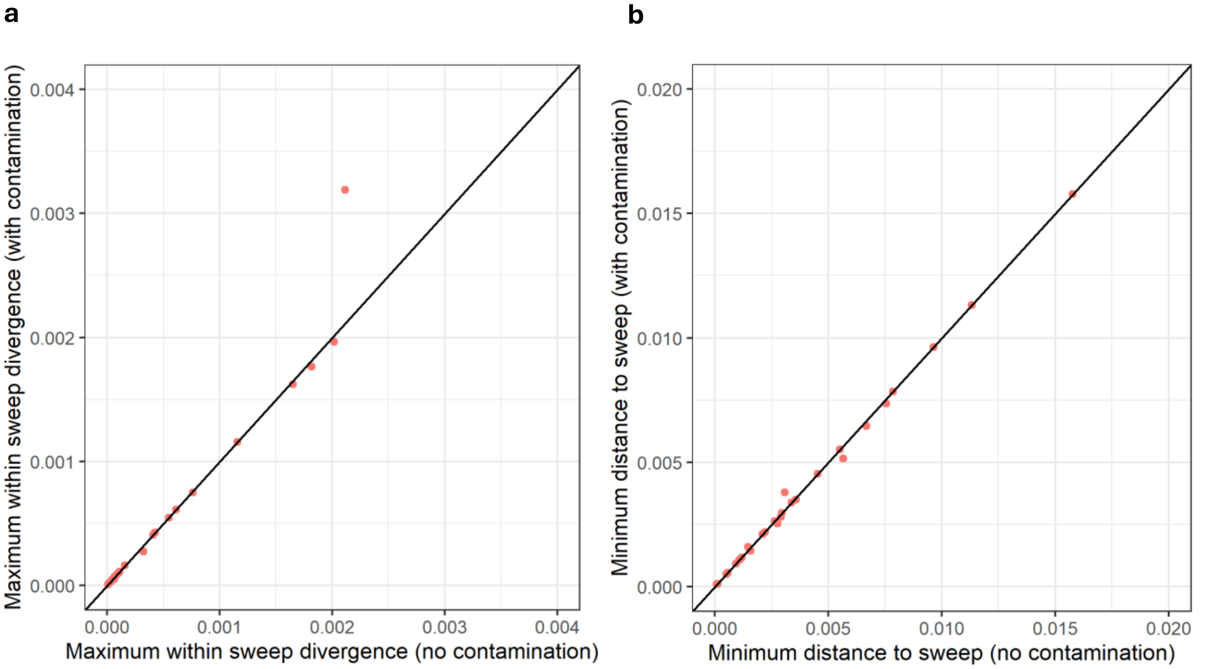

Supplementary Data Fig. 4. Effects of low-level occurrence of closely related strains on GWSS detection and a. maximum divergence within GWSSs and **b. distance of the GWSS to the closest relative.** Each dot represents a GWSS identified after the introduction of the alternative strain.

Supplementary Text

We selected *Vibrio cholerae* for the validation of sweep calling and sweep age calculation because it is a pathogen that colonizes the human gut with well-documented clonal expansions and global waves of transmission events, as well as an estimated molecular clock of approximately 3.3 SNPs per genome per year based on historical isolates ($8.3*10^{-7}$ SNPs $site^{-1}$ $year^{-1}$)[1]. Our analysis consists of two major parts:

1. Validation of the 7th pandemic as a GWSS. The ongoing 7th pandemic of *V. cholerae*, comprising all currently circulating pandemic strains, has been shown in previous studies to originate from a single source population in the Bay of Bengal, followed by local diversification and eventual extinction outside endemic regions. These 7th pandemic strains form a distinct clonal group, known as the L2 phyletic lineage. Given these features, all modern *V. cholerae* L2 isolates (i.e. collected after 1995) should be identifiable as a GWSS.

To validate that our method is capable of detecting *V. cholerae* from the 7th pandemic as a GWSS, we constructed a dataset of L2 isolates collected after 1995 and dereplicated them based on collection location, year, and pandemic wave (as it is known that the 7th pandemic consists of three distinct global transmission waves) to avoid oversampling of individual outbreaks[1]. In parallel, we assembled a dataset of non-L2 *V. cholerae* clinical isolates collected after 1995, similarly dereplicated by location, year, and lineage[2]. The 1995 cutoff was chosen because many historical isolates (and their associated lineages) are likely extinct, and their absence is part of what enables genome-wide sweeps to be detected. Including such lineages would confound GWSS identification. All strains that are used are detailed in Table S9.

Finally, due to the scarcity of metagenomes from cholera patients, and in order to mimic the isolate–metagenome framework used to identify GWSSs in our study, we simulated mock metagenomes by converting each *V. cholerae* isolate genome into FASTQ format (at 10X coverage of the original genome, ART-2016.06.05, -ss HS25, ref. [3]) and combining it with a randomly selected gut metagenome from a human individual without cholera. We then mapped these mock metagenomes to a consensus clonal frame constructed from all L2 isolates and calculated pairwise distances between the mock metagenomes using the same method and parameters as described in the section "Validation of putative GWSS clusters in metagenomes". We found that the L2 isolates from the 7th pandemic formed a distinct clonal cluster, with a clear divergence gap separating them from non-L2 isolates (**Extended Data Fig. 9a**). These results demonstrate that our method is capable of recovering known, pathogenic genome-wide selective sweeps.

2.Validation of different waves of the 7th pandemic and their ages. The 7th cholera pandemic is characterized by three successive global waves. The first wave, which began in 1961, was eventually replaced by strains from the second wave in the late 1970s, and the third wave in the early 1990s. While descendants from the second and third wave are both still circulating today, no descendants from the first wave have been isolated after 1992. This lineage can thus be considered as extinct, serving as a well-documented example of a gradually extending "broom branch."

Accordingly, the age of the 7th pandemic selective sweep can be approximated to be around 2025-1980=45 years old, while the third wave is estimated to be approximately 2025-1990=35 years old. Consistent with this timeline, our method of calculating pairwise distances between metagenomes using a consensus clonal frame and molecular clock rate of 1–10 SNPs per genome per year, estimated the age of the entire 7th pandemic sweep to range from 5.1 to 51.5 years, and the age of the third wave to range from 4.6 to 46 years. The neighbor joining tree constructed using pairwise distances also shows that waves 2 and 3 appear as discrete clusters, with the wave 3 cluster emerging out of the wave 2 cluster (**Extended Data Fig. 9b**).

References for Supplementary text

1. Mutreja, A. et al. Evidence for several waves of global transmission in the seventh cholera pandemic. Nature 477, 462–465 (2011).

2. Wang, H. et al. Genomic epidemiology of Vibrio cholerae reveals the regional and global spread of two epidemic non-toxigenic lineages. PLoS Negl. Trop. Dis. 14, e0008046 (2020).

3. Huang, W., Li, L., Myers, J. R. & Marth, G. T. ART: a next-generation sequencing read simulator. Bioinformatics 28, 593–594 (2012).
