# Supplementary material for: Genome-wide sweeps create ecological units in the human gut microbiome
Source: Nature. 2026 May 6;655(8121):202–9. doi: 10.1038/s41586-026-10476-w (PMC13322978; doi:10.1038/s41586-026-10476-w)
Supplement: Supplementary file 2 — Reporting Summary [file 41586_2026_10476_MOESM2_ESM.pdf]

nature portfolio

Corresponding author(s): Martin F. Polz

Last updated by author(s): May 3, 2026

Reporting Summary

Nature Portfolio wishes to improve the reproducibility of the work that we publish. This form provides structure for consistency and transparency in reporting. For further information on Nature Portfolio policies, see our Editorial Policies and the Editorial Policy Checklist.

Statistics

For all statistical analyses, confirm that the following items are present in the figure legend, table legend, main text, or Methods section.

n/a	Confirmed	
☐	☒	The exact sample size (*n*) for each experimental group/condition, given as a discrete number and unit of measurement
☐	☒	A statement on whether measurements were taken from distinct samples or whether the same sample was measured repeatedly
☐	☒	The statistical test(s) used AND whether they are one- or two-sided<br>*Only common tests should be described solely by name; describe more complex techniques in the Methods section.*
☐	☒	A description of all covariates tested
☐	☒	A description of any assumptions or corrections, such as tests of normality and adjustment for multiple comparisons
☐	☒	A full description of the statistical parameters including central tendency (e.g. means) or other basic estimates (e.g. regression coefficient) AND variation (e.g. standard deviation) or associated estimates of uncertainty (e.g. confidence intervals)
☐	☒	For null hypothesis testing, the test statistic (e.g. *F*, *t*, *r*) with confidence intervals, effect sizes, degrees of freedom and *P* value noted<br>*Give P values as exact values whenever suitable.*
☒	☐	For Bayesian analysis, information on the choice of priors and Markov chain Monte Carlo settings
☒	☐	For hierarchical and complex designs, identification of the appropriate level for tests and full reporting of outcomes
☐	☒	Estimates of effect sizes (e.g. Cohen's *d*, Pearson's *r*), indicating how they were calculated

Our web collection on statistics for biologists contains articles on many of the points above.

Software and code

Policy information about availability of computer code

Data collection	No software was used for the microbiome data collection.
Data analysis	All newly sequenced isolates had reads trimmed, filtered and merged with BBMap v.38.90, and assembled via Spades v.3.15.5.<br>All isolate sequences (publically collected and newly sequenced) were quality checked with CheckM v.1.2.2, and assigned to species level taxonomy according to the MetaPhlAn4 reference genome database (version Jan. 2022) using FastANI v.1.33. Genomes were dereplicated according to human subjects using dRep v.3.4.1.<br><br>To estimate recombination fraction in pairwise genomes and call putative genome-wide selective sweeps (GWSSs), we developed a custom software package which we upload at https://github.com/cusoiv/PopCoGenomeS for public use. Curve fitting for the calculation of recombination rates were done using the R package dpseg v.0.1.1.<br><br>For confirmation of the putative genome-wide selective sweeps, we used Mugsy v.1.2.3 followed by ClonalFrameML v.1.12 to extract the clonal frames from each putative GWSS and construct a custom database, and calculated pairwise distances between all (metagenome and isolate) samples using inStrain v.1.7.5 under default settings using the custom database. Calculation of Tajima's D for GWSS clusters was performed using the pegas package (v.1.3) in R.<br><br>For GWSS association studies with the extended metagenome dataset, we used default settings under the accurate mode of StrainPhlAn4 (v. 4.0.6) with the MetaPhlAn4 reference genome database (version Jan. 2022) to perform strain-level metagenome profiling and GWSS cluster identification. Association studies were conducted using the R package SignifReg v.4.3, utilizing a forward feature selection approach. Statistical analyses and graphical representations were performed in R (v.4.2.1) using base R statistical functions and ggplot2 (v.3.5.1), ggpubr (v.0.6.0), ggtree (v.3.4.4), ggtreeExtra (v.1.6.1), and ComplexHeatmap (v.2.12.1).

For the identification of GWSS specific genes, we predicted protein-coding genes in GWSSs with Prodigal v.2.6.3, aligned protein-coding genes in each GWSS against each other with Blast v2.15.0+, and annotated the genes with EggNOG (emapper v.2.1.12, database v.5.0.2) and Prokka (v.1.14.6).

All code for the relevant data analyses above can be found at https://github.com/cusoiv/genome_sweeps.

For manuscripts utilizing custom algorithms or software that are central to the research but not yet described in published literature, software must be made available to editors and reviewers. We strongly encourage code deposition in a community repository (e.g. GitHub). See the Nature Portfolio guidelines for submitting code & software for further information.

Data

Policy information about availability of data

All manuscripts must include a data availability statement. This statement should provide the following information, where applicable:

- Accession codes, unique identifiers, or web links for publicly available datasets
- A description of any restrictions on data availability
- For clinical datasets or third party data, please ensure that the statement adheres to our policy

All newly sequenced genomes are uploaded to NCBI under the BioProject PRJNA1101861. The study and sample accession numbers for all isolate and metagenomes used are available in Tables S5, S6, S9 and S11. Metadata for isolates were collected from the Unified Human Gastrointestinal Genome (UHGG) catalogue (version 1.0) and from the original publications of culturomics studies not included in the catalogue. Metadata for metagenomes were collected from the curatedMetagenomicData 3.4.2 database and from the original publications of studies not included in the database.

Research involving human participants, their data, or biological material

Policy information about studies with human participants or human data. See also policy information about sex, gender (identity/presentation), and sexual orientation and race, ethnicity and racism.

Reporting on sex and gender

We did not perform any sex or gender based analyses because our association studies were only done as examples to illustrate the the ecological differentiation of our genome-wide selective sweep clusters, so we limited our categorical variables to five (age, colorectal cancer, Crohn's disease, Ulcerative colitis, Type 2 diabetes).

Reporting on race, ethnicity, or other socially relevant groupings

We did not conduct a systematic classification based on race, ethnicity, or other socially relevant groupings. We reference the Baka and Beti people in Cameroon and the Matses people in Peru as examples of populations less influenced by industrialization and urbanization. The Baka and the Matses are described as geographically remote hunter-gatherer communities that have never been in direct contact with each other, illustrating that strains in the human gut microbiome can spread rapidly across the world, even to reputedly highly isolated populations. These definitions are based on previous publications containing the relevant microbiome data.

Population characteristics

Relevant metadata for all isolate and metagenomes can be found in Tables S5, S6, S9 and S11.
We analyzed four large sets of data:
1. All publicly available human gut isolate genomes (19,837 in total, from the Unified Human Gastrointestinal Genome (UHGG) catalogue V1.0 and four large-scale culturomics studies), as well as a group of 186 isolates newly collected and sequenced from Austrian individuals. Available participant metadata varied across studies; therefore, we used only two types of metadata: the human subject identifier (used for dereplication of samples from the same individual; when unavailable, the study accession number was treated as the subject identifier) and biogeographical information describing the country or region of sample collection. These metadata are summarized in Table S5.
2. A total number of 1,477 metagenomes representative of 74 datasets from the curatedMetagenomicData 3.4.2 database for the validation of putative genome-wide selective sweep clusters in metagenomes, as well as description of the biogeography of the sweep clusters. The metadata used for metagenome sample selection from the overall dataset was study, age category, disease, and country, which we include in table S6.
3. A total of 118 Vibrio cholerae isolates (for benchmarking analysis) that represent all currently (after 1995) circulating strains of Vibrio cholerae. The metadata used for these isolates are their lineages and pandemic waves, which we include in table S9.
4. A total number of 6,783 metagenome samples comprising 29 datasets from the curatedMetagenomicData 3.4.2 database and other large-scale studies for the 5 host conditions of interest, for the association studies between genome-wide selective sweep clusters and five host conditions. All relevant metadata can be found in table S11.

Recruitment

1. For isolate data, we included all publicly accessible data; they were subsequently dereplicated according to human subjects to avoid false-identification of repeated sampling from the same individual as genome-wide selective sweeps. The group of 186 isolates newly collected and sequenced from Austrian individuals was from patients undergoing colorectal cancer screening colonoscopy at the Vienna General Hospital, and included healthy individuals as well as those with inflammatory bowel disease/ulcerative colitis. Because recruitment occurred in a clinical screening setting, this cohort is enriched for individuals that are relatively older or have inflammatory bowel disease/ulcerative colitis, and therefore may not fully represent the general population. However, these isolates constitute a very small fraction of the total dataset (186 of 20,023 isolates). Moreover, the only participant metadata used in this study were human subject identifiers (for dereplication) and biogeographical information. Therefore, any potential recruitment or self-selection biases associated with this subset are unlikely to meaningfully influence the overall results.
2. Metagenome representatives selected for validation of putative genome-wide selective sweep clusters covered all the datasets available in the curatedMetagenomicData 3.4.2 database. We grouped all samples by study, age category, disease, and country, and selected up to five metagenomes from each unique group combination to ensure that the dataset selected was representative of as many biogeographies and human health states as possible.

	3. Currently circulating (>1995) Vibrio cholerae strains with whole genome sequences and are publicly available were dereplicated based on collection location, year, and pandemic wave.<br>4. On top of a baseline dataset which included 12 datasets from diverse bio-geographies, and had approx. 1:1 ratio between healthy and diseased individuals, we added all the metagenome datasets that were publicly available for the 4 disease conditions we focused on (colorectal cancer, ulcerative colitis, Crohn's disease, type 2 diabetes) for a total of 29 datasets.
Ethics oversight	For the Bacteroides isolate collection, study approval was granted by the ethics committee of the Medical University of Vienna (EK-Nr: 1617/2014, 1910/2019). All study participants gave written informed consent before study inclusion. The study was conducted in accordance with the ethical principles of the Declaration of Helsinki. The analysis of the Global Microbiome Conservancy isolate dataset was conducted with authorized access to data from the database of Genotypes and Phenotypes (accession phs002235.v1.p1), under approval from the US National Human Genome Research Institute.

Note that full information on the approval of the study protocol must also be provided in the manuscript.

Field-specific reporting

Please select the one below that is the best fit for your research. If you are not sure, read the appropriate sections before making your selection.

☒ Life sciences ☐ Behavioural & social sciences ☐ Ecological, evolutionary & environmental sciences

For a reference copy of the document with all sections, see nature.com/documents/nr-reporting-summary-flat.pdf

Life sciences study design

All studies must disclose on these points even when the disclosure is negative.

Sample size	1. For the isolate data, no formal statistical methods were used to predetermine sample size. Instead, we included all publicly available human gut isolate genomes together with genomes we newly generated, resulting in a total dataset of 20,023 isolates.<br><br>2. For the metagenomes used for validation (1,477 samples), the sample size was chosen to mitigate potential isolate sampling bias and expand the number of samples in which each species could be detected relative to isolate-only analyses. To achieve this, we selected stool metagenomes from the curatedMetagenomicData 3.4.2 database representing a broad range of host phenotypes. After dereplication by human subject and stratified subsampling across study, age category, disease status, and country, this procedure resulted in a set of 1,477 metagenomes representing 74 datasets (Table S6). The addition of these metagenomes increased the number of analyzable samples per species by an average of approximately fivefold relative to isolate-only analyses. Furthermore, the number of genome-wide selective sweeps (GWSSs) detected increased with the number of metagenomes included but plateaued when approximately 20–40% of the total 1,477 metagenomes were used (Supplementary Data Fig. 3b), indicating that the current dataset size is sufficient to recover nearly all GWSSs detectable with the available isolate genomes.<br><br>3. For the metagenomes used in our association studies (6783 samples), our goal was to test for associations between genome-wide selective sweeps and 4 disease conditions, as well as advanced age. No formal power calculation was performed; instead, we included all publicly available metagenomic datasets for these conditions to maximize statistical power. We began by constructing a database baseline dataset which included 12 datasets (2,084 samples) from diverse bio-geographies, and had approx. 1:1 ratio between healthy and diseased individuals (654 healthy individuals and 792 individuals with various diseases). We then added all the metagenome datasets that were publicly available for the 4 disease conditions we focused on (colorectal cancer, ulcerative colitis, Crohn's disease, type 2 diabetes) for a total of 6,783 samples from 4,614 individuals (including 646 CRC patients, 749 T2D patients, 467 CD patients, 342 UC patients). Overall, the sample sizes for baseline healthy individuals, baseline diseased individuals, and each of the four disease cohorts are broadly comparable, enabling balanced comparisons in downstream association analyses.
Data exclusions	For isolates, we filtered out all genomes which did not pass a quality screen for completeness and contamination. We dereplicated all isolate samples and metagenomes by human subjects and households, to ensure that we do not falsely detect repeated sampling or transmission within households as genome-wide selective sweeps.
Replication	For the identification and confirmation of genome-wide selective sweeps, we used all publicly available isolate data in the analysis, rendering replication issues inapplicable. Similarly for the association studies, since all publicly available colorectal cancer,ulcerative colitis, Crohn's disease, and type 2 diabetes datasets were included, the replication issue is again inapplicable.
Randomization	Not applicable since this is a cross-sectional study and not a randomized study. No intervention was performed on subjects.
Blinding	Blinding was not applicable for most aspects of this study because the analyses were based on cross-sectional datasets rather than a randomized study design. For the isolates collected from Austrian individuals, the clinicians performing the colonoscopy were not blinded during sample collection because they were directly involved in the clinical procedures. However, downstream culturing and processing of the isolates were performed without knowledge of participant clinical status, minimizing the potential for bias in subsequent experimental steps.

Reporting for specific materials, systems and methods

We require information from authors about some types of materials, experimental systems and methods used in many studies. Here, indicate whether each material, system or method listed is relevant to your study. If you are not sure if a list item applies to your research, read the appropriate section before selecting a response.

Materials & experimental systems

n/a	Involved in the study
☒	☐ Antibodies
☒	☐ Eukaryotic cell lines
☒	☐ Palaeontology and archaeology
☒	☐ Animals and other organisms
☒	☐ Clinical data
☒	☐ Dual use research of concern
☒	☐ Plants

Methods

n/a	Involved in the study
☒	☐ ChIP-seq
☒	☐ Flow cytometry
☒	☐ MRI-based neuroimaging

Plants

Seed stocks	*Report on the source of all seed stocks or other plant material used. If applicable, state the seed stock centre and catalogue number. If plant specimens were collected from the field, describe the collection location, date and sampling procedures.*
Novel plant genotypes	*Describe the methods by which all novel plant genotypes were produced. This includes those generated by transgenic approaches, gene editing, chemical/radiation-based mutagenesis and hybridization. For transgenic lines, describe the transformation method, the number of independent lines analyzed and the generation upon which experiments were performed. For gene-edited lines, describe the editor used, the endogenous sequence targeted for editing, the targeting guide RNA sequence (if applicable) and how the editor was applied.*
Authentication	*Describe any authentication procedures for each seed stock used or novel genotype generated. Describe any experiments used to assess the effect of a mutation and, where applicable, how potential secondary effects (e.g. second site T-DNA insertions, mosiacism, off-target gene editing) were examined.*
