# Supplementary material for: Genome-wide sweeps create ecological units in the human gut microbiome
Source: Nature. 2026 May 6;655(8121):202–9. doi: 10.1038/s41586-026-10476-w (PMC13322978; doi:10.1038/s41586-026-10476-w)
Supplement: Supplementary file 4 — Peer Review File [file 41586_2026_10476_MOESM4_ESM.pdf]

nature portfolio

Peer Review File

Genome-wide sweeps create ecological units in the human gut microbiome

Corresponding Author: Professor Martin Polz

Any redactions in this file are there to maintain patient confidentiality, the confidentiality of unpublished data, or to remove third-party material.

This file contains all reviewer reports in order by version, followed by all author rebuttals in order by version.

Version 0:

Reviewer comments:

Referee #1

(Remarks to the Author)
Summary:

The authors present a fascinating eco-evolutionary analysis of human gut commensal bacterial strains/lineages and their distributions across populations. They address a long-standing question on how adaptive mechanisms sort microbial lineages into ecologically differentiated populations across geographic, demographic, ethnic, and health/disease variation among humans. Prior work has clearly shown genome-wide sweeps in pathogens, which experience strong selective pressure, but less is know about these kinds of sweeps among commensal taxa. Using evolutionary modeling to identify the clonal frame within these commensal genomes, they find widespread evidence for genome-wide sweeps, across a wide regime of recombination rates, in 66 taxa spanning 25 bacterial families. By assuming a range of diversification rates, they are also able to put temporal constraints on the timescales over which these global (cross-continental) sweeps occurred (ranging from decades to thousands of years ago). Finally, the authors show how these sweeps give rise to ecologically-differentiated populations associated with colorectal cancer and with host age.

Originality and significance:

The results are highly original. While these kinds of global genome-wide sweeps have been observed in human pathogens, there is limited evidence for them in human commensals. The scientific significance is that genome-wide sweeps can happen globally across human populations within a matter of decades, and that these events are likely tied to rapid changes in human cultural behaviors and lifestyles (I found the observation that a particular strain was shared across continents among indigenous populations, and absent in all other host populations, with a sweep that must have occurred hundreds of years ago given the degree of diversification, especially compelling). The applied significance centers on the fact that these sweeps show significant association with health and disease states, suggesting that rapid commensal adaptation may be an important factor to consider in the etiology of diseases.

Data and methodology:

The authors assembled 20,023 isolate genomes from the UHGG and from an Austrian cohort, representing a wide variety of taxa isolated from a diversity of human populations. Quality filtering and processing used standard methodologies. Isolates were clustered into species-level genome bins (SGBs) using both ANI and Methaphlan4. For each SGB, only one isolate was retained per host (to avoid sampling multiple closely-related lineages from the same host). After dereplication at the host-level and applying a few quality and metadata filters, 6,411 isolate genomes were retained from 995 SGBs. Next, the authors estimated the recombined and clonal frames of each genome using a combination of maximum likelihood estimations (MLE; poisson + negative binomial model) and hidden markov models (HMMs). Frames were identified by looking for discontinuities in SNP distributions (higher or lower frequencies of SNPs, compared to the genomic background, is evidence for a recombination event) over 500 bp windows, with a rolling window step size of 50 bp. Clonal frame identification was validated using an evolutionary simulation, over a range of parameters (e.g., recombination rates). Genome wide species sweep (GWSS) events were identified by looking for instances where between-lineage genomic

distances were 5X within-lineage distances (i.e., a broom-like structure to the phylogenetic tree with long inter-clade differences separating clusters of isolates that are closely related). Bias in isolate representation was assessed by looking at these 5X signatures metagenomic data. GWSS cluster age was estimated using literature estimates for diversification rates and using direct molecular clock estimates in samples with multiple time points from the same donor. Additional controls and pop-gen statistics were calculated, according to established techniques.

Appropriate use of statustics and treatment of uncertainties:

Statistical analyses were robust, and done using established methods. The authors explored sensitivities of their modeling to parameter variation. They also ran several controls to account for bias and uncertainty in their conclusions (e.g., direct molecular clock estimates for a subset of taxa and estimation of GWSS from metagenomic alignments in addition to isolates).

Conclusions: robustness, validity, reliability:

I believe the conclusions are robust, valid, and reliable, although they occasionally rely upon modeling assumptions. Overall, this is high-quality work that contributes novel insight into adaptive processes and how they shape the human gut microbiota. I have some suggestions below to help improve the manuscript further.

Suggested improvements: clarification questions, minor edits, analyses, data for possible revision:

lines 136-138: You state that over half of sweep clusters contain at least one gene that showed strong signs of positive selection. Did you have a null model for this observation? What if you picked a random set of genomes that did not show evidence of a sweep?

lines 153-154: Is it also possible that these lineages can colonize other hosts at very low frequency and then be transmitted? True absence can be difficult to constrain, especially due to the 'seed bank' effect seen in many marine and sediment microbiomes (e.g., https://www.nature.com/articles/ismej2011162 and https://www.ncbi.nlm.nih.gov/pmc/articles/PMC9826295/).

line 210: Is 'seniority' a common term here? Why not just state 'age'?

lines 221-245: This last section, where the authors look at how GWSS clusters associate with host phenotypes, is the payoff for the prior modeling and analysis work. However, it feels a bit truncated. Why not look at several phenotypes/diseases? The impact of this study could be increased by showing how broadly relevant these adaptive events are to a range of host phenotypes. Can you extract additional 'reverse ecology' insights from existing metadata? Are their broad patterns in the types of genes that are under selection across similar groupings of host phenotypes (e.g., diet-associated, inflammation-associated, etc.)?

lines 273-275: As stated above, it would be great to show more evidence to solidify this point.

line 784: Is 2X coverage sufficient here? Are the results sensitive to this threshold?

lines 955-958: There is a nice GitHub repository for running their GWSS method (including a conda install option). However, the authors do not appear to provide the actual code used to run the specific analyses and to build the figures presented within the manuscript. They should provide this code, so that their work can be directly reproduced by others.

References:

No issues.

Clarity and context:

I commend the authors on a clearly-written manuscript. My only concern is that the authors didn't include a wide-enough analysis of the phenotype associations, which seem to be the most impactful part of this work.

(Remarks on code availability)
As I mentioned in the review, the code for running their popcogenomes method is moderately well documented (and the authors went through the trouble of making this conda-installable, which will improve its usability). However, they should provide an additional GitHub repository that contains the specific code used to run the analyses and build the plots presented in the paper.

Referee #2

(Remarks to the Author)
This is a paper by Yu, Polz and colleagues, investigating selective sweeps in the gut microbiome. The paper reports some interesting analyses, with the most exciting result (in my opinion) suggesting that sweeps can spread across the entire human population within a decade - an important result that can change our understanding of the dynamics of the gut

microbiome. Nevertheless, there are several issues and questions regarding the analytical approaches, that make me less confident in the results and claims.

Major issues:

1. Rules/parameter choice effect on the results: to construct the GWSS clusters, there is a pipeline (outlined in Fig 1a) that relies on quite a few parameters and cutoffs. However, the choice of these parameters and cutoffs seems arbitrary. Then, once the GWSSs are defined, they are used as a ground truth for all the analyses in the paper. However, since the parameters used in the pipeline can be adjusted, I am wondering how changing these parameters and cutoffs can change the resulting GWSS data, thus changing their properties, and the downstream observed biological patterns. Essentially, I am concerned that some or all of the properties that are described are technical artifacts of the bioinformatics pipelines, cutoffs, and parameters used. For example, how does the pipeline for generating the underlying SNP data affect the results? Why were the various cutoffs for identification (e.g., 6%, 5x rule, 50% cutoff, etc) chosen, and what happens to the results if these are changed? I would have much stronger confidence in the results if a range of possible values was tested for each of these parameters, and the effect on the biological results was measured.

2. Age estimation for GWSS: if I understand correctly, the age estimates for GWSS rely on assuming that there are between 1 and 10 SNPs per genome per year, then calculating the age using both 1 and 10, using this as the range (lower and upper limit), and taking the mid-point of the range as the estimated age. Although the approach is overall reasonable, the numbers seem somewhat arbitrary. One main issue is that there are likely different mutation rates for different taxa. It would be good to normalize these numbers based on more accurate, species-specific mutation rates. These mutation rates can be found in the literature, or estimated from the metagenomic data used to confirm the age estimates. This is not perfect, but seems like it could be more biologically precise than assuming a similar range for all taxa. Moreover, mutation rates may vary along the genome. It might be possible to use the metagenomic data and get more accurate estimates by genomic region for each taxon.

Minor points:

The analysis of Tajima’s D in the clusters (page 6) - is this not essentially biased by the approach used to define the clusters?

I would encourage making the code used for the analysis described in the paper publicly available on github, rather than “available upon request”.

(Remarks on code availability)
The code that is made available is just for one aspect of the analysis (the PopCoGenomeS tool). I'd recommend making all the code available, to make it possible to reproduce the analysis and use the code for other purposes.

Referee #3

(Remarks to the Author)
Yu et al. identify putative selective sweeps that happened in the gut microbiomes over time and across global populations by examining both individual isolate genomes and metagenomic bins from microbiomes sequenced from people around the world. The analysis is intriguing, but there are two major issues: first, the use of metagenomes can be appallingly misleading and the authors do not take precautions in examining potential pitfalls in this analysis. Second, there is no ground truth or corroborating evidence that supports their observations, that make it difficult in discerning whether what we are examining is simply artifact of a computational pipeline.

Metagenomic assemblies are riddled with contamination, especially among closely related strains and even species. The authors do not rigorously address this possibility and how it would affect their results. Simulations support the isolate analysis, but less so the possibility of contamination in their metagenomes.

The pathogenic GWSS (they have 11) could be validated using isolate genomes that have been sequenced over time and from around the globe. These could also be applied to validate the fidelity about their assemblies. These 11 include pathogens for which there are many sequenced isolates (i.e. S. enterica, S. aureus, V. cholerae, P. aeruginosa, C. difficile etc.).

A major issue arises in that the authors use a majority nucleotide (consensus clonal frame) approach to identify strains, but these may not be phased accordingly with one another. Specific SNPs may have converged because of function, but represent convergence. The authors should validate their use of consensus SNPs in a rigorous way to prove that it is not biasing their results.

Does the abundance of genomes in samples affect their observations?

How does the number of samples with the SGB affect the likelihood of calling a sweep or the timing of the perceived sweep?

The molecular clock analysis assumes that all organisms have the same mutation rate, whereas some organisms are more actively dividing and maintaining more diversity within their populations. Treating each genome bin with the same molecular clock analysis seems inappropriate.

They cite genes that many bins show strong signs of positive selection (lines 137-138), but are only able to discuss the capsular polysaccharide genes (lines 240) as possibly being selective. What are the genes that the authors find? Are there any logical observations there (for pathogens, perhaps)?

The authors cite genomes that have swept between isolated populations (line 153). How many years do they estimate that this particular sweep occurred?

The authors filter out a lot of sweeps and also samples (Methods). It would help to understand how many samples/sweeps per SGB were being filtered for what reasons and whether any of these filters strongly influences their results. Removing certain samples with added diversity may make the remaining diversity seem more closely related.

I would ask the authors to help align the two notions that these sweeps occur, sometimes over rapid periods (10 years) but that there is not a very strong signal of organismal sharing (within population sharing is extremely low—data coming straight from Segata's previous Nature paper).

Understandably, the timing of the selective sweeps may have occurred 10-10,000 years ago, by these authors' calculations, but the authors could also examine sharing of specific organisms and also calculate whether the number of SNPs expected matches the distribution of SNPs that occur (keeping in mind again, that the majority SNP is likely not a single bona fide strain). These two observations (recent sweeps and the evident, albeit low within population sharing) seem contradictory. Without more proof (many more isolate genomes, long-read sequencing, or corroborating evidence dating these observations), the results seem suspect.

It is unclear what is being shown in Figure 3, and to some extent Figure 4. They show results but now rhyme or reason as to why we these are accurate.

Minor comments:

The curatedmetagenome project has over 10,000 metagenomes. Why did the authors only use less than 1500?

Please explain what "6% diverged" refers to— is this differences in ANI of the entire metagenomic bin? (line 93)

Seniority means age? This is a confusing usage of this word.

Is the Yersinia pathogen in people's microbiomes a misannotation?

Version 1:

Reviewer comments:

Referee #1

(Remarks to the Author)
The authors have done a great job responding to my prior comments.

My only remaining minor comment is that I am having some conceptual trouble with the result showing that genes under positive selection are not enriched in GWSS clusters relative to non-GWSS clusters. Can the authors provide some intuition for this result? I would expect sweeps within the clonal frame to be driven by selection for specific genes/traits within that frame, and that this selection would be detectable (e.g., in terms of dN/dS). Are GWSSs driven by selection acting on the non-clonal frame? Can the authors explain the balance of forces that would drive a global GWSS signature without showing an enrichment for selection effects at specific loci?

(Remarks on code availability)
The code provided appears adequate for reproducing the authors' analyses.

Referee #2

(Remarks to the Author)
I appreciate the authors' extensive efforts to address the parameter sensitivity concerns through a comprehensive robustness analysis. The detailed justification for each cutoff and the systematic evaluation of parameter variations are helpful and represent a major improvement to the manuscript. However, I have remaining concerns:

1. Regarding parameter sensitivity (Comment 2.1): It is good to see that individual parameter variations generally result in modest changes (1-20%) to GWSS detection. Nevertheless, I remain concerned about the impact of these parameter choices. Two important questions:

First, regarding downstream biological implications: Do the 10-20% differences in GWSS numbers translate to meaningful changes in the key biological conclusions? Is it possible that these 10-20% GWSS are different (plausible, considering that they were not detected initially so they may have different properties) and including them can lead to different results? Do the results and conclusions remain consistent across parameter sets? I would encourage examining whether the primary findings are robust to these parameter variations.

Second, regarding combinatorial effects: Although individual parameter changes show modest effects, the cumulative impact of multiple parameter adjustments could be more substantial. Testing a few combinations of parameter changes would strengthen confidence in the overall approach.

2. Regarding mutation rate assumptions (Comment 2.2): I understand the practical limitations in obtaining species-specific mutation rates, and I acknowledge the authors' reasonable approach given available data. However, this limitation highlights a fundamental challenge: the biological accuracy of age estimates remains uncertain due to the broad range (1-10 SNPs/genome/year) and limited taxonomic representation of empirical rates. Although the metagenome-based validation for 9 SGBs is valuable, the uncertainty in mutation rates inevitably propagates through all temporal analyses.

The core overall concern here is not methodological inadequacy, but rather the degree to which multiple unavoidable assumptions may compound to influence the biological interpretations. When the entirety of a study's conclusions rest upon a cascade of methodological assumptions and parameter choices (from SNP calling thresholds to recombination cutoffs to mutation rate estimates) demonstrating that results are robust to these choices becomes essential rather than optional. The compounding uncertainty from multiple assumptions might alter our confidence in the biological interpretations. Without understanding how the primary conclusions change when these parameters vary, we cannot distinguish between genuine biological patterns and methodological artifacts.

Referee #3

(Remarks to the Author)
The responses to reviewers were very thorough. I thank the authors for including the Vibrio analysis and the better explanation of how they use and process the metagenomic data especially vis a vis genome isolates. I have no further questions.

Version 2:

Reviewer comments:

Referee #1

(Remarks to the Author)
The authors have addressed all of my comments.

Referee #2

(Remarks to the Author)
I thank the authors for their comprehensive response and thorough additional analyses. The systematic evaluation of multiple parameter combinations spanning multiple stages of pipeline addresses my concerns about parameter sensitivity and combinatorial effects. The clarification regarding mutation rate estimates is also helpful. The revision has substantially strengthened the manuscript's rigor, and I have no further concerns.

Open Access This Peer Review File is licensed under a Creative Commons Attribution 4.0 International License, which permits use, sharing, adaptation, distribution and reproduction in any medium or format, as long as you give appropriate credit to the original author(s) and the source, provide a link to the Creative Commons license, and indicate if changes were made.
In cases where reviewers are anonymous, credit should be given to 'Anonymous Referee' and the source.
The images or other third party material in this Peer Review File are included in the article's Creative Commons license, unless indicated otherwise in a credit line to the material. If material is not included in the article's Creative Commons license and your intended use is not permitted by statutory regulation or exceeds the permitted use, you will need to obtain permission directly from the copyright holder.
To view a copy of this license, visit https://creativecommons.org/licenses/by/4.0/

Comments to the editor:

We would like to thank the reviewers for the careful reading of our manuscript and for the constructive feedback. We have now revised the manuscript according to the comments, particularly addressing Reviewer #2 and #3's requests to validate the parameters used for genome-wide selective sweep (GWSS) assignments and to benchmark our analyses against known pathogen data, as well as Reviewer #1's suggestions to further develop our phenotype association studies. We feel that these changes have helped improve the clarity, rigor, and potential impact of the manuscript.

Below you will find a point-by-point response to the questions from the reviewers (responses in regular text, comments from reviewers in *italic*). For clarity, we also included a copy of the manuscript where all changes in the text have been marked out in red (related manuscript file).

Referees' comments:

Referee #1 (Remarks to the Author):

Summary:

The authors present a fascinating eco-evolutionary analysis of human gut commensal bacterial strains/lineages and their distributions across populations. They address a long-standing question on how adaptive mechanisms sort microbial lineages into ecologically differentiated populations across geographic, demographic, ethnic, and health/disease variation among humans. Prior work has clearly shown genome-wide sweeps in pathogens, which experience strong selective pressure, but less is know about these kinds of sweeps among commensal taxa. Using evolutionary modeling to identify the clonal frame within these commensal genomes, they find widespread evidence for genome-wide sweeps, across a wide regime of recombination rates, in 66 taxa spanning 25 bacterial families. By assuming a range of diversification rates, they are also able to put temporal constraints on the timescales over which these global (cross-continental) sweeps occurred (ranging from decades to thousands of years ago). Finally, the authors show how these sweeps give rise to ecologically-differentiated populations associated with colorectal cancer and with host age.

Originality and significance:

The results are highly original. While these kinds of global genome-wide sweeps have been observed in human pathogens, there is limited evidence for them in human commensals. The scientific significance is that genome-wide sweeps can happen globally across human populations within a matter of decades, and that these events are likely tied to rapid changes in human cultural behaviors and lifestyles (I found the observation that a particular strain was shared across continents among indigenous populations, and absent in all other host populations, with a sweep that must have occurred hundreds of years ago given the degree of diversification, especially compelling). The applied significance centers on the fact that these sweeps show significant association with health and disease states, suggesting that rapid commensal adaptation may be an important factor to consider in the etiology of diseases.

Data and methodology:

The authors assembled 20,023 isolate genomes from the UHGG and from an Austrian cohort, representing a wide variety of taxa isolated from a diversity of human populations. Quality filtering and processing used standard methodologies. Isolates were clustered into species-level genome bins (SGBs) using both ANI and Methaphlan4. For each SGB, only one isolate was retained per host (to avoid sampling multiple closely-related lineages from the same host). After

dereplication at the host-level and applying a few quality and metadata filters, 6,411 isolate genomes were retained from 995 SGBs. Next, the authors estimated the recombined and clonal frames of each genome using a combination of maximum likelihood estimations (MLE; poisson + negative binomial model) and hidden markov models (HMMs). Frames were identified by looking for discontinuities in SNP distributions (higher or lower frequencies of SNPs, compared to the genomic background, is evidence for a recombination event) over 500 bp windows, with a rolling window step size of 50 bp. Clonal frame identification was validated using an evolutionary simulation, over a range of parameters (e.g., recombination rates). Genome wide species sweep (GWSS) events were identified by looking for instances where between-lineage genomic distances were 5X within-lineage distances (i.e., a broom-like structure to the phylogenetic tree with long inter-clade differences separating clusters of isolates that are closely related). Bias in isolate representation was assessed by looking at these 5X signatures metagenomic data. GWSS cluster age was estimated using literature estimates for diversification rates and using direct molecular clock estimates in samples with multiple time points from the same donor. Additional controls and pop-gen statistics were calculated, according to established techniques.

Appropriate use of statustics and treatment of uncertainties:

Statistical analyses were robust, and done using established methods. The authors explored sensitivities of their modeling to parameter variation. They also ran several controls to account for bias and uncertainty in their conclusions (e.g., direct molecular clock estimates for a subset of taxa and estimation of GWSS from metagenomic alignments in addition to isolates).

Conclusions: robustness, validity, reliability:

I believe the conclusions are robust, valid, and reliable, although they occasionally rely upon modeling assumptions. Overall, this is high-quality work that contributes novel insight into adaptive processes and how they shape the human gut microbiota. I have some suggestions below to help improve the manuscript further.

Response: We thank the reviewer for the positive feedback on our manuscript and for recognizing how it advances our understanding of the adaptive processes that shape the human gut microbiota. We also appreciate the reviewer's thoughtful concerns regarding the appropriate use of population genomics metrics and the suggestion to extract additional 'reverse ecology' insights and broader patterns in the types of genes under selection. We have made the requested revisions accordingly and believe they have significantly improved the quality and scope of our manuscript.

Suggested improvements: clarification questions, minor edits, analyses, data for possible revision:

Rev1-Comment1.1. *lines 136-138: You state that over half of sweep clusters contain at least one gene that showed strong signs of positive selection. Did you have a null model for this observation? What if you picked a random set of genomes that did not show evidence of a sweep?*

Response: We thank the reviewer for this excellent suggestion, which we have addressed as follows. We compared the number of positively selected genes in each genome-wide selective sweep (GWSS) to a null distribution generated from randomly sampled genomes of the same SGB. Specifically, for each commensal sweep cluster containing ⩾3 isolates, we sampled 10 random sets of isolates from the same SGB, matching the number of isolates in the set to that in the corresponding sweep cluster. To be consistent with the methodology for identifying GWSSs, we then restricted the pairwise recombination fraction in the sampled isolates to <50% and

constructed a consensus clonal frame for each sampled set. We finally identified the three closest isolates to the tMRCA of each sampled set, and calculated dN/dS for all genes in the consensus clonal frame compared to each of the three closest isolates. Among all commensal GWSSs containing ⩾3 isolates, 38 had sufficient isolates within their respective SGBs to allow random sampling. We recorded the number of genes with dN/dS > 2 in all sampled sets to build the null distribution. We then compared this null distribution to the observed number of genes with dN/dS > 2 in the commensal GWSSs, and found that sweep clusters do not contain significantly more genes under positive selection than expected by chance (**Figure R1.1.1**).

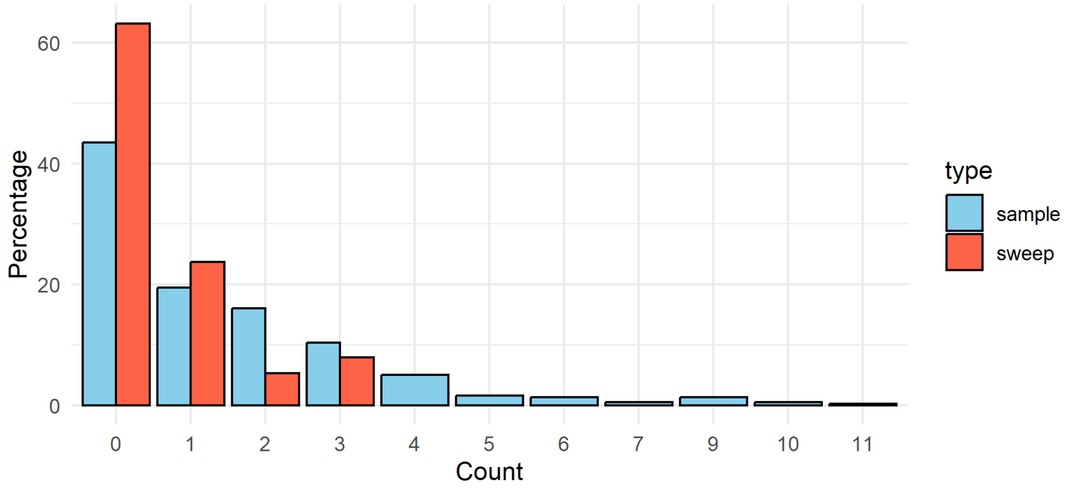

Figure R1.1.1 Histogram for the number of genes with dN/dS>2 in 38 commensal GWSSs compared to randomly sampled sets of isolates.

However, in hindsight, we realized that GWSS clusters are not necessarily expected to be enriched for genes under positive selection compared to randomly sampled clusters of the same size. The presence of more positively selected genes within a GWSS cluster neither implies stronger genome-wide positive selection, nor does the presence of positively selected genes indicate that a genome-wide sweep has occurred. Therefore, rather than serving as evidence for genome-wide sweeps, positively selected genes may be more appropriately used to identify candidate genes that could have contributed to the selective event that formed GWSSs, or represent genes that were under selection post sweep.

Actions taken:

Main text: We removed the analysis that quantified the number of genes with dN/dS > 2 in each GWSS (Extended Data Fig. 4b, lines 136-138 and lines 782-790 in previous manuscript), as this metric does not support the occurrence of genome-wide selective sweeps. Instead, we incorporated the characterization of genes under positive selection into the "**GWSS clusters represent ecologically differentiated populations**" section (Line 241). This allows us to use the functional annotations of differentially positively selected genes as potential support for ecological differentiation among GWSSs.

Supplementary Tables: We now include all genes under positive selection in all GWSS clusters in Supplementary Table S3.

Rev1-Comment1.2. *lines 153-154: Is it also possible that these lineages can colonize other hosts at very low frequency and then be transmitted? True absence can be difficult to constrain, especially due to the 'seed bank' effect seen in many marine and sediment microbiomes (e.g., https://www.nature.com/articles/ismej2011162 and https://www.ncbi.nlm.nih.gov/pmc/articles/PMC9826295/).*

<u>Response & actions taken</u>: We agree that this is a possibility and have therefore revised lines 153 -159 in the **main text** to reflect this.

Rev1-Comment1.3. *line 210: Is 'seniority' a common term here? Why not just state 'age'?*

<u>Response & actions taken</u>: Thank you, we now use advanced age (>65) as a more precise definition than seniority in the corresponding line (now line 218) and throughout the paper.

Rev1-Comment1.4. *lines 221-245: This last section, where the authors look at how GWSS clusters associate with host phenotypes, is the payoff for the prior modeling and analysis work. However, it feels a bit truncated. Why not look at several phenotypes/diseases? The impact of this study could be increased by showing how broadly relevant these adaptive events are to a range of host phenotypes. Can you extract additional 'reverse ecology' insights from existing metadata? Are their broad patterns in the types of genes that are under selection across similar groupings of host phenotypes (e.g., diet-associated, inflammation-associated, etc.)? and lines 273-275: As stated above, it would be great to show more evidence to solidify this point.*

<u>Response</u>: We agree with the reviewer that looking at more phenotypes/diseases, as well as finding common patterns in the types of genes that are under selection, would significantly increase the quality and impact of the paper. Since diet data associated with metagenomes are often missing or unreliable, we expanded our search only for disease associated GWSSs and for broad patterns in genes that are unique to each GWSS in the following ways:

1. **Increase of host states.** We expanded our analysis beyond the original advanced age (>65) and CRC to now include type II diabetes (T2D) and two inflammatory bowel diseases, ulcerative colitis (UC) and Crohn's disease (CD). This expansion increased our metagenome database from 17 datasets containing 3,941 metagenomes to a total of 29 datasets and 6,783 metagenomes. With this expansion, we now shift our focus from identifying distinct GWSSs within each SGB that associate in opposite directions with a single disease, to identifying GWSSs within each SGB that positively associate with different diseases.
2. **Broad patterns in genes under selection in GWSSs.** We examined genes unique to each GWSS cluster and found that, relative to all genes present in GWSS clusters within the corresponding SGB, there was significant and consistent differential enrichment for mobile genetic elements, genes involved in cell wall and membrane biogenesis, and glycosyltransferases. These gene categories frequently co-localize, suggesting they may collectively encode for the synthesis of capsular polysaccharides. This finding is consistent with our analysis of *Bacteroides fragilis* in the initial manuscript, where we observed similar functional enrichments. Importantly, these enrichments are not restricted to specific disease-related GWSSs; rather, they are a common feature across GWSSs independent of host condition (see revised Fig. 4c). These results suggest that capsular polysaccharides may play a central role in facilitating host condition-specific adaptation. No significant functional enrichments were found for genes under positive selection.

<u>Actions taken</u>:

Main Figures: We updated Figure 4 so that it now includes:

a) A "volcano plot" summarizing all GWSSs associated with different diseases, with the x-axis being the association coefficient, and the y axis being the -log10(p value) of the association. The associated GWSSs are further classified based on whether they exhibit geographic bias.

b) As an illustration, we show GWSSs in *Bacteroides uniformis* that are differentially positively associated with all five host conditions tested.
c) A bar plot showing the enrichment of genes involved in cell wall and membrane biogenesis (COG category M), as well as glycosyltransferases, among genes unique to GWSSs relative to their abundance in all GWSSs and demonstrating that this enrichment is independent of disease associations.

Main text: We revised the section "**GWSS clusters represent ecologically differentiated populations**" (lines 211-254) to emphasize that distinct GWSSs within the same SGB can be associated with different diseases, and that these associations may be driven by differences in capsular polysaccharide composition. We also updated the methods section "**Association studies of GWSS clusters in metagenomes**" (lines 944-988) to reflect the expansion of the overall dataset and adjustments to filtering criteria to accommodate the increased number of disease phenotypes. The major change was in the analysis of age-related associations: rather than comparing people in the advanced age (>65) group and reference age group irrespective of health status, we now restrict the association analysis to healthy individuals within each group. This refinement allows us to specifically assess the impact of age on the gut microbiome independent of disease. We also revised the relevant parts in the abstract (lines 36-38) and discussion (lines 261-262) accordingly.

Supplementary Figures: We revised the previous Extended Data Fig. 8 so that it no longer summarizes SGB-level and country-specific GWSS associations with CRC and seniority. Instead, it (now Extended Data Fig. 7) is a summary of non-regional GWSS associations across the five currently evaluated host states. The SGB-level associations with these five host states are now provided separately in the new Extended Data Fig. 8.

Rev1-Comment1.5. *line 784: Is 2X coverage sufficient here? Are the results sensitive to this threshold?*

<u>Response</u>: We increased the coverage threshold to 4X and observed only minor differences in the number of genes with dN/dS > 2 in each GWSS compared to the original 2X threshold (**Figure R1.5.1**). However, since the relevant analysis has been removed from the manuscript, we did not further explore additional coverage thresholds.

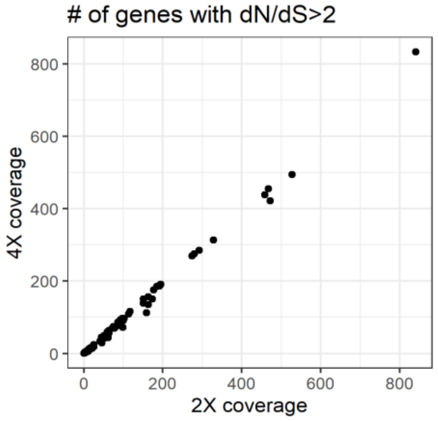

Figure R1.5.1 Number of genes with dN/dS>2 in each SGB under 4X vs. 2X coverage thresholds. Each dot represents one SGB.

Rev1-Comment1.6. *lines 955-958: There is a nice GitHub repository for running their GWSS method (including a conda install option). However, the authors do not appear to provide the actual code used to run the specific analyses and to build the figures presented within the manuscript. They should provide this code, so that their work can be directly reproduced by others.*

Actions taken: We have added this code to GitHub. The additional code is in a separate GitHub repository (https://github.com/cusoiv/genome_sweeps), and includes sample scripts for generating clonal frames and mapping metagenomes individually to each clonal frame, as well as code for all subsequent analyses to generate all the figures in the paper.

References: *No issues.*

Clarity and context: *I commend the authors on a clearly-written manuscript. My only concern is that the authors didn't include a wide-enough analysis of the phenotype associations, which seem to be the most impactful part of this work.*

Response: Thank you, we hope we have resolved the issues with our responses and actions above.

Referee #1 (Remarks on code availability): *As I mentioned in the review, the code for running their popcogenomes method is moderately well documented (and the authors went through the trouble of making this conda-installable, which will improve its usability). However, they should provide an additional GitHub repository that contains the specific code used to run the analyses and build the plots presented in the paper.*

Response & actions taken: Thank you, our new GitHub repository (https://github.com/cusoiv/genome_sweeps) now includes the specific code used to analyze data and build plots in the paper. We keep the original https://github.com/cusoiv/PopCoGenomeS repository reserved only for the PopCoGenomeS tool for clarity.

Referee #2 (Remarks to the Author):

This is a paper by Yu, Polz and colleagues, investigating selective sweeps in the gut microbiome. The paper reports some interesting analyses, with the most exciting result (in my opinion) suggesting that sweeps can spread across the entire human population within a decade - an important result that can change our understanding of the dynamics of the gut microbiome. Nevertheless, there are several issues and questions regarding the analytical approaches, that make me less confident in the results and claims.

Response: We thank the reviewer for the careful and thoughtful reading of the manuscript and for raising important concerns regarding how our choice of parameters and rules could affect the results. We have now performed a more comprehensive analysis and believe these strengthen the scientific rigor of the paper.

Major issues:

Rev2-Comment 2.1. *Rules/parameter choice effect on the results: to construct the GWSS clusters, there is a pipeline (outlined in Fig 1a) that relies on quite a few parameters and cutoffs. However, the choice of these parameters and cutoffs seems arbitrary. Then, once the GWSSs are defined, they are used as a ground truth for all the analyses in the paper. However, since the parameters used in the pipeline can be adjusted, I am wondering how changing these parameters and cutoffs can change the resulting GWSS data, thus changing their properties, and the downstream observed biological patterns. Essentially, I am concerned that some or all of the properties that are described are technical artifacts of the bioinformatics pipelines, cutoffs, and parameters used. For example, how does the pipeline for generating the underlying SNP data affect the results? Why were the various cutoffs for identification (e.g., 6%, 5x rule, 50% cutoff, etc) chosen, and what happens to the results if these are changed? I would have much stronger confidence in the results if a range of possible values was tested for each of these parameters, and the effect on the biological results was measured.*

Response: We appreciate this comment from the reviewer, and we recognize the importance of additional demonstrations of the robustness and sensitivity of the sweep detection to the parameters used in the SNP calling pipeline as well as the various cutoffs applied during identification. Some of these parameters were already examined and justified in the original manuscript but we also summarize these in the table below for clarity. For others, we recognize that further evaluation was necessary and have therefore conducted additional analyses to assess how changes in these parameters affect GWSS detection, trying to include all parameters even if not explicitly listed by the reviewer. Below is a table summarizing both the parameters previously evaluated in the manuscript and those newly examined, along with their corresponding impact on GWSS identification. In the section following the table, we detail the actions taken based on the analysis of the parameters.

Table R2.1.1 – Summary of parameters used for calling GWSSs and assessing their impact on GWSS detection

Parameter	Used	Alterations/Explanations
ANI cutoff for species assignment	94% ANI,<br>>30% coverage	The rationale for using such a fairly relaxed species-calling metric was explained in detail in lines 518-533 of the previous manuscript (now lines 531-547). We believe it is **unnecessary to alter this threshold**, as the maximum within-sweep distance observed across all detected GWSSs is 0.62%, while the distance to its closest relative is 3.1%. Thus, changing the species-level cutoff would have minimal impact on sweep

		detection, since GWSSs occur at much finer scales compared to the species boundary.
Cutoff to determine vertical inheritance	<50% recombination	Since this recombination cutoff was mainly used to identify putative GWSSs based on isolate genomes and to ensure that phylogenies predominantly reflect vertical inheritance, we compared how the number of putative GWSSs changes when the recombination cutoff for calling GWSSs is adjusted to 33% and 67%, respectively. As shown in **Figure R2.1.1,** 292 of 377 putative GWSSs are identical across all three cutoffs, while modifying the cutoff alters both the total number of GWSSs and the number of isolate genomes included in the GWSSs by approximately 10–20%. Increasing the recombination cutoff to 67% allows slightly more GWSSs (and the isolate genomes within them) to be recovered, but this comes at the cost of having a smaller clonal frame for downstream analyses, which could reduce the accuracy of distance calculations between samples. Conversely, lowering the recombination cutoff to 33% would allow for the inclusion of larger clonal frames but at the cost of losing some GWSSs. Because recombination can quickly overwrite clonal frames, a situation may arise where some genomes may artificially be excluded from the cluster. For example, a GWSS identified at a 50% cutoff may include a genome that shares a substantial clonal fraction (>50% but <67%) with the rest of the cluster. Reducing the recombination cutoff to 33% would falsely exclude this genome, reclassifying it as the closest relative rather than a member of the GWSS, thus preventing the identification of the original sweep.<br><br>We therefore chose to **maintain 50% as the cutoff**, as it represents a reasonable balance between losing a small number of putative GWSSs and minimizing the risk of less accurate downstream distance calculations.
Fold cutoff for GWSSs	5X	The 5X cutoff, as explained in the main text (Lines 657–665 of previous manuscript, currently lines 673-679), represents a stricter variant of the previously published 4X rule, reducing the probability of an identified GWSS actually being the result of evolutionary drift to less than 1%. We **deem this 5X cutoff sufficiently strict**, especially since even higher thresholds recover the majority of the GWSSs (e.g., 6X and 8X retain 89% and 75% of the GWSSs, respectively).
Reference genome to represent	Consensus clonal frame	As explained in detail in the response to **Rev3-Comment 3.3,** using the consensus clonal frame as the representative for isolate-based sweeps had minimal impact on both GWSS identification and the estimation

isolate-based GWSSs		of GWSS age when compared to using clonal frames derived from randomly sampled isolates within the GWSS. Therefore, we have chosen to **continue using the consensus clonal frame** for this purpose.
# of Metagenomes used for GWSS detection	1,477	To ensure that the number of GWSSs detected was not underestimated due to an insufficient number of metagenomes, we assessed whether GWSS detection was sensitive to the number of metagenomes used. Specifically, we performed a rarefaction analysis by subsampling the full set of 1,477 metagenomes used in the study to 0%, 10%, 20%, 40%, 60%, and 80%, and then counted the total number of GWSSs detected at each level. As shown in the rarefaction curve below in **Figure R2.1.2**, the number of GWSSs detected increases with the number of metagenomes included, but this trend saturates between 20–40% of the currently used set of metagenomes. These results suggest that the **number of metagenomes used in the current analysis is already sufficient to recover all GWSSs detectable with the available isolate genomes**. While additional GWSSs may exist, their discovery is likely limited by the availability of isolate genomes rather than by metagenome sample size.
Cutoff for mixed vs. single strain	5th percentile of the allele frequency spectrum exceeded 0.8	As explained previously in lines 714-721 of the manuscript (now lines 728-736), this is to ensure that the calculation of sequence distance between the metagenomes and the consensus clonal frame (CCF) does not represent a mixture of strains. This cutoff allows up to 10% of a minor strain to exist in the metagenome: it prevents excessive filtering of metagenomes while minimizing the risk of introducing artificial signals from samples that contain multiple strains. We therefore **maintain this threshold**, as it balances accuracy and inclusivity well.
Metagenome/Isolate to CCF distance (coverage depth)	Only samples with average ≥2X coverage of the CCF are used to calculate the distance	We had previously selected a relatively low cutoff (2X) to ensure that we do not miss any close relatives to the isolate-based GWSSs. To assess the sensitivity of GWSS identification to this parameter, we tested both a more stringent (4X) and a more relaxed (1X) cutoff. We found that these adjustments reduced the number of GWSSs identified by less than 5% (124→121 for 1X, and 124→120 for 4X, **Figure R2.1.3**), with 103 out of the 124 GWSSs being consistently identified across all three thresholds. Given the robustness of GWSS detection to changes in coverage depth, we chose to **maintain the 2X coverage cutoff** as a compromise: it minimizes the risk of missing close relatives due to overly strict coverage requirements while also limiting the potential for contamination from low-coverage reads.

Metagenome/Isolate to CCF distance (coverage breadth)	Only samples with sufficient average coverage breadth of the CCF are included in distance calculations; the threshold is 25% for metagenomes and 50% for isolates.	We investigated how changing the coverage breadth cutoffs to 25% for both isolates and metagenomes, or to 50% for both, would affect the number of GWSSs identified. The cutoff was originally set lower for metagenomes (25%) to avoid missing strains with lower coverage, while it was set higher for isolates (50%) to ensure that any strains potentially misassigned to the SGB would be filtered out. We found that using the 25% threshold for both isolates and metagenomes would decrease the GWSSs by less than 1% (124 →123), while using the 50% threshold would decrease it by 2.4% (124 →121, **Figure R2.1.3)**. Therefore, GWSS detections are also robust to changes in coverage breadth thresholds, and we therefore choose to **maintain the original cutoff**.
Pairwise distances between samples	Only calculate the distance between samples that cover the same reference CCF by >25%	We had previously selected a relatively low cutoff (25%) to ensure that we captured more pairs spanning both within-GWSS and outside-GWSS samples. We therefore tested if shifting to a more stringent cutoff (50%) would change the number of GWSSs identified, and find it increases the number of GWSSs by 3.2% (124→128, **Figure R2.1.3)**. However, we cannot determine whether any of these additional GWSSs were the results of a more stringent cutoff. We therefore **maintain our original coverage cutoffs**, as raising it does not recover many additional GWSSs and allows us to take a more cautious approach.

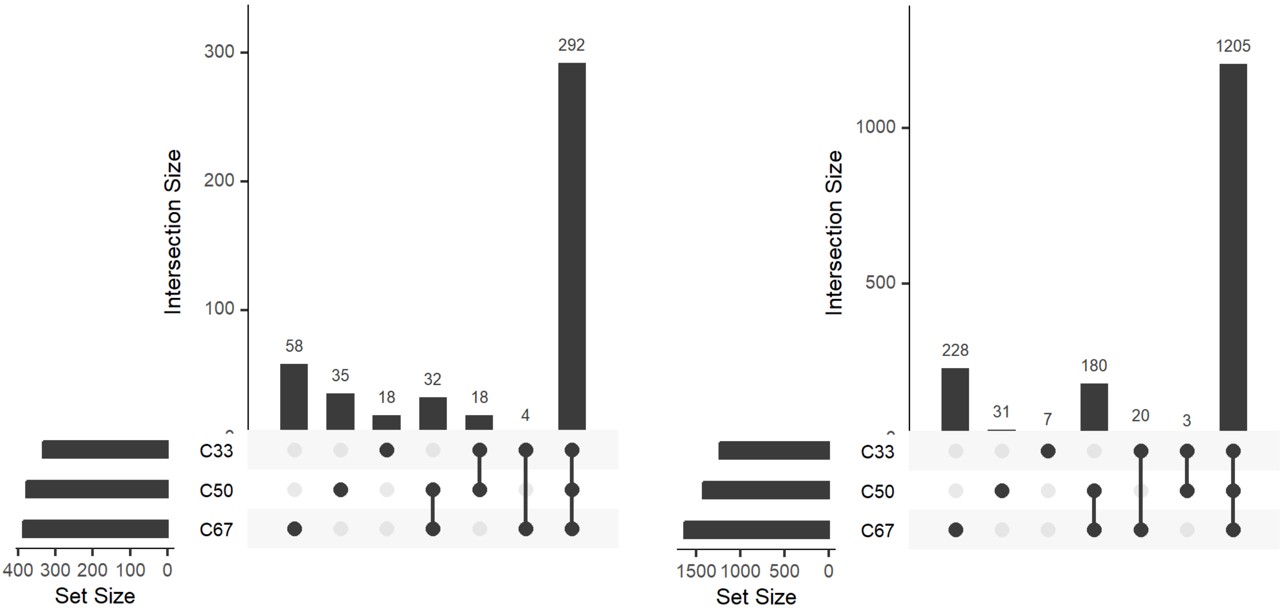

Figure R2.1.1 Comparison of the number of isolate-based sweeps (left) and the corresponding isolates within them (right) detected under different recombination cutoffs. In each UpSet plot, the horizontal bars on the left represent the total number of isolate-based

sweeps (or isolate samples within sweeps) detected under three recombination fraction thresholds, respectively: C33 (<33%), C50 (<50%), and C67 (<67%). The vertical bars represent the number of sweeps (or samples) shared across different thresholds, as indicated by the filled dots below each bar.

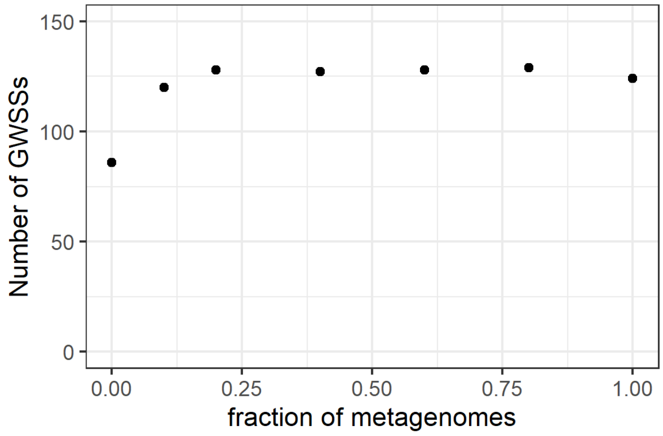

Figure R2.1.2 Rarefaction curve showing the number of GWSSs detected as a function of the fraction of metagenomes used. The x-axis indicates the proportion of the 1,477 metagenomes included in the analysis, and the y-axis shows the total number of GWSSs identified at each subsampling level.

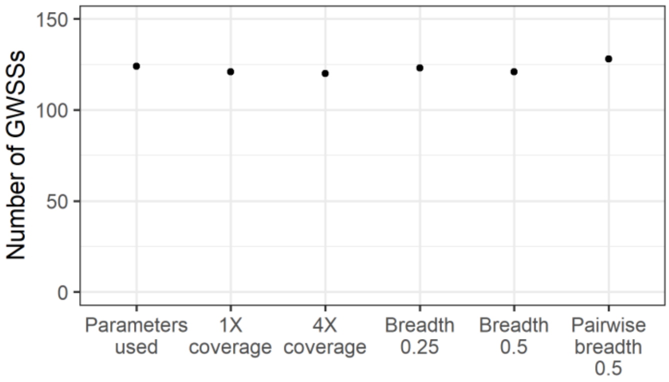

Figure R2.1.3 Comparison of the number of GWSSs identified under different coverage cutoffs. "Parameters used" shows the number of GWSSs detected using the parameters described in the manuscript. "1X coverage" and "4X coverage" indicate the # of GWSSs found when the minimum average coverage required for a sample mapping to a CCF was changed from 2X to 1X or 4X for calculating the distance between the sample and the CCF. "Breadth 0.25" and "Breadth 0.5" represent analyses where the minimum coverage breadth required for both isolates and metagenomes mapping to CCFs was increased to 25% and 50%, respectively. "Pairwise breadth 0.5" shows the number of GWSSs detected when the minimum shared fraction between samples covering the same reference CCF was increased from >25% to 50% when calculating pairwise distances between the samples.

<u>Actions taken</u>:

In summary, we evaluated the sensitivity of GWSS identification to adjustments of key parameters used to detect them. In analyses beyond the original evaluations, we focused on three main parameter types: (1) how the recombination fraction cutoff used to define vertical inheritance affects the detection of isolate-based sweeps; (2) how the number of metagenomes included influences the number of GWSSs detected; and (3) how changes to coverage cutoffs, both in depth and breadth, for samples mapping to CCFs affect GWSS detection. We found that varying the recombination and coverage cutoffs (parameter types 1 and 3) changed the number of isolate-based sweeps and GWSSs by approximately 1–20%. We therefore **retain the original values**, as these results demonstrate that GWSS detection is generally robust to parameter changes. Moreover, while some settings may recover a small number of additional GWSSs, they also increase the risk of false positives. For parameter 2, rarefaction analysis showed that while the number of GWSSs detected rises with more metagenomes included, this trend plateaus

between 20–40% of the total dataset, indicating that the current metagenomic dataset is sufficiently large and that **introducing additional metagenomes is unnecessary**.

Main text: We have added a section in methods called the "**Robustness of GWSS assignments**" (Lines 781-795) for a description of the three major types of parameters that we newly evaluated for their impact on calling GWSSs, and their consequences.

Supplementary tables and Figures: The parameters adjusted, and their consequences are summarized and described in more detail in Table S8, while Figures R2.1.1, R2.1.2, and R2.1.3 are added to the manuscript as Supplementary Data Fig. 2.

Rev2-Comment 2.2. *Age estimation for GWSS: if I understand correctly, the age estimates for GWSS rely on assuming that there are between 1 and 10 SNPs per genome per year, then calculating the age using both 1 and 10, using this as the range (lower and upper limit), and taking the mid-point of the range as the estimated age. Although the approach is overall reasonable, the numbers seem somewhat arbitrary. One main issue is that there are likely different mutation rates for different taxa. It would be good to normalize these numbers based on more accurate, species-specific mutation rates. These mutation rates can be found in the literature, or estimated from the metagenomic data used to confirm the age estimates. This is not perfect, but seems like it could be more biologically precise than assuming a similar range for all taxa. Moreover, mutation rates may vary along the genome. It might be possible to use the metagenomic data and get more accurate estimates by genomic region for each taxon.*

Response: While we also had hoped to find more taxa specific molecular clocks, this type of data was scarcer than anticipated. The estimate of 1-10 SNPs per genome per year is based on the currently available literature[1–4] , that estimated point mutation rates for bacteria *in vivo* in a rigorous and isolate-based manner. Even so, most of these estimates come from pathogenic bacteria, and the only estimate made for gut commensal bacteria *in vivo* is *Bacteroides fragilis* (1 SNP per genome per year).

To make up for this lack of measured rates, we **included in the paper an estimated metagenomic molecular clock for 9 SGBs based on all available metagenomic time series data or twin metagenomes we could access**. These molecular clocks were estimated by identifying strains that persisted in individuals over time or were shared between twins, then calculating the number of SNPs separating these strains divided by the years between sampling (or the time twins had been living apart, under the assumption that they initially shared the same strains). However, we could only investigate 9 SGBs in this way because we were limited to SGBs with sufficient coverage and detectable shared strains across time points (twin samples). Overall, these metagenome-based estimates were consistent with the range of 1–10 SNPs per genome per year (Figure 2b). Although we hope that future work will expand this analysis to more SGBs and integrate both isolate-based and metagenome-based molecular clocks, current efforts remain constrained by the availability of suitable isolate genomes and longitudinal metagenome data. While it would have been possible to incorporate these metagenome-derived molecular clocks into the age estimations directly, we ultimately **chose to present them as a separate benchmarking analysis to avoid introducing inconsistencies within the main age estimation figure** (Figure 2a).

Minor points:

Rev2-Comment 2.3. *The analysis of Tajima's D in the clusters (page 6) - is this not essentially biased by the approach used to define the clusters?*

Response: We do not think that the analysis is essentially biased by the approach to define the clusters. While the method to define the clusters does select for groups of genomes with lower average divergence within the cluster compared to genomes outside the cluster, this does not

necessarily guarantee that the SNP distribution within the cluster will be homogeneous among different genomes. Tajima's D, which quantifies the average pairwise nucleotide diversity relative to that expected under a neutral model that assumes that SNPs are randomly distributed among genomes at a constant mutation rate, tests whether there is an excess of low-frequency variants (i.e., many singletons) relative to the total diversity[5]. A significantly negative Tajima's D indicates such an excess and is consistent with recent rapid population growth or positive selection, often reflecting recovery from a recent clonal expansion[6]. In this sense, Tajima's D provides an orthogonal measure to the 5X rule, offering an independent line of evidence to support the identification of true genome-wide selective sweeps.

Action taken: We include a better explanation for Tajima's D now in lines 136-138 to help the reader better understand why this population genetic measurement can be indictive of genome-wide sweeps and are orthogonal to the 5X rule.

Rev2-Comment 2.4. *I would encourage making the code used for the analysis described in the paper publicly available on github, rather than "available upon request".*

Response & actions taken: We have added this code to GitHub. The additional code is in a separate GitHub repository (https://github.com/cusoiv/genome_sweeps), and includes sample scripts for generating clonal frames and mapping metagenomes individually to each clonal frame, as well as code for all subsequent analyses to generate all the figures in the paper.

Referee #2 (Remarks on code availability):

The code that is made available is just for one aspect of the analysis (the PopCoGenomeS tool). I'd recommend making all the code available, to make it possible to reproduce the analysis and use the code for other purposes.

Response & actions taken: Thank you, our new GitHub repository ((https://github.com/cusoiv/genome_sweeps) now includes the specific code used to analyze data and build plots in the paper. We keep the original https://github.com/cusoiv/PopCoGenomeS repository reserved only for the PopCoGenomeS tool for clarity.

Referee #3 (Remarks to the Author):

Yu et al. identify putative selective sweeps that happened in the gut microbiomes over time and across global populations by examining both individual isolate genomes and metagenomic bins from microbiomes sequenced from people around the world. The analysis is intriguing, but there are two major issues: first, the use of metagenomes can be appallingly misleading and the authors do not take precautions in examining potential pitfalls in this analysis. Second, there is no ground truth or corroborating evidence that supports their observations, that make it difficult in discerning whether what we are examining is simply artifact of a computational pipeline.

Response: We thank the reviewer for the acknowledgement of the significance of our work and for raising important concerns about potential pitfalls in metagenomic analyses and the need for stronger benchmarking. Although we do not use metagenomic bins or assemblies, we acknowledge that some issues can apply to metagenomic data more broadly. We have therefore addressed these concerns and added benchmarking with pathogen data, which we believe significantly improve the confidence in our results.

Rev3-Comment 3.1. *Metagenomic assemblies are riddled with contamination, especially among closely related strains and even species. The authors do not rigorously address this possibility and how it would affect their results. Simulations support the isolate analysis, but less so the possibility of contamination in their metagenomes.*

Response: Metagenome contamination is typically an issue when assembling MAGs or predicting strains. However, our approach does not use metagenomic assemblies but instead only uses isolate genomes to initially identify putative sweep clusters and then uses metagenomes to verify those by mapping reads to these sweeps. Nonetheless, we agree that we had not rigorously evaluated the potential impact of co-occurrence of closely related strains (at low-levels) on predicting sweeps, which we refer to below as artificial contamination.

To address this potential concern, we focused on the largest consensus clonal frame (CCF) database used for the validation of putative GWSS clusters in metagenomes (the largest of six databases, containing 75 CCFs that corresponds to isolate-based, putative sweeps) and introduced artificial contamination from closely related strains into all metagenomes used for sweep confirmation. Specifically, for each metagenome, we added simulated reads derived from the SGBs containing the isolate-based sweeps at 0.5X coverage of an isolate randomly sampled within the SGB. We then assessed how such contamination affected the total number of GWSSs called, as well as two key metrics for the GWSSs: (1) the maximum divergence of the sweep, and (2) the distance between the sweep and its closest relative. This analysis allowed us to evaluate the robustness of our sweep detection approach in the presence of low-level contamination from closely related genomes that are not part of the sweep cluster.

We found that the introduction of this contamination did not alter the number or identity of the GWSSs detected. Examination of two key parameters, the maximum divergence within the GWSS (**Figure R.3.1.1**, left) and the distance of the GWSS to the closest relative (right) indicated that these distances also changed very minimally. Overall, this analysis suggests that low-level contamination from closely related strains has a limited impact on GWSS detection and does not substantially affect the robustness of our approach.

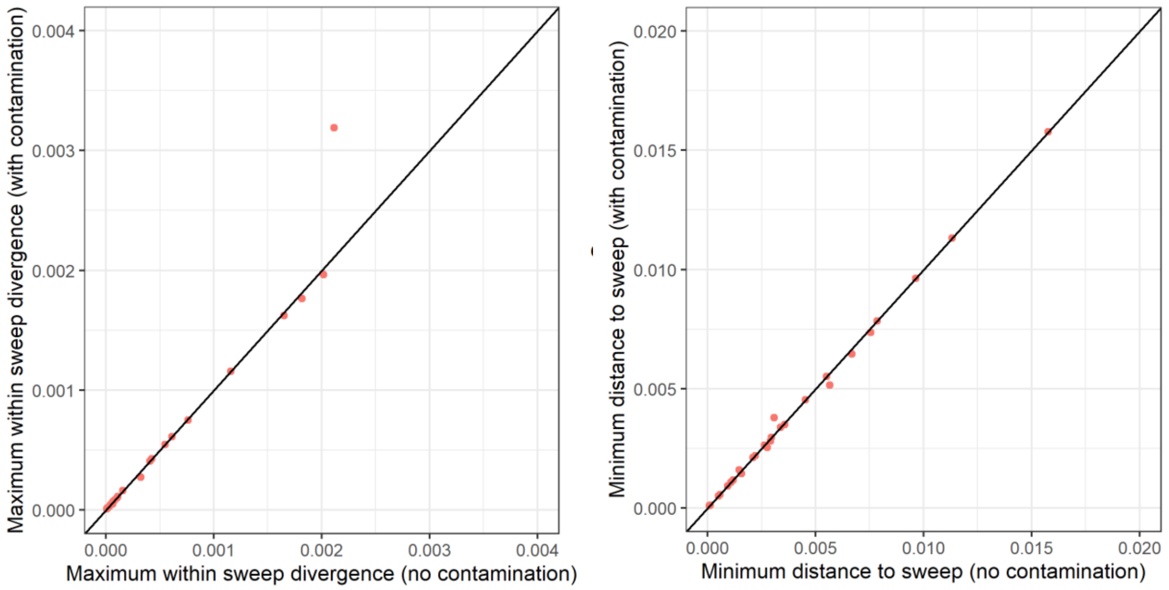

Figure R3.1.1 Contamination effects on maximum divergence within GWSSs (left) and distance of the GWSS to the closest relative (right). Each dot represents a GWSS identified after the introduction of contamination.

<u>**Actions taken**</u>**:**

Main text: We have added a section in methods called the "**Sensitivity of GWSS assignments to low-level occurrence of closely related strains**" (Lines 796-805) to describe the analysis above and summarize the results.

Supplementary Figures: Figure R3.1.1 is added to the paper as Supplementary Data Fig. 3.

Rev3-Comment 3.2. *The pathogenic GWSS (they have 11) could be validated using isolate genomes that have been sequenced over time and from around the globe. These could also be applied to validate the fidelity about their assemblies. These 11 include pathogens for which there are many sequenced isolates (i.e. S. enterica, S. aureus, V. cholerae, P. aeruginosa, C. difficile etc.).*

<u>**Response**</u>**:** We agree with the reviewer that validating our method using pathogens with temporally sampled isolates is an excellent idea. We selected *Vibrio cholerae* for this validation because it is a pathogen that colonizes the human gut with well-documented clonal expansions and global waves of transmission events, as well as an estimated molecular clock of approximately 3.3 SNPs per genome per year based on historical isolates ($8.3*10^{-7}$ SNPs $site^{-1}$ $year^{-1}$)[4]. But again, we would like to point out that we do not use metagenome assemblies.

1. **Validation of the 7th pandemic as a GWSS.** The ongoing 7th pandemic of *V. cholerae*, comprising all currently circulating pandemic strains, has been shown in previous studies to originate from a single source population in the Bay of Bengal, followed by local diversification and eventual extinction outside endemic regions[4]. These 7th pandemic strains form a distinct clonal group, known as the L2 phyletic lineage. Given these features, all modern *V. cholerae* L2 isolates (i.e. collected after 1995) should be identifiable as a GWSS.

   To validate that our method is capable of detecting *V. cholerae* from the 7th pandemic as a GWSS, we constructed a dataset of L2 isolates collected after 1995 and dereplicated them based on collection location, year, and pandemic wave (as it is known that the 7th pandemic consists of three distinct global transmission waves) to avoid oversampling of

individual outbreaks[4]. In parallel, we assembled a dataset of non-L2 *V. cholerae* clinical isolates collected after 1995, similarly dereplicated by location, year, and lineage[7]. The 1995 cutoff was chosen because many historical isolates (and their associated lineages) are likely extinct, and their absence is part of what enables genome-wide sweeps to be detected. Including such lineages would confound GWSS identification.

Finally, due to the scarcity of metagenomes from cholera patients, and to mimic the isolate–metagenome framework used to identify GWSSs in our study, we simulated mock metagenomes by converting each *V. cholerae* isolate genome into FASTQ format (at 10X coverage of the original genome) and combining it with a randomly selected gut metagenome from a human individual without cholera. We then mapped these mock metagenomes to a consensus clonal frame constructed from all L2 isolates and calculated pairwise distances between the mock metagenomes using the same method and parameters as described in the manuscript. We found that the L2 isolates from the 7th pandemic formed a distinct clonal cluster, with a clear divergence gap separating them from non-L2 isolates (**Figure R3.2.1**). These results demonstrate that our method is capable of recovering known, pathogenic genome-wide selective sweeps.

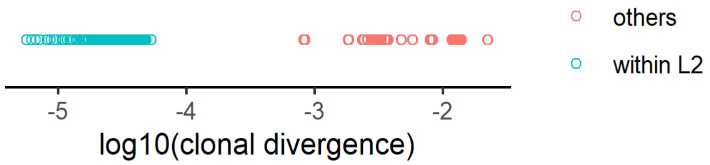

Figure R3.2.1 L2 lineage identified as a distinct clonal GWSS cluster with a clear divergence gap from non-L2 isolates. Each dot represents a mock metagenome generated from a V. cholerae isolate genome.

2. **Validation of different waves of the 7th pandemic and their ages**. The 7th cholera pandemic is characterized by three successive global waves. The first wave, which began in 1961, was eventually replaced by strains from the second wave in the late 1970s, and the third wave in the early 1990s. While descendants from the second and third wave are both still circulating today, no descendants from the first wave have been isolated after 1992. This lineage can thus be considered as extinct, serving as a well-documented example of a gradually extending "broom branch."

Accordingly, the age of the 7th pandemic selective sweep can be approximated to be around 2025-1980=45 years old, while the third wave is estimated to be approximately 2025-1990=35 years old. Consistent with this timeline, our method of calculating pairwise distances between metagenomes using a consensus clonal frame and molecular clock rate of 1–10 SNPs per genome per year, estimated the age of the entire 7th pandemic sweep to range from 5.1 to 51.5 years, and the age of the third wave to range from 4.6 to 46 years. The neighbor joining tree constructed using pairwise distances also shows that waves 2 and 3 appear as discrete clusters, with the wave 3 cluster emerging out of the wave 2 cluster (**Figure R3.2.2**).

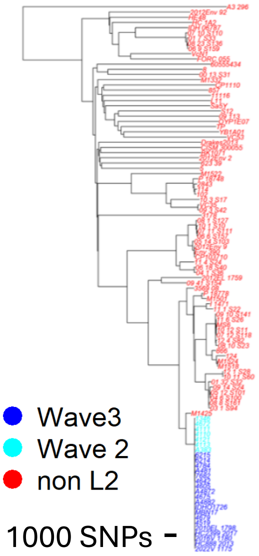

Figure R3.2.1 Neighbor-joining tree constructed from pairwise distances among mock metagenomes derived from isolates within the L2 lineage and from non-L2 lineages.

Actions taken:

Main text: We summarize the content above as part of the section "**Validation of GWSS detection and age with pathogen datasets**" (Lines 854-873).

Supplementary text and Figures: The content above is included as supplementary text, while Figures R3.2.1 and R3.2.2 are added to the manuscript as Extended Data Fig. 10.

Rev3-Comment 3.3. *A major issue arises in that the authors use a majority nucleotide (consensus clonal frame) approach to identify strains, but these may not be phased accordingly with one another. Specific SNPs may have converged because of function, but represent convergence. The authors should validate their use of consensus SNPs in a rigorous way to prove that it is not biasing their results.*

Response: Given that our pipeline does not rely on extracting strains from metagenomes but uses consensus clonal frames (CCFs) extracted from isolate genomes and mapping metagenomic reads to them, the identification of GWSSs is ultimately based on pairwise distance comparisons between samples (metagenome–isolate, metagenome–metagenome, and isolate–isolate), with the CCF only serving as a reference. Therefore, we believe that the use of consensus clonal frames is unlikely to introduce significant bias into our GWSS detection. Also, since our CCFs are extracted from isolate-based GWSSs that are predominantly vertically inherited, we believe that the majority of consensus SNPs reflect vertical inheritance, and that those arising from functional convergence represent only a very minor fraction. Nonetheless, we agree with the reviewer that validating the use of consensus SNPs would enhance the rigor of our approach. We therefore use the same *V. cholerae* dataset as above to ask if using the consensus clonal frame as the representative for the L2 lineage would affect the identification or characterization of sweep age, when compared to using clonal frames derived from 10 randomly sampled L2 isolates.

We found that the difference was minimal: the maximum pairwise distance within the entire L2 lineage (representing the 7th pandemic) calculated using the consensus clonal frame was 103 SNPs, corresponding to an estimated sweep age of 5.1 to 51.5 years. In comparison, the maximum pairwise distance calculated using 10 randomly sampled L2 isolates as references was 116 ± 4.6 SNPs, corresponding to a sweep age of 5.8 to 58 years. Thus, any potential bias introduced by using the consensus clonal frame is negligible, particularly given that our goal is to estimate sweep age within the correct order of magnitude.

Action taken: We summarize the content above and add it to the section "**Validation of GWSS detection and age with pathogen datasets**" (Lines 874-883) in the **main text**.

Rev3-Comment 3.4. *Does the abundance of genomes in samples affect their observations?*

Response: We agree that the abundance of genomes in metagenomic samples can affect our observations. Because we require a minimum coverage depth of 2X and at least 25% coverage breadth for a sample to map to a consensus clonal frame (CCF, and thus receive an SGB assignment and sample-to-CCF distance), it is likely that we miss GWSSs from SGBs that are relatively rare (i.e., SGBs present at less than ~0.04%–0.12% relative abundance in an average-sized human gut metagenome of 5–15 Gb). Conversely, it is also possible that some apparent "gaps" between clusters could be false positives if certain genomes in metagenomes are not observed because they are present at low abundance and therefore fall below our detection thresholds. However, we found that modifying the coverage depth cutoff (from 2X to 1X or 4X), the coverage breadth threshold (from 25% to 50% in metagenomes or from 50% to 25% in isolates), or the required coverage overlap between samples (from 25% to 50%) resulted in only minor changes (1-3%) in the number of GWSSs detected (**Figure R2.1.3**). Therefore, within the detectable abundance range, coverage/breadth thresholds have only a modest effect on GWSS

detection. Of course, it remains possible that genomes present at very low abundances in metagenomes could be missed entirely; however, such strains would likely function more like a seed bank and, in practice, could be considered effectively absent since they are outcompeted by other, more abundant strains (in fact, this could also indicate that they are in the process of being wiped out by an ongoing sweep) , especially when these genomes are from SGBs that generally persist at relatively high abundance in the gut.

Actions taken: How coverage thresholds could affect the detection of strains have been included in the main text "**Robustness of GWSS assignments**" (lines 781-795) as well as **Supplementary Data Fig.2c** and **Table S8**.

Rev3-Comment 3.5. *How does the number of samples with the SGB affect the likelihood of calling a sweep or the timing of the perceived sweep?*

Response: In lines 129-131 of the original manuscript (now lines 131-134), we reported that the number of GWSSs detected in each SGB significantly correlates with the total number of samples (isolates + metagenomes) where the SGB is detectable (Spearman's test, n=46, Rho=0.46, p=0.001). We interpreted this as a signal that it is likely that GWSSs exist in other gut bacterial families that have been less extensively cultured. We also agree that the correlation between the number of samples within an SGB and the estimated timing of the GWSS is an interesting question to explore. To address this, we tested the correlation between the number of samples within each SGB and the average maximum SNP distance among GWSSs within that SGB, and found no significant relationship (Spearman's test, n = 46, p = 0.91), indicating that we are not systematically missing older sweeps because of under sampling.

Rev3-Comment 3.6. *The molecular clock analysis assumes that all organisms have the same mutation rate, whereas some organisms are more actively dividing and maintaining more diversity within their populations. Treating each genome bin with the same molecular clock analysis seems inappropriate.*

Response: While we agree with the reviewer (and Reviewer 2) that different organisms have different mutation rates and had hoped to find more taxa specific molecular clocks, this type of data was scarcer than anticipated. The estimate of 1-10 SNPs per genome per year is based on the currently available literature[1–4] that estimated point mutation rates for bacteria in a rigorous, isolate-based manner. Even so, most of these estimates come from pathogenic bacteria, and the only estimate made for gut commensal bacteria is *Bacteroides fragilis* (1 SNP per genome per year).

To make up for this, we **included in the paper an estimated metagenomic molecular clock for 9 SGBs based on all available metagenomic time series data or twin metagenomes we could access**. These molecular clocks were estimated by identifying strains that persisted in individuals over time or were shared between twins, then calculating the number of SNPs separating these strains divided by the years between sampling (or the time twins had been living apart, under the assumption that they initially shared the same strains).However, the taxa we were able to investigate in this manner were limited to those with sufficient coverage and detectable shared strains across time points (twin samples), and was thus limited to 9 SGBs. Overall, these estimates were consistent with the range of 1–10 SNPs per genome per year (Figure 2b). Although we hope that future work will expand this analysis to more SGBs and integrate both isolate-based and metagenome-based molecular clocks, current efforts remain constrained by the availability of suitable isolate genomes and longitudinal metagenome data. Therefore, **in the paper we focus on estimating GWSS ages within the correct order of magnitude rather than to derive precise ages to circumvent this limitation**.

<u>Action taken</u>: We now emphasize that our focus is on estimating GWSS ages within the correct order of magnitude rather than deriving precise dates, due to limitations in data availibilty in lines 174-176 of the **main text**.

Rev3-Comment 3.7. *They cite genes that many bins show strong signs of positive selection (lines 137-138), but are only able to discuss the capsular polysaccharide genes (lines 240) as possibly being selective. What are the genes that the authors find? Are there any logical observations there (for pathogens, perhaps)?*

<u>Response</u>: We thank the reviewer for this comment. We agree that a more comprehensive analysis of genes that are unique to, or under positive selection in, GWSSs is necessary and would strengthen the paper. We therefore analyzed whether these genes across all the GWSSs we identified are more enriched for certain functional categories (COG and PFAM) compared to all genes present in the GWSSs for each SGB. We found that, in commensals, these genes are more enriched in COG category M (cell wall and membrane biogenesis) and glycosyltransferases, suggesting that the use of capsular polysaccharides to adapt to varying host phenotypes related to health or disease may be a common strategy among commensal bacteria. Meanwhile, for the pathogen GWSS clusters in our analysis, we found that genes unique to, or under positive selection within these clusters, often include virulence factors, such as effector delivery systems, virulence enhancers (e.g., adhesins and siderophores), and enterotoxins in *Salmonella enterica* and *E. coli.* Because the latter contains the expected gene categories for pathogens, this result builds confidence in the identification of specific gene categories for the commensal GWSSs.

<u>Actions taken</u>:

Main text: We have revised the section "**GWSS clusters represent ecologically differentiated populations**" (lines 241-255) so that it now includes a systematic analysis of how genes that are unique to, or under positive selection in GWSSs are more enriched for certain functional categories (COG and PFAM) compared to all genes present in the GWSSs for each SGB. We also discuss how this enrichment suggests that the use of capsular polysaccharides to adapt to varying host phenotypes related to health or disease may be a common strategy among commensal bacteria, particularly in the *Bacteroidales*. We have also updated the methods section "**Identification of sweep-specific genes**" (lines 994-1008) accordingly.

Main Figures: We now include in Figure 4c a bar plot showing the enrichment of genes involved in cell wall and membrane biogenesis, as well as glycosyltransferases, among genes unique to GWSSs relative to their abundance in all GWSSs, in three *Bacteoridales* SGBs. We also demonstrate that this enrichment is independent of disease associations.

Supplementry tables and figures: We have updated Supplementary Table S3, so that it now includes genes and functional assignments that are unique to, or under positive selection in all commensal and pathogenic SGBs. We have also updated Extended Data Fig. 9 to include a bar plot showing the functional enrichment of these genes in each commensal SGB.

Rev3-Comment 3.8. *The authors cite genomes that have swept between isolated populations (line 153). How many years do they estimate that this particular sweep occurred?*

<u>Response</u>: In line 181-184 of the previous manuscript (now lines 165-167) , we had estimated this particular genome-wide selective sweep, which is the oldest GWSS in detected in SGB1836 (*Bacteroides uniformis),* to have an age of approximately 100-1000 years, suggesting that this cluster may have spread prior to industrialization.

Rev3-Comment 3.9. *The authors filter out a lot of sweeps and also samples (Methods). It would help to understand how many samples/sweeps per SGB were being filtered for what*

reasons and whether any of these filters strongly influences their results. Removing certain samples with added diversity may make the remaining diversity seem more closely related.

Response: There are two major reasons for filtering out certain samples. First, we only include one sample from each individual (or family) because repeated sampling of the same person, or short-term transmission among family members due to close contact, can create artificial signals of GWSSs. Indeed, while this approach may not capture all within-person diversity, especially if multiple strains coexist in an individual, there is no evidence that strains coexisting within one person cannot also be found in other individuals. In other words, with sufficiently large sampling across unrelated individuals, we expect that this diversity will still be captured.

The second major reason we filter samples is to exclude metagenomes that contain multiple strains within the same SGB. Without this filter, mixed-strain samples could generate artificial composite genomes when calculating distances to the consensus clonal frame (CCF)[8,9]. For example, if a metagenome contains two strains (one from within a GWSS and one from outside it) the resulting signal can appear as a single intermediate strain that falls within the (>5X) distance gap that is used to define the GWSS, increasing the risk of false negatives. We recognize that this filter may also remove some strain diversity, but again, there is no evidence that strains coexisting in one person's gut are always found together elsewhere or cannot be detected individually. In a preliminary analysis of our 1,477 metagenomes, for example, we found that of the 118 strains of *Bacteroides uniformis* detected, 54 were present both in mixed-strain and single-strain samples, and we expect this number to increase with a larger dataset. This suggests that excluding mixed-strain samples does not systematically remove strains that cannot be found as single-strains in other hosts.

Actions taken:

Main text: We now report in the relevant methods section that these filters reduce the number of usable metagenome samples per SGB to between 1 and 742 (lines 734-736).

Supplementary tables: We also now include the number of usable metagenome samples and isolate samples for each SGB in Table S7.

Rev3-Comment 3.10. *I would ask the authors to help align the two notions that these sweeps occur, sometimes over rapid periods (10 years) but that there is not a very strong signal of organismal sharing (within population sharing is extremely low—data coming straight from Segata's previous Nature paper).*

Response: We thank the reviewer for this comment, as it highlights exactly what we hope to draw attention to with this paper. Much of the existing human gut microbiome literature has focused on transmission, emphasizing that strain sharing is more common among family members than among unrelated individuals. However, a closer examination of the Segata paper shows that sharing among unrelated individuals is not negligible — approximately 3% of individuals share at least one strain[10]. This is consistent with other studies of strain transmission events, which report that strain sharing among unrelated individuals ranges from ~0.1% to 2%, which is much larger than neutral simulations[9,11], and is likely the result of broader population genetic processes rather than unrecognized direct transmission.

It is also important to note that our GWSS detection approach does not rely on a fixed divergence threshold (as in Garud *et al.*[9], where strains are considered the same if d < 0.01%) or use within-host strain diversity as a benchmark for defining strain sharing[10]. Instead, our method detects genome-wide selective sweeps based on discontinuities in divergence, meaning that strains within the same GWSS cluster in commensal gut bacteria can be more divergent than strains identified by simple transmission-focused thresholds (average GWSS

divergence is 0.1% ± 0.1%, with a maximum up to 0.6%). These numbers are therefore consistent with our finding that, on average, about 2% of our samples are contained within GWSS clusters.

We would also like to emphasize that low pairwise strain sharing does not preclude the presence of strains capable of spreading rapidly across hosts. In fact, given that GWSSs affect only a subset of strains within a subset of gut microbiome species, it is entirely consistent to observe hundreds of clonally expanding strains in a human population while still maintaining a low baseline of pairwise strain sharing. This is particularly true if such clonal populations reach only modest prevalence, e.g., sweeping to 5–10% of hosts rather than fixing across the entire population. In other words, widespread but incomplete sweeps are compatible with low overall pairwise sharing rates, especially when considered across a large number of species and populations.

Our paper specifically aims to draw attention to the fact that a low level of strain sharing does not imply randomness; rather, it can reflect non-neutral processes shaped by shared selective forces among unrelated individuals. While previous studies have acknowledged that strain sharing among unrelated individuals can result from eco-evolutionary mechanisms and that host adaptation may play a role, they have not explicitly examined this sharing as a signature of common selective pressures acting across hosts. Here, we provide additional evidence that transmission among individuals is non-neutral and propose a mechanism by which this strain sharing can arise from ecological conditions shared among globally distributed human populations.

Action taken: We have now added a sentence in the **discussion** part of the **main text** (lines 275-279) to emphasize that despite the level of strain sharing among unrelated individuals is lower than that of related individuals, it is not random.

Rev3-Comment 3.11. Understandably, the timing of the selective sweeps may have occurred 10-10,000 years ago, by these authors' calculations, but the authors could also examine sharing of specific organisms and also calculate whether the number of SNPs expected matches the distribution of SNPs that occur (keeping in mind again, that the majority SNP is likely not a single bona fide strain). These two observations (recent sweeps and the evident, albeit low within population sharing) seem contradictory. Without more proof (many more isolate genomes, long-read sequencing, or corroborating evidence dating these observations), the results seem suspect.

Response & actions taken: We appreciate the reviewer's concern and agree that additional corroborating evidence would strengthen these findings. We believe that Figure 2b, together with our new benchmarking analysis using *Vibrio cholerae*, helps address this point. The estimated GWSS ages based on metagenomic molecular clocks in Figure 2b already cover all nine SGBs for which we could detect shared strains with sufficient coverage in available metagenomic time series or twin metagenomes. While we acknowledge that expanding this analysis to more SGBs would be valuable, this is currently limited by the availability of suitable isolate genomes and longitudinal metagenomic datasets. However, for pathogens like *Vibrio cholerae*, where historical outbreak data exist, we were able to benchmark our sweep age estimates against known outbreak dates for the 7th pandemic and its waves, finding that the true dates fall within our estimated ranges. We have now included this benchmarking analysis in the Methods and Supplementary Text. Therefore, we believe that our current approach is both sufficient and up to date given available data, although we recognize there is still room for improvement as more isolate genomes and metagenomes become available.

Rev3-Comment 3.12. *It is unclear what is being shown in Figure 3, and to some extent Figure 4. They show results but now rhyme or reason as to why we these are accurate.*

<u>Response and actions taken</u>: We thank the reviewer for this comment and recognize that the information in Figure 3 may have been confusing. We have now revised the figure to use a histogram, which more clearly illustrates the differences and distribution of recombination rates between SGBs with GWSSs and those without. Additionally, we now present the *Blautia* data as a separate subfigure to help readers interpret the information more easily. To further validate our results, we benchmarked our recombination rate estimates in Figure 3 against those reported by Liu *et al*. (2024)[11], who used a similar approach (fraction of recombined genome per synonymous SNP in clonal fractions) but with a different set of metagenomes (932 samples) mapped to species-specific core genomes. For 10 out of 11 SGBs shared between our datasets, our estimated recombination rates are within 0.5- to 3-fold of those reported by Liu *et al*. (Figure R3.12.1). Considering that our estimated recombination rates among different SGBs span three orders of magnitude, and that Liu *et al.* used more evolutionarily conserved genome regions and synonymous clonal divergence (whereas we used overall clonal divergence), this level of agreement is reasonable and supports the accuracy of our analysis. In addition, Figure 4 has been substantially revised following the expansion of the ecological association analyses, and we hope it is now clearer and easier to interpret.

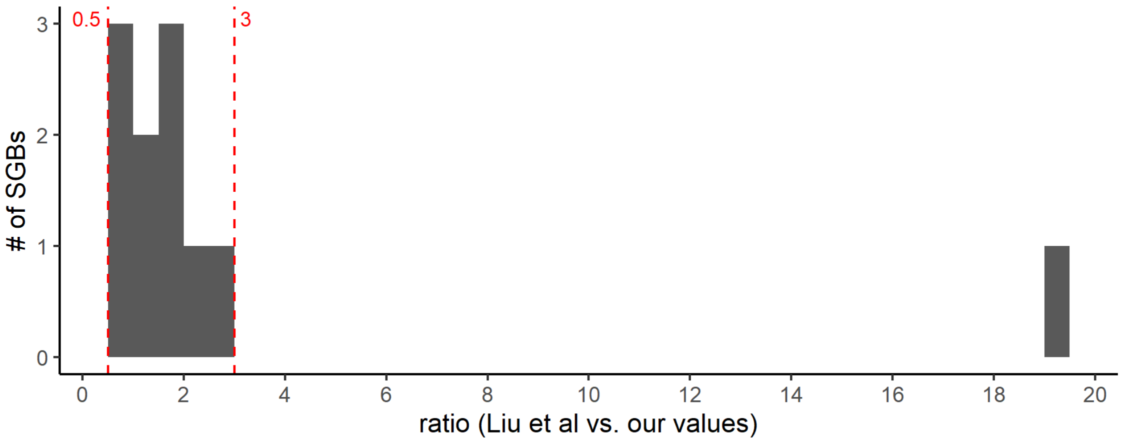

Figure R3.12.1 Histogram of the ratio of recombination rates estimated using Liu et al. (2024) data versus our dataset for the 11 overlapping SGBs.

Minor comments:

Rev3-Comment 3.13. *The curatedmetagenome project has over 10,000 metagenomes. Why did the authors only use less than 1500?*

<u>Response</u>: The 1,477 metagenomes we use are representative of 74 datasets that cover all the >10,000 metagenomes in the curatedmetagenome project, so that we cover a suffient range of ages, diseases and biogeographies. As also written in Table R2.1.1 and Figure R2.1.2, to ensure that the number of GWSSs detected was not underestimated due to an insufficient number of metagenomes, we assessed whether GWSS detection was sensitive to the number of metagenomes used. Specifically, we performed a rarefaction analysis by subsampling the full set of 1,477 metagenomes used in the study to 0%, 10%, 20%, 40%, 60%, and 80%, and then counted the total number of GWSSs detected at each level. The rarefaction curve in Figure R2.1.2 shows the number of GWSSs detected increases with the number of metagenomes included, but this trend saturates between 20–40% of the currently used set of metagenomes. These results suggest that the number of metagenomes used in the current analysis is already sufficient to recover all GWSSs detectable with the available isolate genomes. While additional GWSSs may exist, their discovery is likely limited by the availability of isolate genomes rather than by metagenome sample size.

Actions taken:

Main text: This rarefaction analysis has been summarized and included as part of an additional section in methods called the "**Robustness of GWSS assignments**" (lines 781-795).

Supplementary tables and Figures: This rarefaction analysis has been described in more detail in Table S8, while Figure R2.1.2 has been added to the manuscript as Supplementary Data Fig. 2b.

Rev3-Comment 3.14. *Please explain what "6% diverged" refers to— is this differences in ANI of the entire metagenomic bin? (line 93)*

Response and action taken: Thank you, we should have explained this better. The 6% diverged here means 6% diverged from the reference genome, therefore in some SGBs the maximum divergence could be up to 10-12%. We added the corresponding explaination "As a result, the total divergence within each SGB could be as high as 10–12%." in lines 538-539 of the **main text**.

Rev3-Comment 3.15. *Seniority means age? This is a confusing usage of this word.*

Response & action taken: Thank you, we have now changed seniority to advanced age (>65) in the corresponding line (now line 218) and also throughout the paper.

Rev3-Comment 3.16. *Is the Yersinia pathogen in people's microbiomes a misannotation?*

Response: The major Yersinia SGB detected in our analysis is *Yersinia enterocolitica*, which is a well-known foodborne pathogen that causes diarrhea, most commonly acquired through the consumption of raw or undercooked pork (https://www.cdc.gov/yersinia/about/index.html). Therefore, we do not believe that the presence of Yersinia in people's microbiomes reflects a misannotation.

References

1. Didelot, X., Walker, A. S., Peto, T. E., Crook, D. W. & Wilson, D. J. Within-host evolution of bacterial pathogens. *Nat. Rev. Microbiol.* **14**, 150–162 (2016).
2. Zhao, S. *et al.* Adaptive Evolution within Gut Microbiomes of Healthy People. *Cell Host Microbe* **25**, 656-667.e8 (2019).
3. Duchêne, S. *et al.* Genome-scale rates of evolutionary change in bacteria. *Microb. Genomics* **2**, e000094 (2016).
4. Mutreja, A. *et al.* Evidence for several waves of global transmission in the seventh cholera pandemic. *Nature* **477**, 462–465 (2011).
5. Tajima, F. Statistical method for testing the neutral mutation hypothesis by DNA polymorphism. *Genetics* **123**, 585–595 (1989).
6. Nielsen, R. Statistical tests of selective neutrality in the age of genomics. *Heredity* **86**, 641–647 (2001).
7. Wang, H. *et al.* Genomic epidemiology of Vibrio cholerae reveals the regional and global spread of two epidemic non-toxigenic lineages. *PLoS Negl. Trop. Dis.* **14**, e0008046 (2020).
8. Truong, D. T., Tett, A., Pasolli, E., Huttenhower, C. & Segata, N. Microbial strain-level population structure and genetic diversity from metagenomes. *Genome Res.* **27**, (2017).
9. Garud, N. R., Good, B. H., Hallatschek, O. & Pollard, K. S. Evolutionary dynamics of bacteria in the gut microbiome within and across hosts. *PLOS Biol.* **17**, e3000102 (2019).
10. Valles-Colomer, M. *et al.* The person-to-person transmission landscape of the gut and oral microbiomes. *Nature* 1–11 (2023) doi:10.1038/s41586-022-05620-1.
11. Liu, Z. & Good, B. H. Dynamics of bacterial recombination in the human gut microbiome. *PLOS Biol.* **22**, e3002472 (2024).

Comments to the editor:

We would like to thank the reviewers for providing more constructive feedback. We have now revised the manuscript according to the comments. Specifically, we have addressed Reviewer #2's request to validate combinations of parameters used for genome-wide selective sweep (GWSS) assignments, and clarified why genes under positive selection are not enriched in GWSSs (Reviewer #1). We feel that these changes have helped improve the rigor of the manuscript.

Below you will find a point-by-point response to the questions from the reviewers (responses in regular text, comments from reviewers in *italic*). For clarity, we also included a copy of the manuscript where all changes in the text have been marked in red ("related manuscript file").

Referees' comments:

Referee #1 (Remarks to the Author):

The authors have done a great job responding to my prior comments.

Rev1-Comment1.1. *My only remaining minor comment is that I am having some conceptual trouble with the result showing that genes under positive selection are not enriched in GWSS clusters relative to non-GWSS clusters. Can the authors provide some intuition for this result? I would expect sweeps within the clonal frame to be driven by selection for specific genes/traits within that frame, and that this selection would be detectable (e.g., in terms of dN/dS). Are GWSSs driven by selection acting on the non-clonal frame? Can the authors explain the balance of forces that would drive a global GWSS signature without showing an enrichment for selection effects at specific loci?*

Response: We thank the reviewer for raising this conceptual point. We believe a GWSS need not show an excess of genes with dN/dS>1 for several reasons. First, a sweep can be driven by one or a few variants while the rest of the clonal frame hitchhikes. With limited recombination, most genes are neutral passengers, so a single gene under strong selection can lead to genome-wide sweeps, while multiple genes under weak selection may not. Second, the targets of selection may be genes unique to a GWSS, structural variants, or regulatory changes (e.g., promoters, phase variation, inversions, mobile elements) rather than coding substitutions. Third, GWSS often reflect recent, rapid events: once the adaptive haplotype fixes and competitor lineages are purged, the sweeping strain can persist even if the selective force leading to the sweep disappears. Therefore, the driver locus can return to be under neutral selection, and over time, may no longer maintain a dN/dS>1.

Although the number of genes under positive selection is not an appropriate signal indicating GWSSs, we can still compare the GWSS clade with its sister clade to pinpoint potential drivers of a sweep. We do this by looking for genes that are exclusive to the GWSS (which thus do not have a dN/dS signature), or that show a signature of positive selection that is specific to the GWSS. These results are presented in Table S3, Figure 4c, Extended Data Fig. 9 and lines 241-255.

Referee #1 (Remarks on code availability):

The code provided appears adequate for reproducing the authors' analyses.

Referee #2 (Remarks to the Author):

I appreciate the authors' extensive efforts to address the parameter sensitivity concerns through a comprehensive robustness analysis. The detailed justification for each cutoff and the systematic evaluation of parameter variations are helpful and represent a major improvement to the manuscript. However, I have remaining concerns:

Rev2-Comment 2.1. *Regarding parameter sensitivity (Comment 2.1): It is good to see that individual parameter variations generally result in modest changes (1-20%) to GWSS detection. Nevertheless, I remain concerned about the impact of these parameter choices. Two important questions:*

First, regarding downstream biological implications: Do the 10-20% differences in GWSS numbers translate to meaningful changes in the key biological conclusions? Is it possible that these 10-20% GWSS are different (plausible, considering that they were not detected initially so they may have different properties) and including them can lead to different results? Do the results and conclusions remain consistent across parameter sets? I would encourage examining whether the primary findings are robust to these parameter variations.

Second, regarding combinatorial effects: Although individual parameter changes show modest effects, the cumulative impact of multiple parameter adjustments could be more substantial. Testing a few combinations of parameter changes would strengthen confidence in the overall approach.

Response and actions: We thank the reviewer for suggesting these additional robustness checks. We evaluated combinatorial effects of parameter changes by testing 54 parameter combinations spanning all stages of the GWSS-calling pipeline. We found that the total number of GWSSs always varies by <15%, and that the major biological conclusions hold. These are that sweeps have a wide age and geographical distribution (with a large fraction being younger than a century, indicating rapid spread), and that genes specific to GWSS tend to be on mobile genetic elements and enriched in functions involved in the synthesis or modification of glycans and glycoconjugates.

Below, we explain the 54 parameter combinations that span all stages of the GWSS pipeline (3×3×3×2), and have the results summarized in **Figure R2.1.1**:

(i) We varied the pairwise recombination threshold used to define initial isolate-based putative GWSSs (33%, 50%, 67%). While 50% was the parameter used in the manuscript, in prior tests using the 67% cutoff recovered 2.4% more GWSSs (and 15% more constituent isolate genomes), whereas 33% reduced GWSS recovery by 11.9% (and 12.9% less constituent isolate genomes, Supplementary Data Fig. 2a).

(ii) In the GWSS confirmation step, where isolate and metagenome samples are mapped to the clonal consensus frame (CCF) of the putative GWSSs and the 5X rule is applied to sample-CCF distances, we varied the minimum mean coverage depth required for inclusion of the sample (1X, 2X, 4X).

(iii) Also in this mapping step, we varied coverage breadth thresholds with three isolate/metagenome settings: Isolates 25% / MG 25%, Isolates 50% / MG 25%, and Isolates 50% / MG 50%.

(iv) Since our final confirmation step for GWSSs were based on computing pairwise distances between samples mapping to the same CCF and reapplying the 5X rule, we altered the minimum shared covered fraction required for distance calculation (>25% or >50%) in this step.

We rebuilt the CCF database to include all CCFs identified from isolate-based putative GWSSs under each recombination cutoff (33%, 50%, 67%). For every parameter set, we then re-ran the full pipeline and recorded the total number of GWSSs detected.

We include a figure demonstrating how different parameter combinations affect GWSS calling (**Figure R2.1.1a**). The dominant driver was the recombination cutoff used to generate isolate-based putative sweeps. Using 33% reduced the number of putative sweeps by 11.9% and propagated to a <15% decrease in final GWSS counts. Using 67% yielded 2.4% more putative sweeps and introduced <7% additional GWSSs. In contrast, varying coverage depth, coverage breadth, and pairwise coverage thresholds produced only minor amplifications of this initial effect. As we argued in our previous revisions, we believe the 50% cutoff is the best compromise between losing a small number of GWSS while allowing confident construction of the clonal frame (and therefore accurate mapping and downstream distance calculations).

Furthermore, none of the parameter sets led to a significant change in the age distribution of the sweeps using a constant molecular clock of 5 SNPs per genome per year (**Figure R2.1.2**). The number of (young) sweeps estimated to be less than a century old varied between 31-40 (**Figure R2.1.1b**).

Similarly, the functional categories that were enriched in the largest number of SGBs and are shown in **Figure 4C**, including COG category M and PFAMs glycosyl transferases, were stably enriched across all parameter combinations (**Figure R2.1.3**). This was also true for functional categories associated with mobile genetic elements, including COG category L and PFAMs associated with phage integrases. Two PFAMs that were enriched in fewer SGBs were found to be unstable across the parameter combinations, but these were not highlighted in the paper. Two additional PFAMs were detected in some of the new parameter combinations, but as these are also not stable across all combinations, they are not presented in the revised paper.

We have now **included Figure R2.1.1 as Supplementary Figure 2c** in the manuscript, **updated Table S8 (with data corresponding to Figures R2.1.1 and R2.1.3) and lines 741-745 in the methods section "Robustness of GWSS assignments"** to assess the effect of parameter combinations on GWSS calling, rather than individual parameters alone.

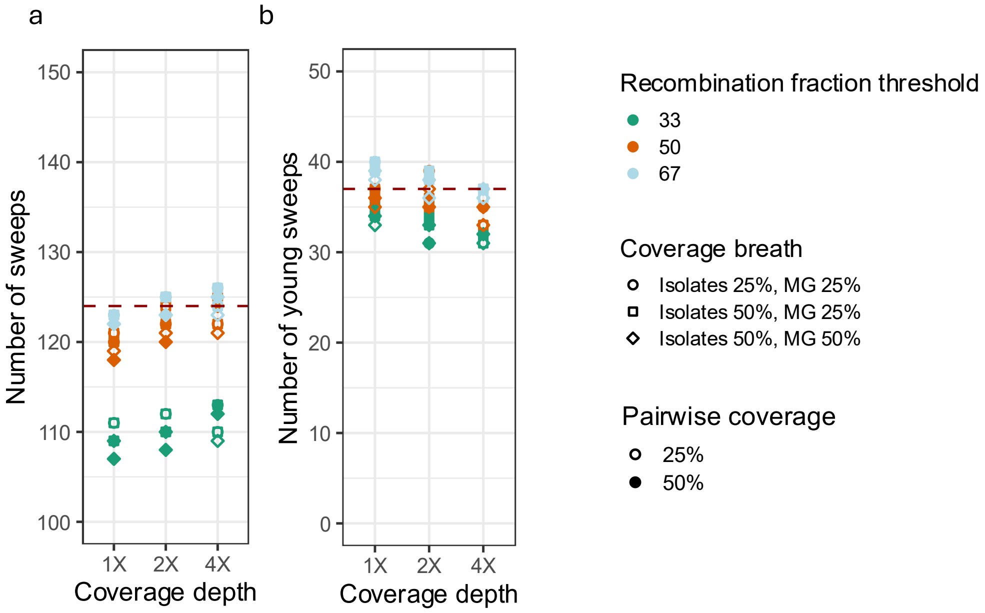

Figure R2.1.1 – The number of a) total and b) young (<100 years old) GWSSs is robust to 54 combinations of parameter changes. Each dot represents the number of GWSSs detected under one set of parameters, with the dot color representing the pairwise recombination

threshold for the identification of the initial isolate-based sweep (33, 50, 67%). The x-axis shows the coverage depth (1X, 2X, 4X) used for calculating the distance between a (isolate or metagenome) sample and the CCF of the isolate-based sweeps: these numbers set different minimum mean coverages required for a sample to be included in the calculation. Simultaneously, coverage breadth was varied with three settings for isolates and metagenomes (MG): Isolates 25%, MG 25%, Isolates 50%, MG 25%, or Isolates 50%, MG 50%. Finally, "Pairwise coverage" is the minimum shared covered fraction between two samples mapping to the same CCF when computing pairwise distances, set to >25% or >50%. The red horizontal lines represent the number of GWSSs identified with the parameters used in the paper.

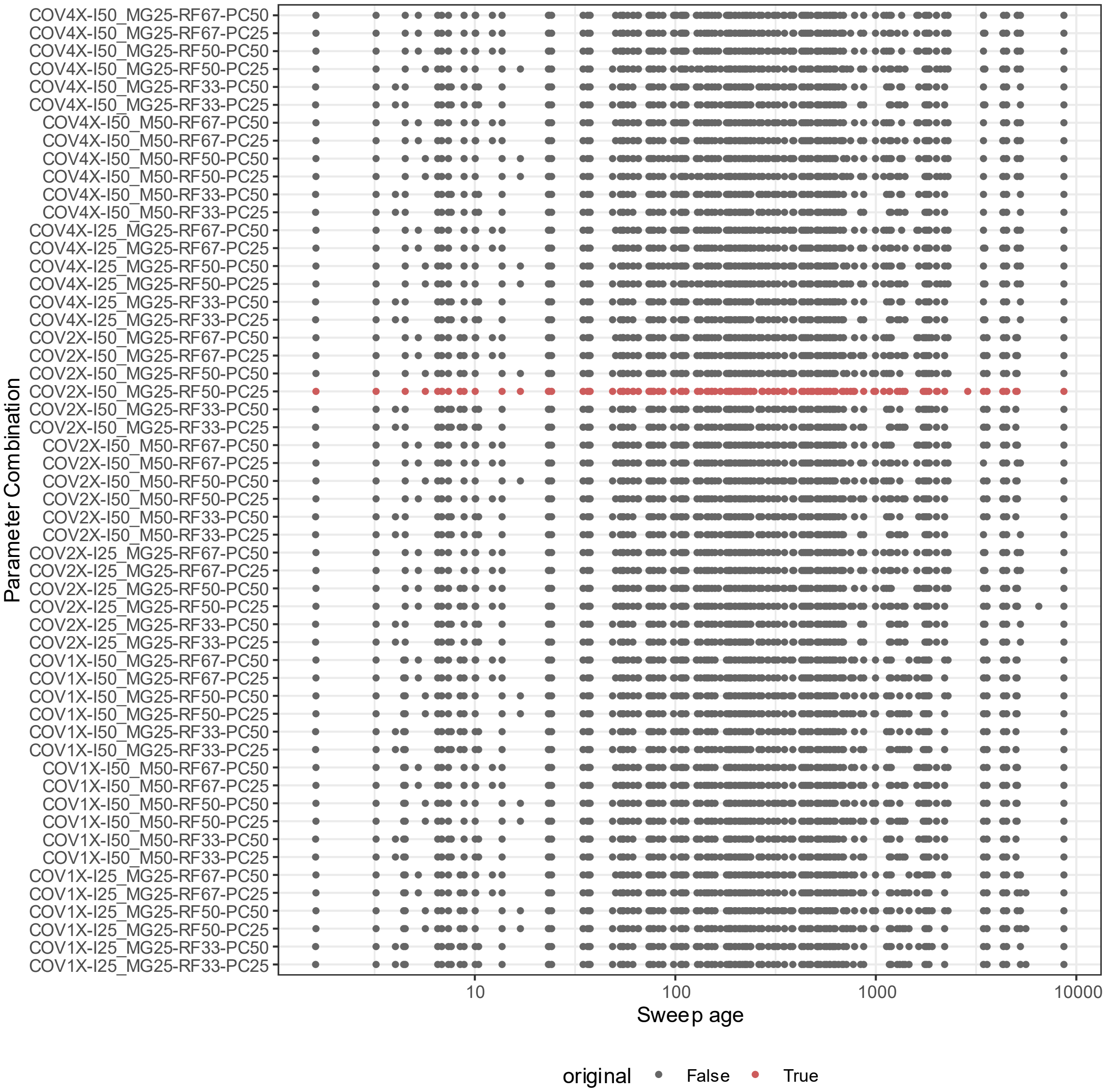

Figure R2.2.2 Age distributions of GWSSs identified under 54 different parameter combinations are highly similar. Each dot represents a GWSS. The dots in red represent the age distribution of the GWSSs using the parameters used in the manuscript. Abbreviations: COV = coverage depth (1X, 2X, 4X); I = Isolate (25: 25% coverage breadth or 50: 50% coverage breadth); MG=metagenome (25: 25% coverage breadth or 50: 50% coverage breadth); RF =

recombination fraction (33: <33%, 50: <50%, 67: <67%); PC = pairwise coverage (25: >25%, 50:>50%).

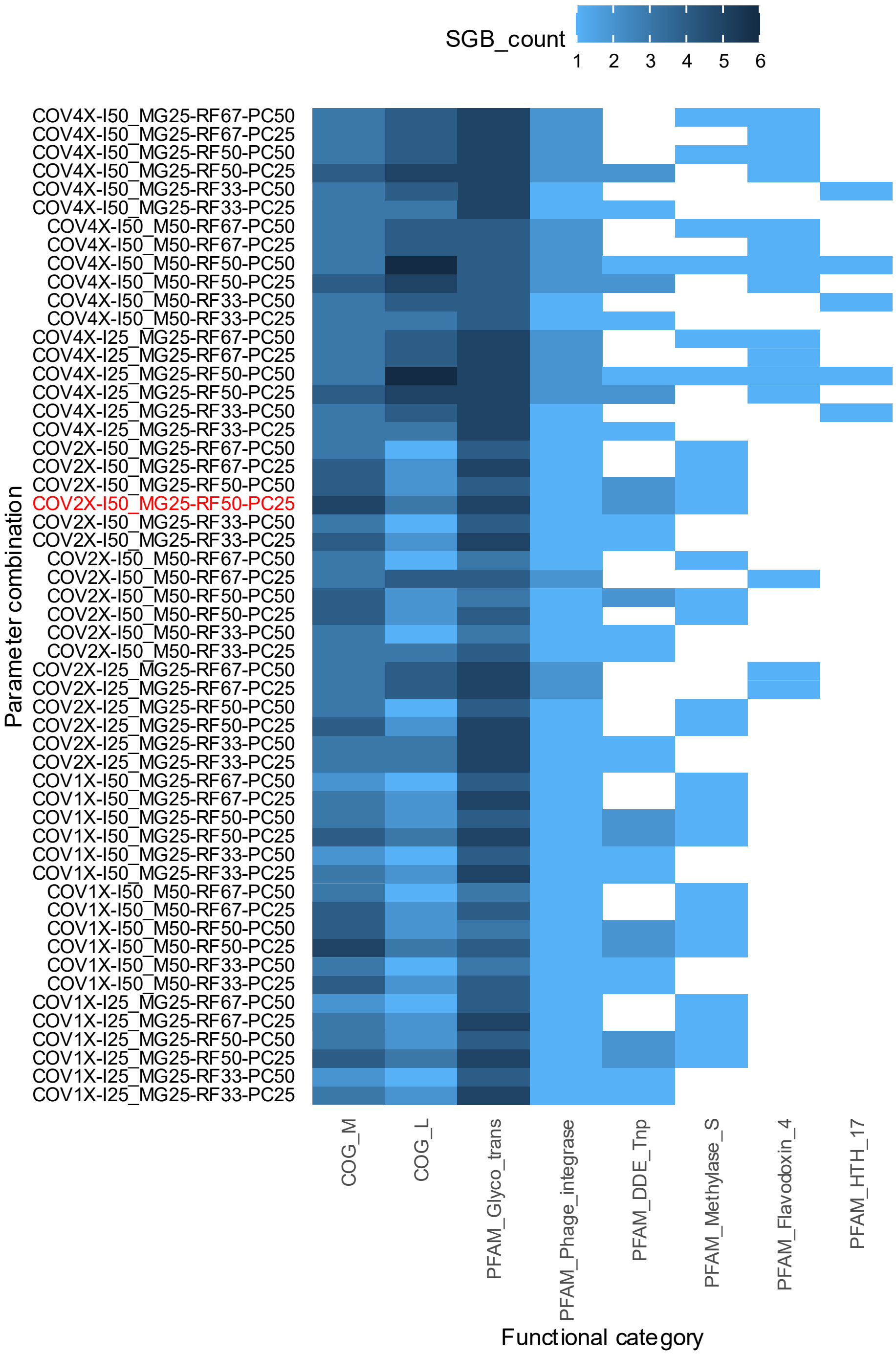

Figure R2.1.3 COG categories or PFAMs enriched under 54 different parameter combinations are highly similar. The color of each tile represents the number of SGBs significantly enriched for a COG category or PFAM in the genes that are unique to GWSSs. The line in red represent the enrichment profile for the parameters used in the manuscript. Abbreviations: COV = coverage depth (1X, 2X, 4X); I = Isolate (25: 25% coverage breadth or 50: 50% coverage breadth); MG=metagenome (25: 25% coverage breadth or 50: 50% coverage breadth); RF = recombination fraction (33: <33%, 50: <50%, 67: <67%); PC = pairwise coverage (25: >25%, 50:>50%).

Rev2-Comment 2.2. *Regarding mutation rate assumptions (Comment 2.2): I understand the practical limitations in obtaining species-specific mutation rates, and I acknowledge the authors' reasonable approach given available data. However, this limitation highlights a fundamental challenge: the biological accuracy of age estimates remains uncertain due to the broad range (1-10 SNPs/genome/year) and limited taxonomic representation of empirical rates. Although the metagenome-based validation for 9 SGBs is valuable, the uncertainty in mutation rates inevitably propagates through all temporal analyses.*

Response: We believe our 1-10 SNPs per genome per year bracket is grounded in measured *in situ* human microbiome data across diverse taxa and methods, including isolate-based studies of commensal gut bacteria, pathogen studies, and metagenome-based validations across 9 SGBs. Reported within-host rates consistently fall in this interval: for example, *Bacteroides fragilis* ≈ 1 SNP/genome/year[1], a non-pathogenic *E. coli* strain ≈ 3 SNPs/genome/year [2], Staphylococcus aureus ≈ 5-8 SNPs/genome/year[3], and Vibrio cholerae outbreaks ≈ 3 SNPs/genome/year[4]. Consistent with these benchmarks, Figure 2b shows that ages estimated from metagenomic molecular clocks for 9 SGBs closely match those estimated with 1-10 SNPs per genome per year, reinforcing the appropriateness of this range. With this empirically supported range in hand, we next clarify how rate uncertainty is handled in our workflow.

First, mutation rates are used only in the final, descriptive dating step to convert SNP distances into approximate ages of GWSSs; there are no downstream temporal analyses that depend on these values. Second, we made sure that all age statements are made at order-of-magnitude resolution (e.g., decades). Consequently, conclusions such as “a large fraction of GWSSs are <100 years old” are robust to the full 1-10 SNPs/genome/year bracket: shifting rates within this range moves absolute dates but does not change their order-of-magnitude placement nor the qualitative inference of recent, rapid spread.

We believe that **Figure 2** and the corresponding text lay out the variation in the age estimates considering the rate brackets observed in the literature and that the conclusions that a global sweep can happen within a lifetime of a human is robust to this variation.

Rev2-Concluding remarks. *The core overall concern here is not methodological inadequacy, but rather the degree to which multiple unavoidable assumptions may compound to influence the biological interpretations. When the entirety of a study's conclusions rest upon a cascade of methodological assumptions and parameter choices (from SNP calling thresholds to recombination cutoffs to mutation rate estimates) demonstrating that results are robust to these choices becomes essential rather than optional. The compounding uncertainty from multiple assumptions might alter our confidence in the biological interpretations. Without understanding how the primary conclusions change when these parameters vary, we cannot distinguish between genuine biological patterns and methodological artifacts.*

Response: Again, we thank the reviewer for pushing us to thoroughly evaluate our parameter choices. We hope that the reviewer agrees that we have demonstrated that the conclusions of the study are robust towards these choices.

Referee #3 (Remarks to the Author):

The responses to reviewers were very thorough. I thank the authors for including the Vibrio analysis and the better explanation of how they use and process the metagenomic data especially vis a vis genome isolates. I have no further questions.

Response: We thank the reviewer for the initial comments, which have helped improve the manuscript.

Citations

1. Zhao, S. *et al.* Adaptive Evolution within Gut Microbiomes of Healthy People. *Cell Host & Microbe* **25**, 656-667.e8 (2019).
2. Ghalayini, M. *et al.* Evolution of a Dominant Natural Isolate of Escherichia coli in the Human Gut over the Course of a Year Suggests a Neutral Evolution with Reduced Effective Population Size. *Appl Environ Microbiol* **84**, e02377-17 (2018).
3. Didelot, X., Walker, A. S., Peto, T. E., Crook, D. W. & Wilson, D. J. Within-host evolution of bacterial pathogens. *Nat Rev Microbiol* **14**, 150–162 (2016).
4. Mutreja, A. *et al.* Evidence for several waves of global transmission in the seventh cholera pandemic. *Nature* **477**, 462–465 (2011).
